# Aspirin-Based Thromboprophylaxis Versus Anticoagulant Regimens After Total Hip or Knee Arthroplasty: An Updated Systematic Review and Meta-Analysis of Randomized Controlled Trials

**DOI:** 10.3390/medicina62102030

**Published:** 2026-10-20

**Authors:** Hasan Al Chalabi, Omar Amro, Amy Gordon, Ghaith Al-Abbasi

**Affiliations:** 1 Northern Ireland Medical and Dental Training Agency (NIMDTA), Belfast BT8 7RL, UK; omar.n.i.amro51@gmail.com; 2 Department of Obstetrics and Gynaecology, North Manchester General Hospital, Manchester University NHS Foundation Trust, Manchester M8 5RB, UK; amy.gordon@mft.nhs.uk; 3 Department of Trauma and Orthopaedic Surgery, NHS Greater Glasgow and Clyde, Glasgow G12 0XH, UK; ghaith.al-abbasi@doctors.org.uk

**Keywords:** aspirin, anticoagulants, deep vein thrombosis, pulmonary embolism, total hip arthroplasty, total knee arthroplasty, thromboprophylaxis, venous thromboembolism

## Abstract

*Background and Objectives*: Aspirin is widely used for venous thromboembolism prophylaxis after total hip arthroplasty (THA) and total knee arthroplasty (TKA); however, uncertainty remains regarding its efficacy and safety compared with anticoagulant regimens. This systematic review and meta-analysis evaluated aspirin-based thromboprophylaxis versus anticoagulant regimens following THA or TKA. *Materials and Methods*: PubMed, Scopus, Web of Science, and the Cochrane Central Register of Controlled Trials (CENTRAL) were searched through July 2026 for randomized controlled trials comparing aspirin-based prophylaxis with anticoagulant regimens. The primary outcomes were any deep vein thrombosis (DVT), symptomatic DVT, and symptomatic venous thromboembolism (VTE). Secondary outcomes included pulmonary embolism (PE), proximal and distal DVT, bleeding outcomes, wound complications, blood transfusion, and mortality. Risk ratios (RRs) with 95% confidence intervals (CIs) were pooled using random-effects models. Additional risk-difference sensitivity analyses incorporated CRISTAL’s published cluster-adjusted estimates. *Results*: Sixteen trials involving 21,265 participants were included. Aspirin was associated with higher risks of symptomatic DVT (RR 1.79, 95% CI 1.35–2.38), symptomatic VTE (RR 1.70, 95% CI 1.33–2.18), PE (RR 1.89, 95% CI 1.33–2.68), and distal DVT (RR 1.47, 95% CI 1.06–2.06). Aspirin was associated with lower risks of minor bleeding (RR 0.47, 95% CI 0.31–0.71) and ecchymosis or bruising (RR 0.60, 95% CI 0.39–0.93). No statistically significant differences were detected for the other assessed outcomes. Additional analyses incorporating CRISTAL’s cluster-adjusted risk differences did not identify statistically significant differences for the three primary outcomes or PE. *Conclusions*: The main pooled analyses associated aspirin-based thromboprophylaxis with higher risks of symptomatic thromboembolic events. However, the symptomatic DVT and symptomatic VTE associations were no longer statistically significant after exclusion of CRISTAL or restriction to trials at low risk of bias. The low-risk analysis identified a significantly higher risk of any DVT with aspirin. Aspirin reduced minor bleeding and bruising without a statistically significant reduction in major or clinically relevant non-major bleeding. These findings require cautious interpretation given dependence on CRISTAL, sensitivity to trial inclusion and analytical approach, and differences in prophylactic strategies and outcome definitions.

## 1. Introduction

Total hip arthroplasty (THA) and total knee arthroplasty (TKA) are highly effective procedures for relieving pain and restoring function in patients with advanced joint disease. However, postoperative venous thromboembolism (VTE), deep-vein thrombosis (DVT) and pulmonary embolism (PE) remain an important preventable cause of morbidity and mortality. Therefore, preventive protocols must be used to avoid these complications. Selection of a prophylactic agent is a challenge as it requires balancing thromboembolic prevention against bleeding [1,2].

Aspirin is an important thromboprophylactic agent because it is inexpensive, orally administered, widely available, and does not require routine laboratory monitoring. However, international recommendations remain inconsistent, reflecting persistent uncertainty in the evidence. The American Society of Hematology conditionally recommends either aspirin or an anticoagulant after THA or TKA, but this recommendation was based on very-low-certainty evidence [2]. The National Institute for Health and Care Excellence recommends low-molecular-weight heparin (LMWH) followed by aspirin as one option after elective hip replacement, whereas aspirin monotherapy is an option after knee replacement [3]. In contrast, the 2022 International Consensus Meeting on VTE strongly supported low-dose aspirin as a prophylactic option in appropriately selected arthroplasty patients [4].

Several randomized controlled trials (RCTs) addressed clinically distinct prophylactic strategies after THA or TKA. In EPCAT I, aspirin was used only after an initial 10-day course of dalteparin following THA and was noninferior to continued dalteparin for extended prophylaxis [5,6]. EPCAT II similarly demonstrated that, after all participants had received rivaroxaban for the first five postoperative days, switching to aspirin was not significantly different from continuing rivaroxaban for preventing symptomatic VTE [6]. These trials supported aspirin as an extended prophylactic agent after an anticoagulant lead-in, but did not directly establish the effectiveness of aspirin from the immediate postoperative period.

The evidence for immediate aspirin monotherapy has been less consistent. The large CRISTAL cluster-randomized crossover trial reported symptomatic VTE in 3.45% of patients assigned to aspirin and 1.82% of those assigned to enoxaparin, failing to demonstrate noninferiority of aspirin and showing superiority of enoxaparin for the trial’s composite outcome, which included symptomatic distal DVT [1]. In contrast, the recently published EPCAT III trial randomized 5429 patients to aspirin from the initial postoperative period or rivaroxaban for five days followed by aspirin. Symptomatic proximal DVT or PE occurred in 0.48% and 0.45% of patients, respectively, establishing noninferiority of immediate aspirin, with no clinically relevant difference in major or clinically relevant nonmajor bleeding [7].

Recent meta-analyses have generally suggested that aspirin has efficacy and safety comparable to direct oral anticoagulants or low-molecular-weight heparin; however, the certainty of this evidence is limited by substantial clinical and methodological heterogeneity across trials [8,9,10]. This systematic review and meta-analysis aimed to evaluate the efficacy and safety of aspirin-based thromboprophylaxis compared with anticoagulant regimens after total hip or knee arthroplasty, while accounting for differences in surgical procedure, aspirin initiation strategy, total daily aspirin dose, comparator class, and follow-up period.

## 2. Materials and Methods

### 2.1. Study Design and Registration

This systematic review and meta-analysis was conducted in accordance with the Cochrane Handbook for Systematic Reviews of Interventions [11] and reported following the Preferred Reporting Items for Systematic Reviews and Meta-Analyses (PRISMA) 2020 statement [12]. The study protocol was prospectively registered in the International Prospective Register of Systematic Reviews (PROSPERO; CRD420261465564) [13]. The completed PRISMA 2020 checklist is provided as Supplementary File S1.

### 2.2. Literature Search and Study Selection

A comprehensive literature search was performed in PubMed, Scopus, Web of Science (WoS), and the Cochrane Central Register of Controlled Trials (CENTRAL) from database inception to 25 July 2026. The search strategy combined terms related to total knee arthroplasty, total hip arthroplasty, and aspirin, including the keywords: (“total knee arthroplasty” OR TKA OR “total hip arthroplasty” OR THA) AND (“aspirin” OR “acetyl salicylic acid” OR ASA). No restrictions were applied during the initial search process.

The complete search strategies for all databases are provided in Supplementary Table S1.

All retrieved records were imported into EndNote 2025 [14] for duplicate removal and subsequently uploaded to the Rayyan web platform (Rayyan Systems Inc., Cambridge, MA, USA; https://www.rayyan.ai/, accessed on 1 July 2026) [15] for screening.

Two reviewers independently screened titles and abstracts for potential eligibility. Full-text articles of potentially relevant studies were then assessed against the predefined eligibility criteria. Disagreements were resolved through discussion and consensus. In addition, forward and backward citation searches were performed manually to identify any additional eligible studies.

### 2.3. Eligibility Criteria and Outcomes

We included RCTs that compared aspirin-based thromboprophylaxis with anticoagulant regimens after total hip or knee arthroplasty. We excluded observational studies, non-randomized trials, review articles, editorials, letters, conference abstracts lacking sufficient data for extraction, and non-English publications.

The primary outcomes were any DVT, symptomatic DVT, and symptomatic VTE. Secondary outcomes included PE, proximal DVT, distal DVT, any bleeding, major bleeding, minor bleeding, gastrointestinal (GI) bleeding, clinically relevant non-major bleeding (CRNMB), ecchymosis or bruising, blood transfusion, wound infection, wound hematoma, and mortality.

### 2.4. Data Extraction and Risk of Bias Assessment

Data were extracted independently by two authors using a standardized, pilot-tested electronic data extraction form. Extracted information included study-level characteristics (study design, blinding status, study time, centers, country, sample size, population, aspirin regimen, comparator class, comparator drug regimen, non-pharmacologic co-intervention, follow-up duration, VTE ascertainment, and study conclusion), patient-level characteristics (age, sex, BMI, surgery type, surgery class, laterality, osteoarthritis, hypertension, diabetes mellitus, previous VTE, cancer, current smoker, hemoglobin, and platelet count), risk-of-bias assessments, and all predefined efficacy and safety outcomes. Any discrepancies in data extraction were resolved through discussion and consensus among the investigators.

The methodological quality of included RCTs was assessed using the Cochrane Risk of Bias 2 (ROB 2) tool [16]. Five domains were evaluated: bias arising from the randomization process, deviations from intended interventions, missing outcome data, outcome measurement, and selection of the reported result. Each domain was rated as low risk of bias, some concerns, or high risk of bias. An overall low-risk judgment required all domains to be rated as low risk; an overall judgment of some concerns applied when at least one domain raised some concerns, and no domain was rated as high risk, unless concerns across multiple domains substantially lowered confidence in the result. An overall high-risk judgment was applied when at least one domain was rated as high risk or concerns across multiple domains substantially lowered confidence in the result.

### 2.5. Statistical Analysis

For our dichotomous outcomes, pooled risk ratios (RRs) with 95% CIs were calculated. The main risk-ratio meta-analyses were conducted using DerSimonian–Laird random-effects models. Statistical heterogeneity was assessed using Cochran’s Q test and quantified with the I^2^ statistic. Statistical heterogeneity was considered substantial when I^2^ exceeded 50% and/or Cochran’s Q test yielded a *p*-value < 0.05. When significant heterogeneity was identified, leave-one-out sensitivity analyses were performed to evaluate the influence of individual studies on the pooled effect estimate.

Two included trials were retained in the qualitative review and risk-of-bias summary but excluded from quantitative synthesis because they were judged to be at overall high risk of bias using RoB 2 [17,18]. Trials assessed as low risk of bias or as having some concerns were included in the quantitative analyses. The EPCAT III trial was excluded only from the overall pooled analysis because aspirin was administered in both treatment groups, making its comparison conceptually distinct from trials in which aspirin was used exclusively in the intervention arm. However, it was included in the aspirin-monotherapy analysis alongside studies comparing aspirin alone with LMWH, a direct oral anticoagulant (DOAC), sequential LMWH–DOAC therapy, or sequential DOAC–aspirin therapy [7].

Prespecified subgroup analyses were performed according to population characteristics, intervention characteristics, and follow-up duration when sufficient data were available. Specifically, studies were stratified according to surgery type (THA only, TKA only, or mixed THA/TKA), surgery class (primary arthroplasty only or mixed surgical classes), laterality (unilateral procedures only or mixed unilateral/bilateral procedures), timing of aspirin initiation (within 24 h postoperatively without an anticoagulant lead-in or after 24 h following an anticoagulant lead-in), intervention regimen (aspirin alone, LMWH followed by aspirin, or a DOAC followed by aspirin), total daily aspirin dose (<100 mg, 100 mg, or >100 mg), and follow-up duration (28, 42, or 90 days). An additional subgroup analysis was conducted among studies in which aspirin alone was administered in the intervention group, with studies stratified according to comparator regimen as LMWH alone, a DOAC alone, or a combined pharmacologic protocol. Galbraith plots were generated to explore potential sources of heterogeneity. For outcomes reported in at least 10 studies, we assessed the publication bias using the funnel plot methodology to visualize the association between effect size and standard error, and Egger’s regression test was performed to measure its statistical evidence. A *p*-value < 0.10 was considered indicative of potential small-study effects. Doi plots and LFK indices were explored for small-study effects when applicable and interpreted cautiously given the limited number of studies. All statistical analyses were done using Stata/MP version 19 for Windows (StataCorp LLC, College Station, TX, USA).

Additional post hoc sensitivity analyses were performed for the three primary outcomes, restricting inclusion to trials judged to be at overall low risk of bias according to RoB 2 and retaining the eligibility criteria of the main pooled comparisons. Risk ratios with 95% confidence intervals were pooled using DerSimonian–Laird random-effects models. Zhou 2023’s rivaroxaban and dalteparin groups were combined into a single anticoagulant comparator to avoid counting the aspirin group twice [19].

Sensitivity analyses excluding CRISTAL were assessed for symptomatic DVT, symptomatic VTE, and PE. The symptomatic DVT and symptomatic VTE results were obtained from the existing leave-one-out analyses. An additional PE analysis was reconstructed from the displayed event counts and denominators using a DerSimonian–Laird random-effects model. For this analysis, Chung’s two rivaroxaban arms were combined into a single comparator, and its published procedure denominators were retained without adjustment for within-patient correlation. Trials with zero events in both arms were excluded from risk-ratio pooling, and a continuity correction of 0.5 was applied to all four cells in trials with a zero-event arm.

The main risk-ratio analyses used aggregate event counts and did not preserve CRISTAL’s adjustment for its cluster-randomized crossover design. Additional post hoc sensitivity analyses therefore incorporated CRISTAL’s published cluster-adjusted risk differences for any DVT, symptomatic DVT, symptomatic VTE, and PE, with standard errors approximated from the reported 95% confidence intervals [1]. These estimates were combined with risk differences reconstructed for the other trials using generic inverse-variance random-effects models, restricted maximum likelihood estimation, and normal 95% confidence intervals. Zero-event handling and other methodological details are provided with Supplementary Figures S50–S53.

A post hoc exploratory analysis stratified trials by total pharmacological prophylaxis duration (≤14 versus >14 days), including any anticoagulant lead-in. Trials that could not be assigned to a single duration category were omitted from this analysis.

Estimates were reconstructed from displayed event counts using DerSimonian–Laird random-effects models; duration classification, exclusions, and detailed methods are provided in the notes accompanying Supplementary Figures S47–S49.

## 3. Results

### 3.1. Search Results

**Figure 1:**
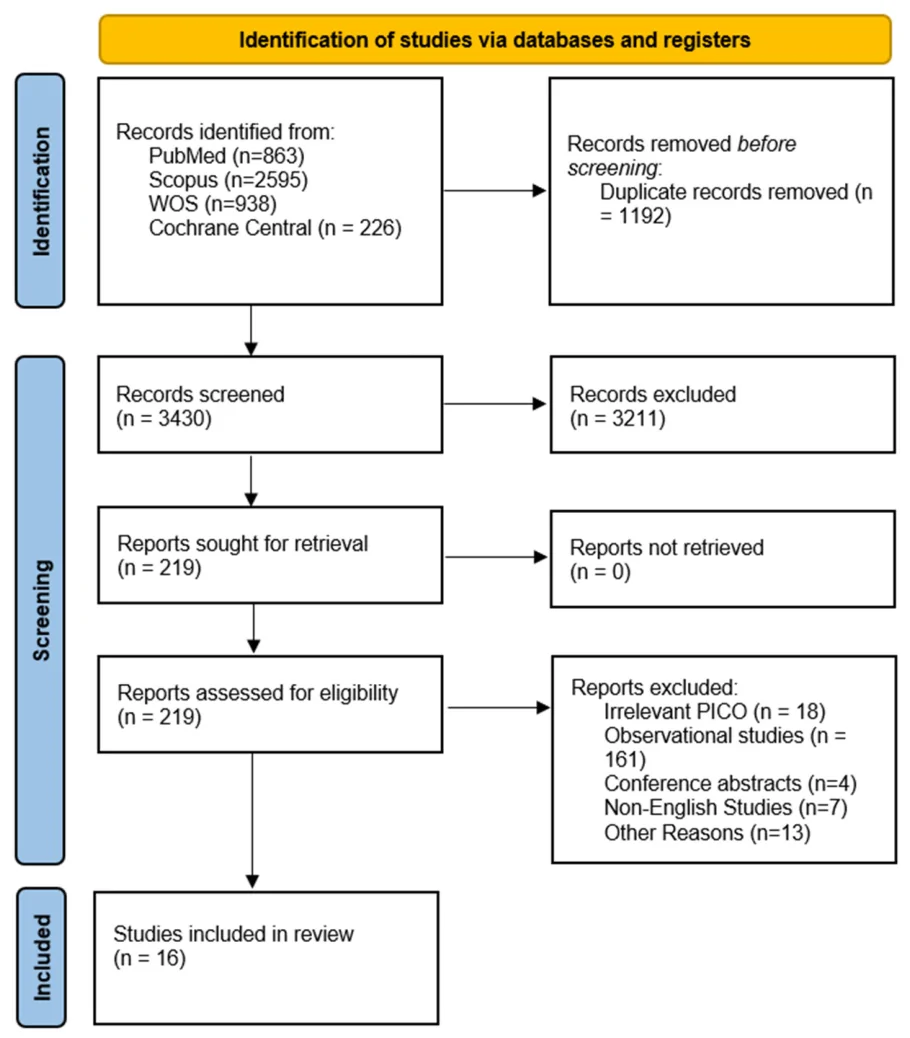
PRISMA flow diagram.

The literature search identified 4622 records. After removal of 1192 duplicates, 3430 records underwent title and abstract screening, of which 3211 were excluded. The full texts of the remaining articles were assessed for eligibility, resulting in the inclusion of 16 RCTs [1,5,6,7,17,18,19,20,21,22,23,24,25,26,27,28]. The study selection process is summarized in the PRISMA flow diagram (Figure 1).

### 3.2. Characteristics of Included Studies and Participants

The 16 included RCTs comprised 21,265 participants and were published between 2006 and 2026 across Australia, Brazil, Canada, China, India, Israel, Mexico, Pakistan, South Korea, and the United States. Four were multicenter trials [1,5,6,7], whereas 12 were conducted at a single center [17,18,19,20,21,22,23,24,25,26,27,28]. Six studies enrolled mixed THA/TKA populations [1,6,7,18,20,27], six included only patients undergoing TKA [19,23,24,25,26,28], and four included only patients undergoing THA [5,17,21,22]. Eleven trials were restricted to primary arthroplasty [1,17,18,19,21,23,24,25,26,27,28], four included mixed surgical classes [5,6,7,20], and one did not clearly report primary or revision status [22]. Aspirin was initiated within 24 h postoperatively without an anticoagulant lead-in in 10 studies [1,7,19,21,22,24,25,26,27,28], whereas six initiated aspirin after an anticoagulant lead-in [5,6,17,18,20,23]. Total daily aspirin doses ranged from 75 to 650 mg, treatment duration ranged from in-hospital prophylaxis to 42 days, and follow-up ranged from 28 to 90 days. Comparator regimens included LMWH, direct oral anticoagulants, continued anticoagulant therapy, and sequential anticoagulant protocols. The reported mean or median participant age ranged from 48.3 to 71.2 years. Baseline characteristics were generally comparable between treatment groups. Detailed study and participant characteristics are presented in Table 1 and Table 2.

### 3.3. Risk of Bias Assessment

**Figure 2:**
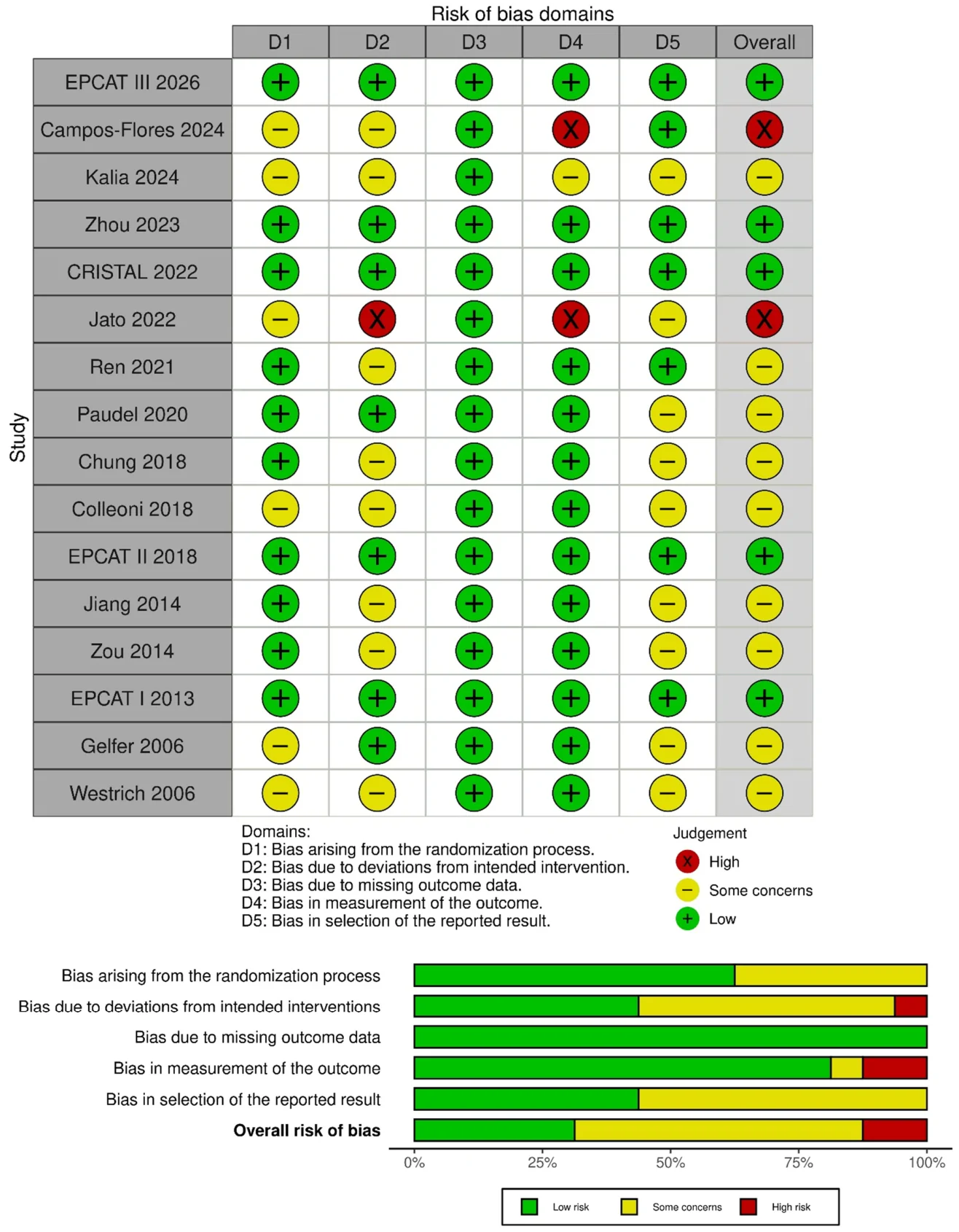
ROB 2 assessment of included trials. Study references: [1,5,6,7,17,18,19,20,21,22,23,24,25,26,27,28].

Overall, five trials were judged to be at low risk of bias [1,5,6,7,19], nine raised some concerns [20,21,22,23,24,25,26,27,28], and two were judged to be at high risk of bias [17,18]. Both high-risk trials had a high risk of bias in outcome measurement [17,18], while Jato et al. [17] also had a high risk of bias due to deviations from intended interventions. Detailed RoB 2 assessments are presented in Figure 2.

### 3.4. Primary Outcomes

#### 3.4.1. Any Deep Vein Thrombosis

**Figure 3:**
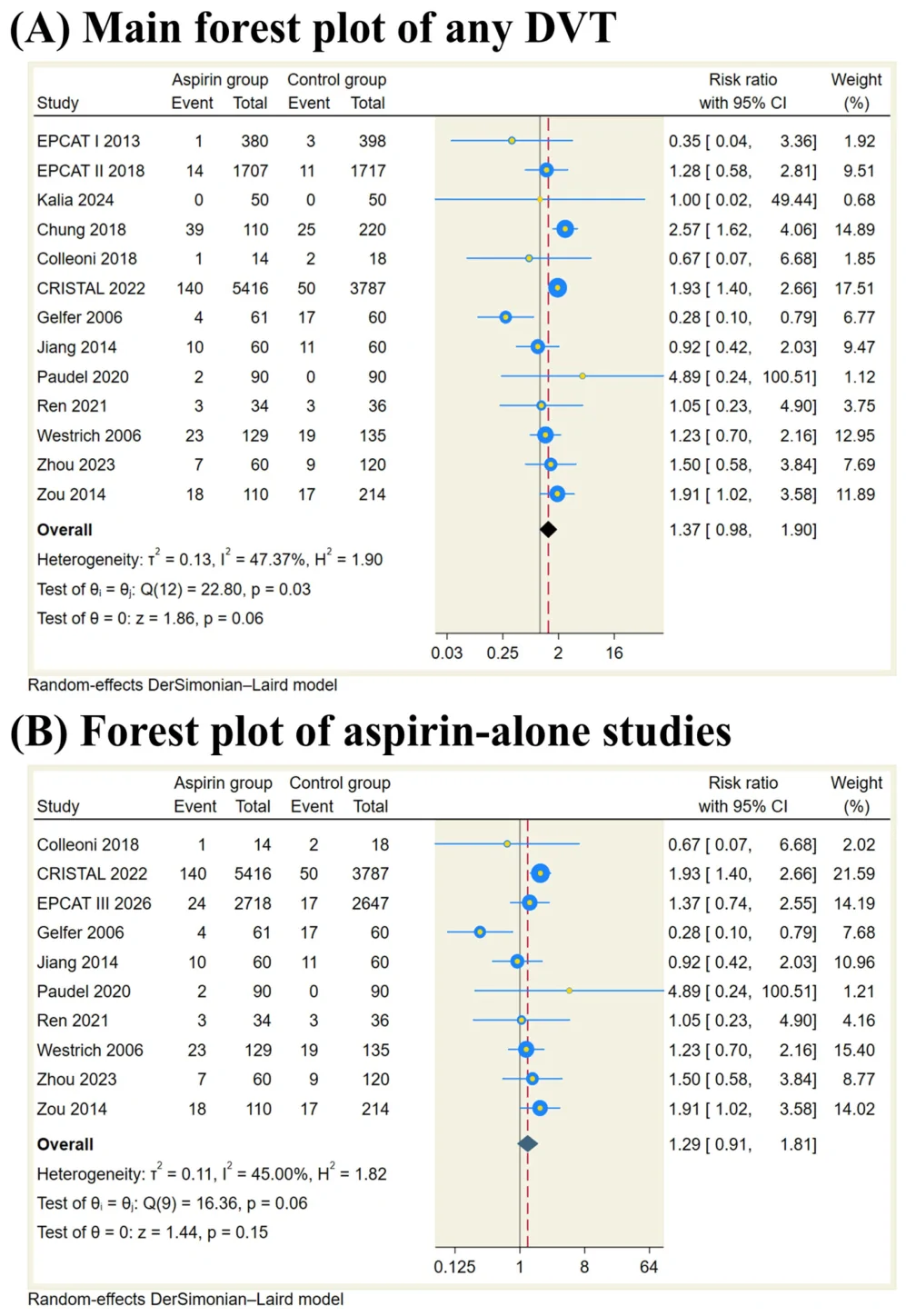
Any deep vein thrombosis. (**A**) Main pooled analysis comparing aspirin-based thromboprophylaxis with anticoagulant regimens. (**B**) Pooled analysis restricted to aspirin-alone regimens. Yellow points indicate study RRs (circle size reflects weight), horizontal lines show 95% CIs, and diamonds indicate pooled RRs. The grey solid line marks RR = 1; the red dashed line marks the pooled RR CI, confidence interval; DVT, deep vein thrombosis; RR, risk ratio. Study references: [1,5,6,7,19,20,21,22,23,24,25,26,27,28].

Thirteen studies reported any DVT. The pooled analysis suggested a higher risk of any DVT with aspirin-based thromboprophylaxis than with anticoagulant regimens, although the difference did not reach statistical significance (RR 1.37, 95% CI 0.98 to 1.90; *p* = 0.06), with moderate heterogeneity (I^2^ = 47.37%; Figure 3A). The analysis restricted to aspirin-alone regimens produced a similar non-significant estimate (RR 1.29, 95% CI 0.91 to 1.81; *p* = 0.15; I^2^ = 45.00%; Figure 3B).

Subgroup analyses showed no significant differences according to aspirin initiation timing, intervention regimen, follow-up duration, surgery class, surgery type, or total daily aspirin dose (all *p* for interaction > 0.05). However, a significant interaction was observed according to laterality (*p* for interaction < 0.001): aspirin was associated with a higher risk of any DVT in studies including unilateral and bilateral procedures (RR 2.13, 95% CI 1.64 to 2.77), whereas no significant association was observed in studies restricted to unilateral procedures (RR 1.09, 95% CI 0.76 to 1.55). All subgroup analyses of any DVT are presented in Supplementary Figures S1–S7. Leave-one-out analyses indicated that the overall finding was not fully robust, as the association became statistically significant after the omission of EPCAT I 2013, Gelfer 2006, or Jiang 2014 (Supplementary Figure S8) [5,25,27]. The Galbraith plot identified Gelfer 2006 as a potential source of heterogeneity (Supplementary Figure S9) [27]. Visual inspection of the funnel plot and Egger’s regression test suggested possible small-study effects (*p* = 0.0972; Supplementary Figure S10).

#### 3.4.2. Symptomatic Deep Vein Thrombosis

**Figure 4:**
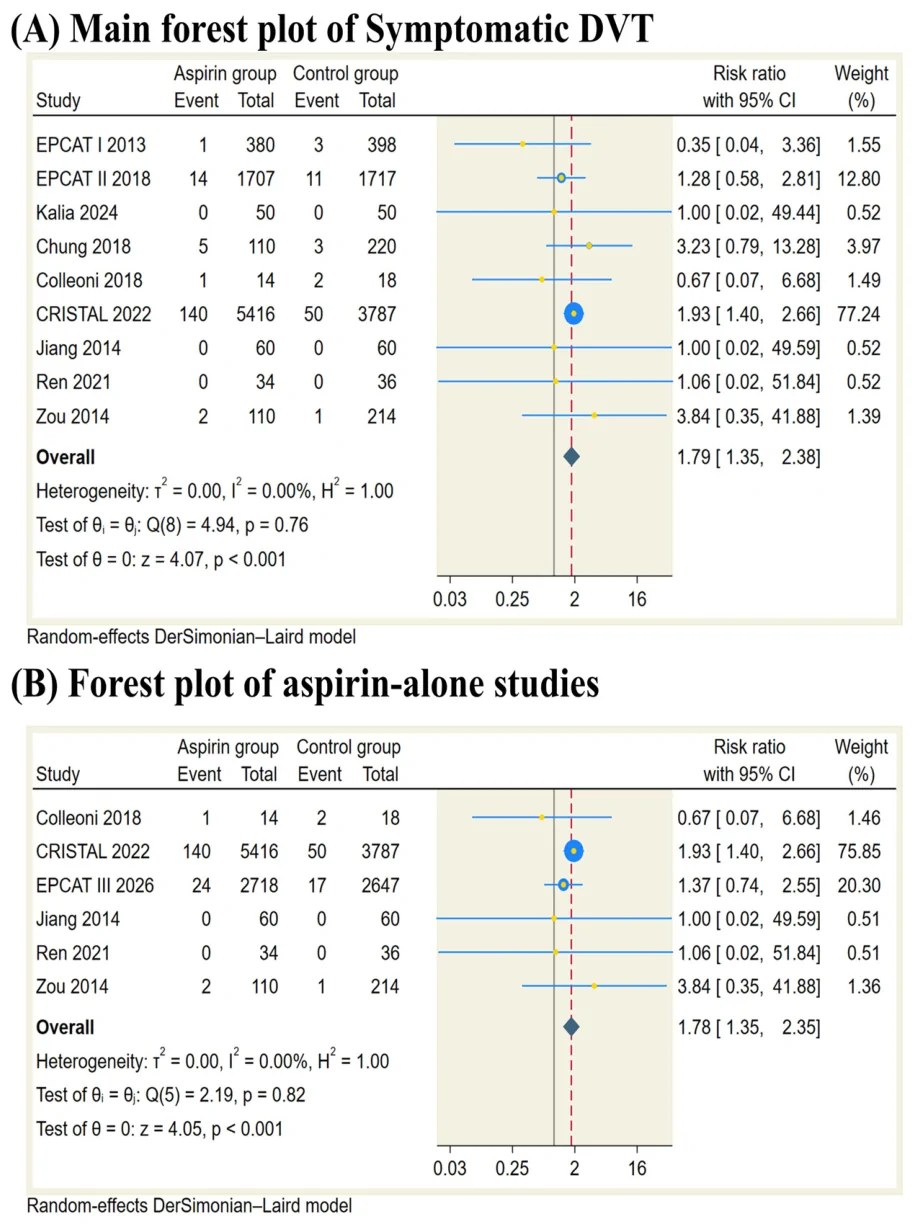
Symptomatic deep vein thrombosis. (**A**) Main pooled analysis comparing aspirin-based thromboprophylaxis with anticoagulant regimens. (**B**) Pooled analysis restricted to aspirin-alone regimens. Yellow points indicate study RRs (circle size reflects weight), horizontal lines show 95% CIs, and diamonds indicate pooled RRs. The grey solid line marks RR = 1; the red dashed line marks the pooled RR. CI, confidence interval; DVT, deep vein thrombosis; RR, risk ratio. Study references: [1,5,6,7,20,21,23,24,25,26].

Nine studies reported symptomatic DVT. Aspirin-based thromboprophylaxis was associated with a significantly higher risk of symptomatic DVT than anticoagulant regimens (RR 1.79, 95% CI 1.35 to 2.38; *p* < 0.001), with no observed statistical heterogeneity (I^2^ = 0%; Figure 4A). A comparable association was observed in the aspirin-alone analysis (RR 1.78, 95% CI 1.35 to 2.35; *p* < 0.001; I^2^ = 0%; Figure 4B).

The direction of effect was generally consistent across subgroups defined by aspirin initiation timing, intervention regimen, laterality, surgery class, surgery type, total daily aspirin dose, and follow-up duration, with no significant subgroup interactions (all *p* for interaction > 0.05; Supplementary Figures S11–S17).

In the leave-one-out sensitivity analysis, exclusion of CRISTAL 2022 attenuated the pooled estimate for symptomatic DVT to RR 1.39 (95% CI 0.77–2.50; *p* = 0.277), and the association was no longer statistically significant [1]. Exclusion of any other individual study did not remove statistical significance (Supplementary Figure S18).

The Galbraith plot did not identify a clear statistical outlier (Supplementary Figure S19), whereas the Doi plot suggested major asymmetry (LFK index = −4.02; Supplementary Figure S20).

#### 3.4.3. Symptomatic Venous Thromboembolism

**Figure 5:**
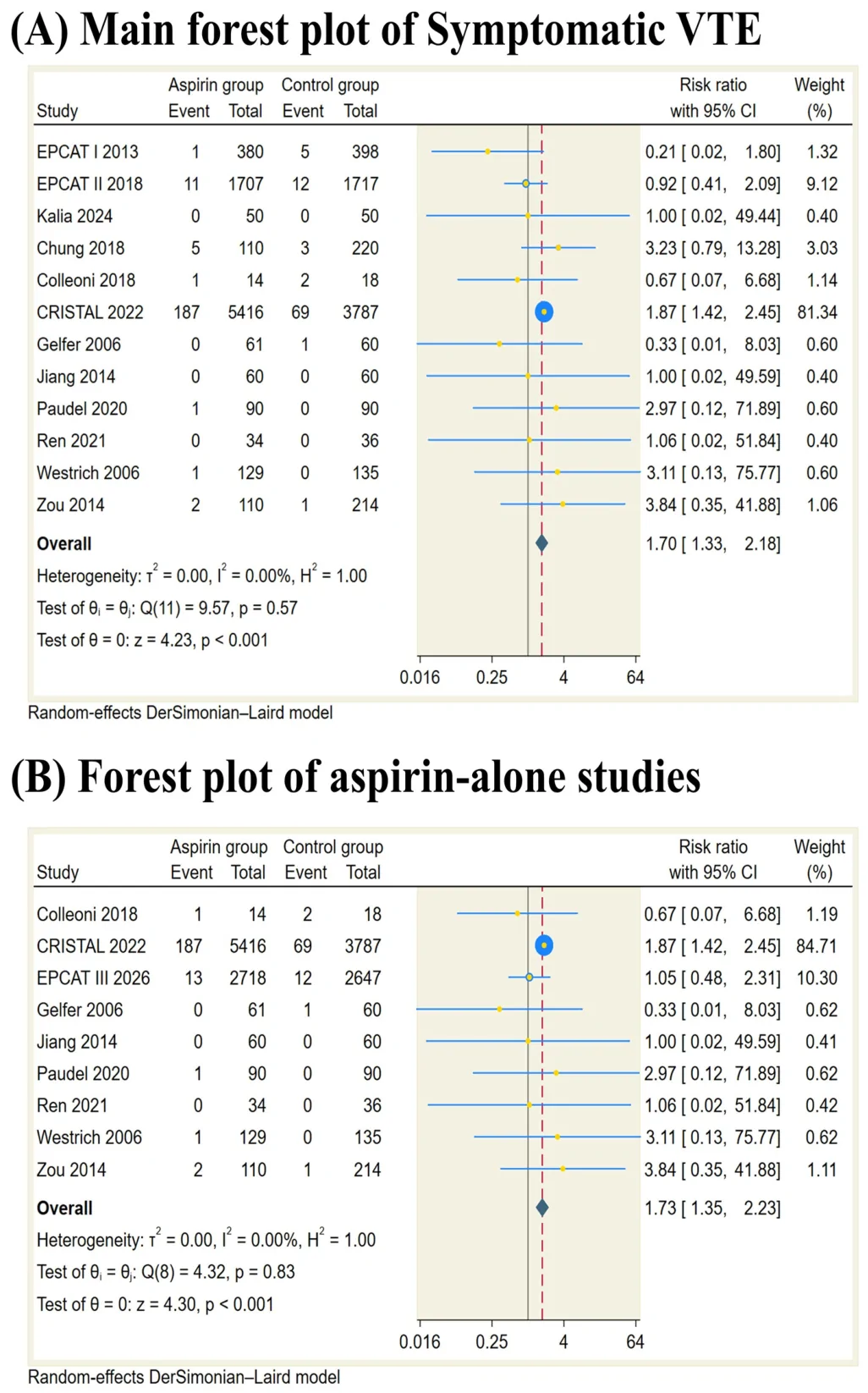
Symptomatic venous thromboembolism. (**A**) Main pooled analysis comparing aspirin-based thromboprophylaxis with anticoagulant regimens. (**B**) Pooled analysis restricted to aspirin-alone regimens. Yellow points indicate study RRs (circle size reflects weight), horizontal lines show 95% CIs, and diamonds indicate pooled RRs. The grey solid line marks RR = 1; the red dashed line marks the pooled RR. CI, confidence interval; RR, risk ratio; VTE, venous thromboembolism. Study references: [1,5,6,7,20,21,22,23,24,25,26,27,28].

Twelve studies reported symptomatic venous thromboembolism (VTE). Aspirin-based thromboprophylaxis was associated with a significantly higher risk of symptomatic VTE than anticoagulant regimens (RR 1.70, 95% CI 1.33 to 2.18; *p* < 0.001), with no observed heterogeneity (I^2^ = 0%; Figure 5A). The analysis restricted to aspirin-alone regimens yielded a similar result (RR 1.73, 95% CI 1.35 to 2.23; *p* < 0.001; I^2^ = 0%; Figure 5B).

No significant subgroup interactions were observed according to aspirin initiation timing, intervention regimen, follow-up duration, or surgery type. Significant interactions were identified according to laterality and surgery class (both *p* for interaction = 0.03). Aspirin was associated with a higher risk of symptomatic VTE in studies including both unilateral and bilateral procedures (RR 1.91, 95% CI 1.46–2.49), whereas no significant association was observed in studies restricted to unilateral procedures (RR 0.89, 95% CI 0.47–1.68). Similarly, an increased risk was observed in primary-only arthroplasty studies (RR 1.87, 95% CI 1.44–2.42), but not in studies enrolling mixed surgical classes (RR 0.77, 95% CI 0.37–1.63). The interaction according to total daily aspirin dose did not reach statistical significance (*p* for interaction = 0.08). These subgroup analyses are presented in Supplementary Figures S21–S27. In the leave-one-out sensitivity analysis, exclusion of CRISTAL 2022 attenuated the pooled estimate for symptomatic VTE to RR 1.14 (95% CI 0.64–2.01; *p* = 0.655), and the association was no longer statistically significant [1]. Exclusion of any other individual study did not remove statistical significance (Supplementary Figure S28). The Galbraith plot did not identify a clear statistical outlier (Supplementary Figure S29). Visual inspection of the funnel plot and Egger’s regression test provided no evidence of small-study effects (*p* = 0.2698; Supplementary Figure S30).

In sensitivity analyses restricted to trials at overall low risk of bias, four trials contributed to the any-DVT analysis: EPCAT I, EPCAT II, CRISTAL, and Zhou 2023 [19]. Aspirin-based prophylaxis was associated with a higher risk of any DVT (RR 1.75, 95% CI 1.30–2.36; *p* < 0.001; I^2^ = 2.9%). Three trials—EPCAT I, EPCAT II, and CRISTAL—contributed to the symptomatic DVT and symptomatic VTE analyses. Neither association reached statistical significance: symptomatic DVT yielded an RR of 1.58 (95% CI 0.93–2.68; *p* = 0.089; I^2^ = 33.5%), and symptomatic VTE yielded an RR of 1.10 (95% CI 0.46–2.64; I^2^ = 68.9%). (Supplementary Figures S43–S45)

In the additional sensitivity analysis excluding CRISTAL, six trials contributed estimable risk ratios. The pooled estimate for PE was RR 1.39 (95% CI 0.56–3.46; *p* = 0.482; I^2^ = 40.3%; Supplementary Figure S46), indicating no statistically significant difference between aspirin-based prophylaxis and anticoagulant regimens. Five trials reporting no PE events in either arm did not contribute to the pooled risk ratio. Both PE analyses combined Chung’s comparator arms; however, the analysis excluding CRISTAL also excluded double-zero trials, which were retained in the main analysis.

No statistically significant duration interaction was detected for any DVT (*p* = 0.366), symptomatic DVT (*p* = 0.131), or symptomatic VTE (*p* = 0.346; Supplementary Figures S47–S49). Symptomatic estimates were imprecise, and only EPCAT I contributed an estimable symptomatic DVT result to the >14-day subgroup.

### 3.5. Secondary Outcomes

**Figure 6:**
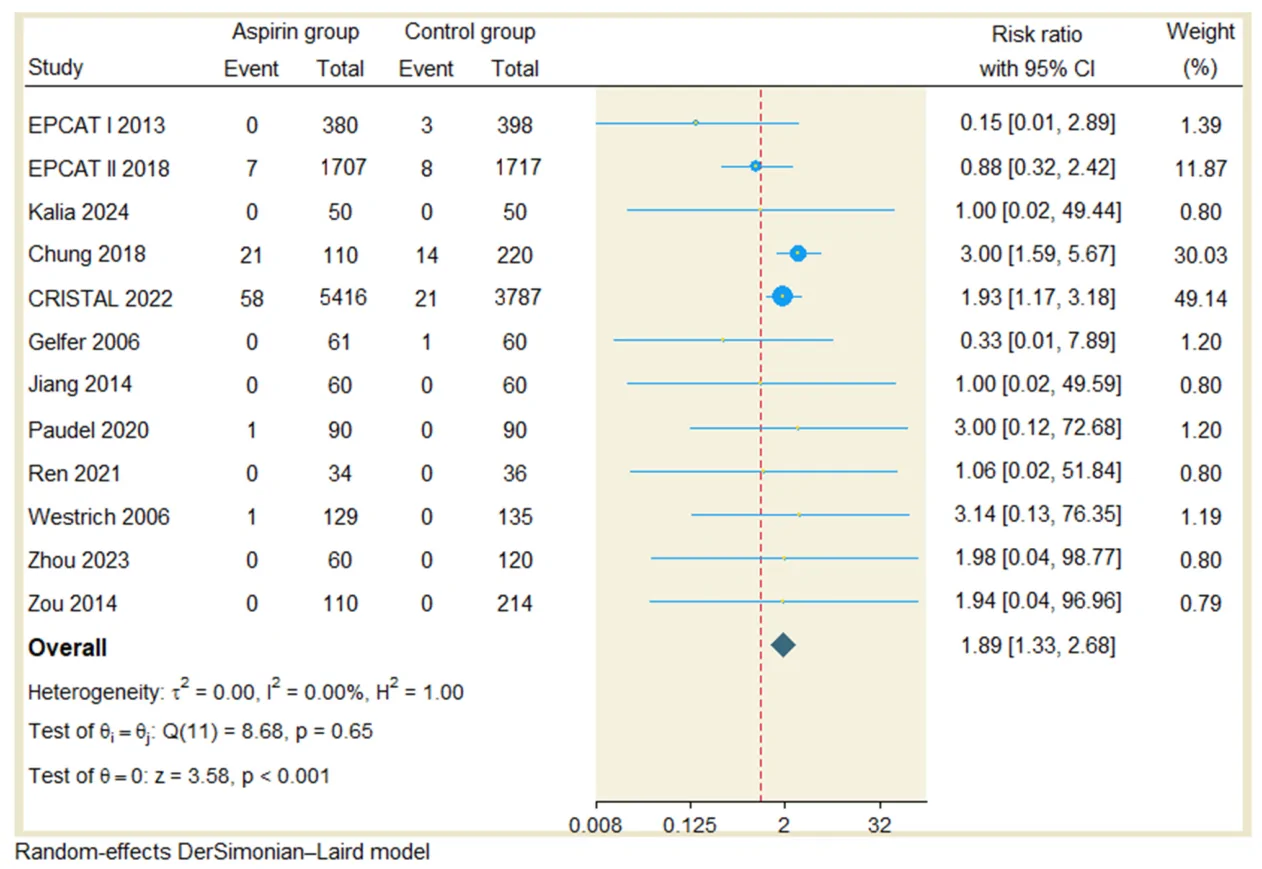
Pooled analysis of pulmonary embolism comparing aspirin-based thromboprophylaxis with anticoagulant regimens. Yellow points indicate study RRs (circle size reflects weight), horizontal lines show 95% CIs, and diamonds indicate pooled RRs. The grey solid line marks RR = 1; the red dashed line marks the pooled RR. CI, confidence interval; RR, risk ratio. Study references: [1,5,6,19,20,21,22,23,25,26,27,28].

Pulmonary embolism was reported in 12 trials. After combining Chung’s two rivaroxaban groups into a single comparator, aspirin-based thromboprophylaxis was associated with a significantly higher risk of pulmonary embolism than anticoagulant regimens (RR 1.89, 95% CI 1.33–2.68; *p* < 0.001; I^2^ = 0%; Figure 6). No significant difference was observed for proximal DVT (RR 1.08, 95% CI 0.63–1.85; *p* = 0.79; I^2^ = 0%; Supplementary Figure S31), whereas Aspirin was associated with a higher risk of distal DVT than anticoagulant regimens (RR 1.47, 95% CI 1.06–2.06; *p* = 0.02; I^2^ = 34.60%; Supplementary Figure S32).

No statistically significant between-group differences were detected in major bleeding (RR 0.81, 95% CI 0.46–1.42; *p* = 0.46; I^2^ = 1.59%), clinically relevant non-major bleeding (RR 1.02, 95% CI 0.51–2.03; *p* = 0.95; I^2^ = 0%), any bleeding (RR 0.73, 95% CI 0.42–1.26; *p* = 0.26; I^2^ = 25.44%), or gastrointestinal bleeding (RR 0.84, 95% CI 0.18–3.80; *p* = 0.82; I^2^ = 0%). Aspirin was associated with lower risks of minor bleeding (RR 0.47, 95% CI 0.31–0.71; *p* < 0.001; I^2^ = 0%) and ecchymosis or bruising (RR 0.60, 95% CI 0.39–0.93; *p* = 0.02; I^2^ = 39.34%).

No statistically significant differences were detected in wound infection (RR 1.24, 95% CI 0.59–2.57; *p* = 0.57; I^2^ = 0%), wound hematoma (RR 0.38, 95% CI 0.13–1.16; *p* = 0.09; I^2^ = 0%), blood transfusion (RR 0.81, 95% CI 0.53–1.23; *p* = 0.33; I^2^ = 22.35%), or mortality (RR 1.06, 95% CI 0.36–3.14; *p* = 0.92; I^2^ = 0%). The corresponding forest plots are presented in Supplementary Figures S33–S42.

### 3.6. Sensitivity Analyses Incorporating CRISTAL’s Cluster Adjustment

In additional post hoc risk-difference analyses incorporating CRISTAL’s published cluster-adjusted estimates, the pooled absolute differences were 1.87 percentage points for any DVT (95% CI −2.48 to 6.21), 0.51 for symptomatic DVT (95% CI −0.32 to 1.34), 0.41 for symptomatic VTE (95% CI −0.42 to 1.24), and 0.08 for PE (95% CI −0.29 to 0.44; Supplementary Figures S50–S53, respectively). Positive values indicate greater risk with aspirin. All confidence intervals included zero. Heterogeneity was substantial for the any-DVT risk-difference analysis (I^2^ = 97.8%).

## 4. Discussion

In this updated meta-analysis of 16 randomized controlled trials involving 21,265 participants, aspirin-based thromboprophylaxis was associated with higher risks of symptomatic DVT, symptomatic VTE, PE, and distal DVT than anticoagulant regimens. The difference in any DVT did not reach statistical significance, and no significant difference was observed for proximal DVT. Aspirin reduced minor bleeding and ecchymosis or bruising, but it did not significantly reduce major bleeding, clinically relevant non-major bleeding, wound complications, blood transfusion, or mortality. However, the thromboembolic associations were sensitive to trial inclusion and analytical approach, limiting confidence in a consistent difference between prophylactic strategies.

These results differ from several earlier meta-analyses that reported broadly comparable efficacy between aspirin and anticoagulants [8,9,29]. Salman et al. reported a higher risk of PE with aspirin than with LMWH, whereas other analyses and an umbrella review found similar overall VTE outcomes with fewer bleeding complications [10,30,31]. The present analysis may provide a different estimate because it separated symptomatic from screening-detected events, analyzed proximal and distal DVT independently, excluded trials judged to be at overall high risk of bias from quantitative synthesis, and incorporated large contemporary trials. These distinctions are important because composite VTE outcomes can conceal clinically relevant differences between symptomatic PE, proximal DVT, distal DVT, and asymptomatic thrombosis detected only through routine imaging.

The contrasting designs of CRISTAL and the EPCAT trials help explain the apparent inconsistency in the randomized evidence. CRISTAL compared aspirin with enoxaparin throughout prophylaxis and found more symptomatic VTE with aspirin [1]. EPCAT I and EPCAT II instead evaluated aspirin after an initial course of dalteparin or rivaroxaban and therefore primarily support aspirin as extended prophylaxis after early postoperative anticoagulation [5,6]. EPCAT III compared immediate aspirin with five days of rivaroxaban followed by aspirin, after which both groups received aspirin, and used symptomatic proximal DVT or PE as its primary outcome [7]. Its noninferiority finding therefore does not establish equivalence between aspirin and a full course of anticoagulation in all arthroplasty populations. Taken together, the evidence suggests that treatment timing, comparator duration, patient selection, and outcome definition materially influence the observed effect.

CRISTAL’s cluster-randomized crossover design warrants specific consideration because treatment allocation occurred at the hospital level. The investigators used hospital-level cluster-summary methods, with harmonic-mean weighting to accommodate unequal cluster-period sizes. Following early cessation, crossover estimates were combined with parallel cluster estimates from hospitals that had not crossed over [1]. These methods explicitly addressed clustering and incomplete crossover, supporting the methodological credibility of the trial’s analysis. Nevertheless, only 16 of 31 hospitals crossed over, limiting the intended within-hospital comparisons [1]. Furthermore, unblinded treatment allocation and variable enrolment rates leave potential for selective recruitment and differential diagnostic testing that statistical adjustment for clustering cannot eliminate [1]. Supplementary analyses examining hospital characteristics and missing outcomes supported the consistency of the symptomatic VTE finding, although they cannot exclude all residual bias [1]. CRISTAL should therefore be regarded as influential randomized evidence with identifiable design limitations, rather than considered unreliable solely because of cluster randomization.

Although CRISTAL’s original analysis accounted for its cluster-randomized crossover design, this adjustment was not preserved in our main analyses based on aggregate event counts. Additional analyses incorporating its published cluster-adjusted risk differences yielded confidence intervals crossing zero for all three primary outcomes and PE. These analyses also changed the effect measure and pooling method; consequently, differences from the main risk-ratio findings cannot be attributed solely to cluster adjustment. Risk-difference pooling is sensitive to sparse events and variation in baseline risk. The resulting uncertainty does not establish equivalence between treatments.

Most subgroup analyses did not identify significant differences according to aspirin initiation timing, intervention regimen, surgery type, aspirin dose, or follow-up duration. The significant interactions by procedure laterality and surgery class should be considered exploratory and hypothesis-generating. These comparisons were based on aggregate study-level characteristics, involved multiple subgroup tests, and were strongly influenced by the distribution and weight of CRISTAL. They therefore do not establish that the relative effectiveness of aspirin differs between individual patients undergoing unilateral versus bilateral procedures or primary versus revision arthroplasty. The leave-one-out analyses also demonstrated substantial dependence on this trial: after its exclusion, the pooled estimates were RR 1.39 (95% CI 0.77–2.50) for symptomatic DVT and RR 1.14 (95% CI 0.64–2.01) for symptomatic VTE. Although both point estimates remained above 1, their confidence intervals included both lower and higher risks with aspirin. Thus, the remaining trials did not independently demonstrate a statistically significant difference, and confidence in the consistency and generalisability of the overall findings is reduced. These results do not establish equivalence between aspirin and anticoagulant regimens.

### 4.1. Clinical Implications

Current guidelines reflect the continuing uncertainty surrounding aspirin. The American Society of Hematology conditionally recommends either aspirin or an anticoagulant after total hip or knee arthroplasty, but explicitly rates the certainty of evidence as very low [2]. NICE distinguishes between procedures, recommending LMWH followed by aspirin as one option after elective hip replacement while allowing aspirin monotherapy after knee replacement [3]. In contrast, the 2022 International Consensus Meeting strongly endorsed low-dose aspirin for many patients undergoing joint arthroplasty [4]. The present findings suggest that these recommendations should be applied with attention to the exact prophylactic strategy and the clinical outcome being prioritized.

Aspirin remains attractive because of its low cost, oral administration, availability, and lack of laboratory monitoring. It may be a reasonable component of prophylaxis in carefully selected patients, particularly as extended therapy after a short anticoagulant lead-in or when bleeding risk, treatment burden, cost, or adherence are major concerns. However, the current evidence does not support assuming universal equivalence between immediate aspirin monotherapy and anticoagulant regimens. Although the main risk-ratio analyses favoured anticoagulant regimens for several thromboembolic outcomes, these associations were not consistently supported across sensitivity analyses, limiting confidence in a general treatment advantage. Treatment selection should therefore incorporate the patient’s baseline thromboembolic and bleeding risks, the planned procedure, mobility, comorbidities, prior VTE history, and the feasibility of mechanical prophylaxis and early mobilization. Shared decision-making should communicate the observed reduction in minor bleeding with aspirin alongside the uncertainty regarding comparative thromboembolic protection.

Aspirin dose is another potential source of clinical heterogeneity. Total daily doses ranged from 75 to 650 mg across the included trials. Although the dose subgroup analyses did not identify statistically significant interactions, this should not be interpreted as evidence that different doses provide equivalent efficacy or safety. These comparisons were conducted between trials rather than through direct randomized comparisons of aspirin doses, and dose differences coincided with variation in surgical populations, comparator regimens, prophylaxis duration, and mechanical co-interventions. Consequently, this review cannot establish an optimal aspirin dose or a dose–response relationship. Direct randomized comparisons using standardized prophylaxis protocols and outcome definitions are needed.

### 4.2. Strengths and Limitations

This study has several strengths. It provides an updated synthesis of randomized evidence through July 2026 and incorporates EPCAT III, the largest individually randomized trial specifically addressing immediate aspirin initiation. Restriction to randomized trials reduced confounding inherent in observational comparisons. Clinically distinct outcomes were analyzed separately, including any DVT, symptomatic DVT, symptomatic VTE, PE, proximal DVT, distal DVT, and multiple bleeding outcomes. The additional analysis restricted to aspirin-alone intervention regimens reduced conceptual heterogeneity arising from sequential prophylaxis. Prespecified subgroup, leave-one-out, Galbraith, funnel-plot, Egger, and Doi-plot analyses were used to explore consistency, influence, heterogeneity, and possible small-study effects.

Several limitations should be considered. First, the pooled symptomatic estimates were heavily influenced by CRISTAL, and their statistical significance was not maintained when this trial was excluded. Although dominance by a large, methodologically robust trial is not inherently problematic, it limits the extent to which the pooled estimates represent an average effect across diverse settings. Second, substantial clinical heterogeneity remained across trials in aspirin dose, initiation time, duration, comparator regimen, use of mechanical prophylaxis, surgical procedure, laterality, and inclusion of primary or revision arthroplasty. The any-DVT outcome also combined symptomatic and screening-detected events assessed using different diagnostic strategies.

Restriction to trials at low risk of bias produced different findings across outcomes. The association between aspirin and increased any DVT remained statistically significant, whereas the symptomatic DVT and symptomatic VTE estimates had wider confidence intervals and did not reach statistical significance. These findings indicate that the main symptomatic associations were not consistently maintained after restriction to the low-risk subset. However, the wide confidence intervals do not establish equivalence between treatments and remain compatible with clinically important differences. Interpretation also requires consideration of the smaller number of included trials and differences in prophylactic strategies and outcome definitions.

The additional PE analysis excluding CRISTAL yielded a confidence interval crossing the null. However, this analysis also excluded double-zero trials, which were retained in the main PE analysis. The difference between these estimates therefore cannot be attributed solely to removal of CRISTAL. The wide confidence interval in the sensitivity analysis indicates substantial uncertainty and does not establish equivalence between aspirin and anticoagulant regimens.

Third, several outcomes were rare, producing zero-event cells and wide confidence intervals in individual studies. Safety outcomes were inconsistently defined and incompletely reported, limiting power to detect clinically important differences. Fourth, subgroup analyses were based on aggregate study-level data and were susceptible to ecological bias and confounding. Given the multiple comparisons and the influence of CRISTAL, the significant interactions by laterality and surgery class should be treated as exploratory findings requiring independent confirmation before informing clinical decisions. Fifth, evidence of possible small-study effects was observed for any DVT, and the Doi plot demonstrated major asymmetry for symptomatic DVT. These findings should be interpreted cautiously because statistical tests for asymmetry are unstable when few studies are available, but they reduce certainty in the precision of the pooled estimates.

### 4.3. Future Directions

Future trials should directly compare immediate aspirin monotherapy with full-course LMWH or DOAC prophylaxis using adequately powered, individually randomized, multicenter designs. Standardized outcome definitions should distinguish symptomatic proximal DVT, symptomatic distal DVT, PE, screening-detected thrombosis, major bleeding, clinically relevant non-major bleeding, and wound complications, with blinded independent adjudication. Trials should prospectively stratify patients by validated VTE and bleeding risk, procedure type, laterality, primary versus revision surgery, and mobility, and should evaluate absolute rather than only relative treatment effects. Individual-patient-data meta-analysis could clarify whether specific subgroups derive comparable protection from aspirin and whether a short anticoagulant lead-in meaningfully alters treatment effect. Adherence, patient preferences, cost-effectiveness, and quality-of-life outcomes should also be incorporated to define the net clinical value of each prophylactic strategy.

## 5. Conclusions

In this updated meta-analysis of randomized trials, aspirin-based thromboprophylaxis was associated with higher risks of symptomatic DVT, symptomatic VTE, PE, and distal DVT than anticoagulant regimens. No statistically significant differences were detected in major bleeding or clinically relevant non-major bleeding. However, the symptomatic DVT and symptomatic VTE associations were strongly influenced by CRISTAL and were no longer statistically significant after its exclusion.

Additional risk-difference sensitivity analyses incorporating CRISTAL’s published cluster-adjusted estimates did not identify statistically significant differences for any DVT, symptomatic DVT, symptomatic VTE, or PE. These findings limit the robustness of the main thromboembolic associations but do not establish equivalence between treatments.

Sensitivity analyses restricted to trials at low risk of bias identified a significantly higher risk of any DVT with aspirin, whereas the symptomatic DVT and symptomatic VTE associations did not reach statistical significance. The wide confidence intervals do not establish equivalence between treatments and remain compatible with clinically important differences.

Aspirin reduced minor bleeding and ecchymosis or bruising, but the balance between these benefits and thromboembolic risk remains uncertain. The findings should be interpreted in the context of CRISTAL’s influence, sensitivity to trial inclusion, and variation in prophylactic strategies and outcome definitions. Further adequately powered randomized trials are needed to clarify the relative efficacy and safety of immediate aspirin monotherapy compared with full-course anticoagulant prophylaxis.

## Figures and Tables

**Table 1:** Summary of included studies.

Study ID	Study Design	Blinding Status	Study Time	Centers	Country	Sample Size	Population				Aspirin regimen				Comparator Class	Comparator Drug Regimen	Non-Pharmacologic Co-Intervention	Follow-Up (Days)	VTE Ascertainment	Conclusions
							Surgery Type	Surgery Class	Laterality	Indication(s) for Surgery	Aspirin Initiation	Anticoagulant Lead-In	Aspirin Duration (Days)	Total Daily Aspirin Dose (mg)						
EPCAT III 2026 [7]	RCT	Double-blind	February 2021 to January 2026	Multicenter	Canada	5429	Mixed THA/TKA	Mixed primary/revision; mostly primary	Unilateral	Elective arthroplasty indications	Within 24 h postoperatively (No lead-in period)	No lead-in period	14 d (TKA); 35 d (THA)	81	Rivaroxaban (DOAC) then Aspirin	Rivaroxaban 10 mg daily for the first 5 postoperative days, followed by aspirin 81 mg daily for the remaining 9 d after TKA or 30 d after THA	Compression stockings permitted at surgeon discretion	90	Symptom-driven; no routine screening	Aspirin from the initial postoperative period was noninferior to 5 d rivaroxaban followed by aspirin for proximal DVT or PE, with similar clinically relevant bleeding.
Campos-Flores 2024 [18]	RCT	Open-label	April 2022 to October 2022	Single-center	Mexico	60	Mixed THA/TKA	Primary only	NA	Osteoarthritis	Postoperative day 4 (Anticoagulant lead-in followed by aspirin)	Enoxaparin (LMWH)	32 d	100	Enoxaparin (LMWH)	Enoxaparin 40 mg SC daily for 5 wk (20 mg in low weight or severe renal impairment)	Pneumatic compression stockings for at least 21 d and early physical therapy in both groups	90	Routine Doppler screening plus symptom reporting	No symptomatic or screening-detected VTE occurred in either group; aspirin after a short enoxaparin lead-in was reported as noninferior to continued enoxaparin.
Kalia 2024 [20]	RCT	NA	July 2022 to December 2023	Single-center	India	100	Mixed THA/TKA	Mixed primary/revision; distribution not reported	Unilateral	Elective arthroplasty indications	Postoperative day 6 (Anticoagulant lead-in followed by aspirin)	Rivaroxaban (DOAC)	9 d (TKA); 30 d (THA)	75	Rivaroxaban (DOAC) continued	Rivaroxaban 10 mg orally daily continued for the same extended period	No device intervention reported	90	Symptom-driven; no screening of asymptomatic patients at 90 d	The authors reported aspirin after 5 d rivaroxaban as similarly effective and safe to continued rivaroxaban.
Zhou 2023 [19]	RCT	Authors report patients, assessors, and data collectors were blinded	October 2020 to December 2021	Single-center	China	180	TKA only	Primary only	Unilateral	Osteoarthritis	Within 24 h postoperatively (No lead-in period)	No lead-in period	30 d	100	Dalteparin (LMWH)	Rivaroxaban 10 mg orally daily or dalteparin 2500 IU SC daily, each beginning 12 h postoperatively for 30 d	Routine rehabilitation, ankle pumps, compression bandaging, and ice in all groups	90	Routine serial ultrasound plus symptom-triggered CTPA	Aspirin, rivaroxaban, and dalteparin had similar reported thrombotic complication rates; aspirin had less blood loss, transfusion, and bleeding.
CRISTAL 2022 [1]	RCT	Open-label at hospitals/patients; investigators and DSMB masked during analysis	April 2019 to December 2020	Multicenter	Australia	9711	Mixed THA/TKA	Primary only	Unilateral and bilateral	Osteoarthritis	Within 24 h postoperatively (No lead-in period)	No lead-in period	14 d (TKA); 35 d (THA)	100	Enoxaparin (LMWH)	Enoxaparin 40 mg SC once daily for the same duration; 20 mg if weight <50 kg or eGFR <30	IPC calf devices until mobile, compression stockings, early mobilization in both groups	90	Symptom-driven; no routine VTE screening	Aspirin did not meet noninferiority and produced more symptomatic VTE than enoxaparin; secondary outcomes were not significantly better with enoxaparin.
Jato 2022 [17]	RCT	NA	NA	Single-center	Pakistan	160	THA only	Primary only	Unilateral	Osteoarthritis	Postoperative day 6 (Anticoagulant lead-in followed by aspirin)	Enoxaparin (LMWH)	16 d	100	Enoxaparin (LMWH) then Rivaroxaban	Enoxaparin 2000 U SC daily for 5 d, then rivaroxaban 10 mg orally daily for 16 d	Preoperative quadriceps and ankle exercises; no specific postoperative mechanical device reported	90	Routine duplex after 21 d; clinically relevant outcome reporting is inconsistent	The paper reports similar efficacy for aspirin and rivaroxaban after enoxaparin lead-in, but the numerical reporting is internally inconsistent.
Ren 2021 [21]	RCT	Allocation concealed; treatment blinding not reported	January 2019 to January 2020	Single-center	China	70	THA only	Primary only	Unilateral	Mixed etiologies including osteonecrosis and secondary osteoarthritis	Within 24 h postoperatively (No lead-in period)	No lead-in period	35 d	200	Rivaroxaban (DOAC)	Rivaroxaban 10 mg orally once daily for 5 wk	Pneumatic compression during hospitalization in both groups	90	Routine Doppler plus symptom monitoring	Aspirin 100 mg twice daily was not statistically different from rivaroxaban for VTE prevention or blood loss after primary THA.
Paudel 2020 [22]	RCT	Authors report coordinators, surgeons, and patients were blinded	May 2010 to May 2012	Single-center	India	180	THA only	Unclear	Unilateral or bilateral	Mixed, including inflammatory arthritis	Within 24 h postoperatively (No lead-in period)	No lead-in period	42 d	650	Enoxaparin (LMWH)	Enoxaparin 40 mg SC daily beginning 12 h after surgery and continued until mobilization (approximately postoperative day 4)	IPC was specified only for the aspirin arm	90	Routine serial Doppler plus symptom-triggered PE evaluation	Aspirin plus IPC had few thromboembolic events and substantially less major and minor bleeding than short-course LMWH.
Chung 2018 [23]	RCT	Open-label	May 2011-Nov 2013	Single-center	South Korea	278	TKA only	Primary only	Unilateral or staged bilateral	Osteoarthritis or rheumatoid arthritis	Postoperative day 3 (Anticoagulant lead-in followed by aspirin)	Dalteparin (LMWH)	5 d	100	Rivaroxaban (DOAC)	Rivaroxaban 10 mg orally daily for either 7 d or 10 d	Intermittent pneumatic compression for 7 d in all groups	90	Routine MDCT pulmonary angiography/indirect venography; symptoms classified separately	Rivaroxaban reduced overall imaging-detected VTE compared with the dalteparin-to-aspirin regimen, but symptomatic VTE did not differ; 10 d rivaroxaban was more effective than 7 d.
Colleoni 2018 [24]	RCT	Open-label	NA	Single-center	Brazil	32	TKA only	Primary only	Unilateral	Primary osteoarthritis	Within 24 h postoperatively (No lead-in period)	No lead-in period	14 d	300	Rivaroxaban (DOAC)	Rivaroxaban 10 mg orally once daily for 14 d	Standard rehabilitation; no specific mechanical prophylaxis reported	28	Clinical surveillance; Doppler only for suspected DVT	Aspirin and rivaroxaban were both considered useful, with no statistically significant differences in DVT or other complications.
EPCAT II 2018 [6]	RCT	Double-blind	January 2013 to April 2016	Multicenter	Canada	3424	Mixed THA/TKA	Mixed primary, revision, resurfacing, and other	Unilateral	Elective arthroplasty indications	Postoperative day 6 (Anticoagulant lead-in followed by aspirin)	Rivaroxaban (DOAC)	9 d (TKA); 30 d (THA)	81	Rivaroxaban (DOAC) continued	Rivaroxaban 10 mg orally daily continued for the same extended period	Mechanical prophylaxis varied by center and was not universal	90	Symptom-driven; no routine screening	After 5 d rivaroxaban, switching to aspirin was noninferior to continuing rivaroxaban for symptomatic VTE, with no significant bleeding difference.
Jiang 2014 [25]	RCT	Open-label	January 2012 to May 2013	Single-center	China	120	TKA only	Primary only	Unilateral	Degenerative arthritis	Within 24 h postoperatively (No lead-in period)	No lead-in period	14 d	100	LMWH then Rivaroxaban (DOAC)	LMWH 5000 U SC daily on postoperative days 1–5, then rivaroxaban 10 mg orally daily through day 14	Stockings and IPC in both groups; IPC to 5 d and stockings to 2 wk	42	Routine duplex screening plus clinical follow-up	Aspirin with mechanical prophylaxis had similar DVT incidence to sequential LMWH-rivaroxaban and was associated with less blood loss, ecchymosis, and cost.
Zou 2014 [26]	RCT	Open-label	July 2011 to July 2013	Single-center	China	324	TKA only	Primary only	Unilateral	Osteoarthritis	Within 24 h postoperatively (No lead-in period)	No lead-in period	14 d	100	LMWH	Rivaroxaban 10 mg orally daily or LMWH 4000 anti-Xa IU SC daily, beginning 12 h after surgery for 14 d	Elastic bandaging, elevation, ankle pumps, and early mobilization in all groups	28	Routine Doppler screening with symptomatic DVT identified separately; PE clinically assessed	Rivaroxaban reduced DVT more than aspirin or LMWH but caused more hidden blood loss and wound complications; aspirin and LMWH did not differ significantly.
EPCAT I 2013 [5]	RCT	Double-blind	December 2007 to July 2010	Multicenter	Canada	786	THA only	Mixed primary, resurfacing, and revision	Unilateral	Elective THA indications	Postoperative day 11 (Anticoagulant lead-in followed by aspirin)	Dalteparin (LMWH)	28 d	81	Dalteparin (LMWH) continued	Dalteparin 5000 U SC daily continued for another 28 d	Per local practice; not standardized in the main report	90	Symptom-driven; no routine screening	Extended aspirin after 10 d dalteparin was noninferior to continued dalteparin and had similar safety.
Gelfer 2006 [27]	RCT	Open-label; radiologist blinded	April 2001 to September 2002	Single-center	Israel	136	Mixed THA/TKA	Primary only	Unilateral	Elective arthroplasty indications	Within 24 h postoperatively (No lead-in period)	No lead-in period	In-hospital	100	Enoxaparin (LMWH)	Enoxaparin 40 mg SC once daily beginning 6–12 h after surgery	CECT was used only in the aspirin arm, creating an unequal mechanical co-intervention	90	Routine bilateral venography plus clinical surveillance	The CECT-plus-aspirin protocol produced fewer total and proximal DVTs than enoxaparin, with similar safety.
Westrich 2006 [28]	RCT	Open-label	September 2000 to February 2004	Single-center	United States	275	TKA only	Primary only	Unilateral	Elective TKA indications	Within 24 h postoperatively (No lead-in period)	No lead-in period	28 d	650	Enoxaparin (LMWH)	Enoxaparin 30 mg SC twice daily beginning approximately 48 h postoperatively until discharge, then 40 mg once daily for 3 wk, with VenaFlow	Spinal-epidural anesthesia and VenaFlow calf compression in both groups	42	Routine serial ultrasound; symptom-triggered CT for suspected PE	When combined with VenaFlow compression and neuraxial anesthesia, enoxaparin was not superior to aspirin for DVT prevention.

Abbreviations: anti–factor Xa (anti-Xa); computed tomography (CT); computed tomography pulmonary angiography (CTPA); continuous enhanced circulation therapy (CECT); data and safety monitoring board (DSMB); day(s) (d); deep vein thrombosis (DVT); direct oral anticoagulant (DOAC); estimated glomerular filtration rate (eGFR); hour(s) (h); international unit(s) (IU); intermittent pneumatic compression (IPC); low-molecular-weight heparin (LMWH); milligram(s) (mg); multidetector computed tomography (MDCT); not available (NA); pulmonary embolism (PE); randomized controlled trial (RCT); subcutaneous (SC); total hip arthroplasty (THA); total knee arthroplasty (TKA); unit(s) (U); venous thromboembolism (VTE); week(s) (wk). Study references: [1,5,6,7,17,18,19,20,21,22,23,24,25,26,27,28].

**Table 2:** Baseline characteristics of included patients.

Study ID	Group/Arm	Group Sample Size	Age, Years, Mean (SD)/Median [IQR]	Male Sex, *n* (%)	BMI, kg/m^2^, Mean (SD)/Median [IQR]	Surgery Type, *n* (%)		Surgery Class, *n* (%)				Bilateral Procedures, *n* (%)	Osteoarthritis, *n* (%)	Hypertension, *n* (%)	Diabetes Mellitus, *n* (%)	Previous VTE, *n* (%)	Cancer, *n* (%)	Current Smoker, *n* (%)	Hemoglobin, g/dL, Mean (SD)	Platelet Count, ×10^9^/L, Mean (SD)
						THA	TKA	Primary	Revision	Resurfacing	Other									
EPCAT III 2026 [7]	Aspirin	2718	66.7 (10.0)	1127 (41.5)	30.8 (6.8)	1269 (46.7)	1448 (53.3)	2626 (96.7)	91 (3.3)	0 (0.0)	0 (0.0)	0 (0.0)	NA	NA	NA	NA	16 (0.6)	NA	NA	NA
	Rivaroxaban then Aspirin	2647	66.3 (9.8)	1179 (44.7)	30.7 (6.3)	1247 (47.2)	1396 (52.8)	2554 (96.6)	89 (3.4)	0 (0.0)	0 (0.0)	0 (0.0)	NA	NA	NA	NA	9 (0.3)	NA	NA	NA
Campos-Flores 2024 [18]	Enoxaparin then Aspirin	30	NA	8 (26.7)	NA	5 (16.7)	25 (83.3)	30 (100.0)	0 (0.0)	0 (0.0)	0 (0.0)	NA	30 (100.0)	15 (50.0)	8 (26.7)	NA	NA	NA	NA	NA
	Enoxaparin	30	NA	9 (30.0)	NA	7 (23.3)	23 (76.7)	30 (100.0)	0 (0.0)	0 (0.0)	0 (0.0)	NA	30 (100.0)	18 (60.0)	10 (33.3)	NA	NA	NA	NA	NA
Kalia 2024 [20]	Rivaroxaban then Aspirin	50	60.0 (8.6)	22 (44.0)	NA	28 (56.0)	22 (44.0)	50 (100.0)		0 (0.0)	0 (0.0)	0 (0.0)	NA	NA	NA	NA	NA	NA	11.7 (1.7)	203.2 (68.9)
	Rivaroxaban continued	50	57.0 (11.1)	29 (58.0)	NA	33 (66.0)	17 (34.0)	50 (100.0)		0 (0.0)	0 (0.0)	0 (0.0)	NA	NA	NA	NA	NA	NA	11.2 (3.6)	213.8 (60.9)
Zhou 2023 [19]	Aspirin	60	66.4 (7.6)	26 (43.3)	25.6 (3.3)	0 (0.0)	60 (100.0)	60 (100.0)	0 (0.0)	0 (0.0)	0 (0.0)	0 (0.0)	60 (100.0)	NA	NA	NA	NA	NA	NA	NA
	Rivaroxaban	60	64.8 (7.2)	27 (45.0)	25.4 (4.2)	0 (0.0)	60 (100.0)	60 (100.0)	0 (0.0)	0 (0.0)	0 (0.0)	0 (0.0)	60 (100.0)	NA	NA	NA	NA	NA	NA	NA
	Dalteparin	60	64.1 (6.7)	29 (48.3)	24.9 (3.8)	0 (0.0)	60 (100.0)	60 (100.0)	0 (0.0)	0 (0.0)	0 (0.0)	0 (0.0)	60 (100.0)	NA	NA	NA	NA	NA	NA	NA
CRISTAL 2022 [1]	Aspirin	5675	67.0 [61.0–74.0]	2467 (43.5)	30.5 [26.9–35.1]	2155 (38.0)	3520 (62.0)	5675 (100.0)	0 (0.0)	0 (0.0)	0 (0.0)	636 (11.2)	5675 (100.0)	NA	NA	276 (4.9)	NA	NA	NA	NA
	Enoxaparin	4036	68.0 [61.0–74.0]	1733 (42.9)	30.6 [26.9–34.9]	1538 (38.1)	2498 (61.9)	4036 (100.0)	0 (0.0)	0 (0.0)	0 (0.0)	429 (10.6)	4036 (100.0)	NA	NA	240 (5.9)	NA	NA	NA	NA
Jato 2022 [17]	Enoxaparin then Aspirin	80	58.9 (8.2)	38 (47.5)	NA	80 (100.0)	0 (0.0)	80 (100.0)	0 (0.0)	0 (0.0)	0 (0.0)	0 (0.0)	80 (100.0)	NA	NA	NA	NA	NA	NA	NA
	Enoxaparin then Rivaroxaban	80	59.4 (9.9)	55 (68.8)	NA	80 (100.0)	0 (0.0)	80 (100.0)	0 (0.0)	0 (0.0)	0 (0.0)	0 (0.0)	80 (100.0)	NA	NA	NA	NA	NA	NA	NA
Ren 2021 [21]	Aspirin	34	54.5 [40.8–62.3]	13 (38.2)	23.6 [20.7–25.5]	34 (100.0)	0 (0.0)	34 (100.0)	0 (0.0)	0 (0.0)	0 (0.0)	0 (0.0)	NA	5 (14.7)	3 (8.8)	NA	NA	8 (23.5)	NA	NA
	Rivaroxaban	36	50.0 [36.8–57.0]	11 (30.6)	23.5 [20.3–26.0]	36 (100.0)	0 (0.0)	36 (100.0)	0 (0.0)	0 (0.0)	0 (0.0)	0 (0.0)	NA	6 (16.7)	1 (2.8)	NA	NA	5 (13.9)	NA	NA
Paudel 2020 [22]	Aspirin	90	48.3 (19.0–85.0)	48 (53.3)	NA	90 (100.0)	0 (0.0)	NA	NA	NA	NA	NA	NA	NA	NA	NA	NA	NA	NA	NA
	Enoxaparin	90	49.1 (19.0–85.0)	51 (56.7)	NA	90 (100.0)	0 (0.0)	NA	NA	NA	NA	NA	NA	NA	NA	NA	NA	NA	NA	NA
Chung 2018 [23]	Dalteparin then Aspirin	110	66.5 (32.0–83.0)	9 (8.2)	25.0 (16.2–33.8)	0 (0.0)	23 (76.7)	110 (100.0)	0 (0.0)	0 (0.0)	0 (0.0)	40 (36.4)	86 (78.2)	62 (56.4)	25 (22.7)	NA	NA	NA	NA	NA
	Rivaroxaban 7 d	110	66.6 (34.0–81.0)	7 (6.4)	24.7 (16.1–32.1)	0 (0.0)	110 (100.0)	110 (100.0)	0 (0.0)	0 (0.0)	0 (0.0)	36 (32.7)	96 (87.3)	64 (58.2)	29 (26.4)	NA	NA	NA	NA	NA
	Rivaroxaban 10 d	110	68.6 (39.0–84.0)	10 (9.1)	25.1 (18.8–34.4)	0 (0.0)	110 (100.0)	110 (100.0)	0 (0.0)	0 (0.0)	0 (0.0)	48 (43.6)	94 (85.5)	62 (56.4)	19 (17.3)	NA	NA	NA	NA	NA
Colleoni 2018 [24]	Aspirin	14	71.2 (6.4)	1 (7.1)	NA	0 (0.0)	14 (100.0)	14 (100.0)	0 (0.0)	0 (0.0)	0 (0.0)	0 (0.0)	14 (100.0)	NA	NA	NA	NA	NA	NA	NA
	Rivaroxaban	18	67.1 (7.7)	4 (22.2)	NA	0 (0.0)	18 (100.0)	18 (100.0)	0 (0.0)	0 (0.0)	0 (0.0)	0 (0.0)	18 (100.0)	NA	NA	NA	NA	NA	NA	NA
EPCAT II 2018 [6]	Rivaroxaban then Aspirin	1707	62.9 (10.1)	804 (47.1)	31.1 (6.8)	902 (52.8)	805 (47.2)	1569 (91.9)	94 (5.5)	41 (2.4)	3 (0.2)	0 (0.0)	NA	NA	NA	37 (2.2)	42 (2.5)	162 (9.5)	13.9 (1.3)	241.2
	Rivaroxaban continued	1717	62.7 (10.1)	833 (48.5)	31.0 (6.6)	902 (52.5)	815 (47.5)	1572 (91.6)	108 (6.3)	35 (2.0)	2 (0.1)	0 (0.0)	NA	NA	NA	44 (2.6)	38 (2.2)	157 (9.1)	13.9 (1.3)	240.9
Jiang 2014 [25]	Aspirin	60	65.1 (7.5)	5 (8.3)	24.2 (3.3)	0 (0.0)	60 (100.0)	60 (100.0)	0 (0.0)	0 (0.0)	0 (0.0)	0 (0.0)	60 (100.0)	NA	NA	NA	NA	NA	NA	NA
	LMWH then Rivaroxaban	60	63.8 (6.7)	4 (6.7)	24.6 (3.6)	0 (0.0)	60 (100.0)	60 (100.0)	0 (0.0)	0 (0.0)	0 (0.0)	0 (0.0)	60 (100.0)	NA	NA	NA	NA	NA	NA	NA
Zou 2014 [26]	Aspirin	110	62.7 (47.0–79.0)	28 (25.5)	27.8 (17.8–40.0)	0 (0.0)	110 (100.0)	110 (100.0)	0 (0.0)	0 (0.0)	0 (0.0)	0 (0.0)	110 (100.0)	NA	NA	NA	NA	NA	13	NA
	Rivaroxaban	102	63.5 (50.0–82.0)	32 (31.4)	27.5 (18.0–39.5)	0 (0.0)	102 (100.0)	102 (100.0)	0 (0.0)	0 (0.0)	0 (0.0)	0 (0.0)	102 (100.0)	NA	NA	NA	NA	NA	13.1	NA
	LMWH	112	65.7 (54.0–80.0)	20 (17.9)	27.0 (20.3–37.0)	0 (0.0)	112 (100.0)	112 (100.0)	0 (0.0)	0 (0.0)	0 (0.0)	0 (0.0)	112 (100.0)	NA	NA	NA	NA	NA	12.9	NA
EPCAT I 2013 [5]	Dalteparin then Aspirin	385	57.6 (11.9)	231 (60.0)	29.3 (5.9)	385 (100.0)	0 (0.0)	308 (80.0)	35 (9.1)	42 (10.9)	0 (0.0)	0 (0.0)	NA	137 (35.6)	33 (8.6)	5 (1.3)	8 (2.1)	53 (13.8)	14.2	246.7
	Dalteparin continued	400	57.9 (12.2)	213 (53.3)	27.9 (5.8)	400 (100.0)	0 (0.0)	320 (80.0)	35 (8.8)	45 (11.3)	0 (0.0)	0 (0.0)	NA	144 (36.0)	30 (7.5)	8 (2.0)	13 (3.3)	64 (16.0)	14.1	258.6
Gelfer 2006 [27]	Aspirin	61	68.0 (10.4)	21 (34.4)	28.0 (4.0)	33 (54.1)	28 (45.9)	61 (100.0)	0 (0.0)	0 (0.0)	0 (0.0)	0 (0.0)	NA	26 (42.6)	3 (4.9)	NA	5 (8.2)	10 (16.4)	NA	NA
	Enoxaparin	60	67.0 (8.7)	23 (38.3)	29.0 (4.8)	40 (66.7)	20 (33.3)	60 (100.0)	0 (0.0)	0 (0.0)	0 (0.0)	0 (0.0)	NA	26 (43.3)	10 (16.7)	NA	8 (13.3)	6 (10.0)	NA	NA
Westrich 2006 [28]	Aspirin	136	69.0 (12.1)	53 (39.0)	NA	0 (0.0)	136 (100.0)	136 (100.0)	0 (0.0)	0 (0.0)	0 (0.0)	0 (0.0)	NA	NA	NA	NA	NA	NA	NA	NA
	Enoxaparin	139	68.9 (9.6)	46 (33.1)	NA	0 (0.0)	139 (100.0)	139 (100.0)	0 (0.0)	0 (0.0)	0 (0.0)	0 (0.0)	NA	NA	NA	NA	NA	NA	NA	NA

Data are presented as *n* (%) unless otherwise indicated. Age and body mass index are reported as mean (SD), median [IQR], or mean (range), according to the statistic provided by each study. Group sample size represents patients, except in Chung et al. [23], which reported TKA cases/procedures; percentage denominators may therefore differ from the group sample size when baseline data were missing. Hemoglobin values were standardized to g/dL by dividing values originally reported in g/L by 10, while platelet counts were standardized to ×10^9^/L; values reported as ×10^3^/µL are numerically equivalent. For trials explicitly restricted to primary arthroplasty, surgery class was recorded as 100% primary even when arm-specific counts were not separately reported. Kalia et al. [20] included both primary and revision arthroplasty but did not report their distribution, whereas Paudel et al. [22] did not report primary/revision status. Abbreviations: body mass index (BMI); continuous enhanced circulation therapy (CECT); interquartile range (IQR); intermittent pneumatic compression (IPC); not available (NA); standard deviation (SD); total hip arthroplasty (THA); total knee arthroplasty (TKA); twice daily (BID); venous thromboembolism (VTE). Study references: [1,5,6,7,17,18,19,20,21,22,23,24,25,26,27,28].

## Data Availability

All data generated or analysed during this study are included in the article and its Supplementary Materials.

## Supplementary Materials

The following supporting information can be downloaded at: https://www.mdpi.com/article/10.3390/medicina62102030/s1, Supplementary Table S1 presents the complete database search strategies and results. Supplementary Figures S1–S53 present subgroup, sensitivity, heterogeneity, reporting-bias, secondary-outcome, and safety analyses, including the additional risk-difference analyses incorporating CRISTAL’s published cluster-adjusted estimates [1,5,6,19,20,21,22,23,24,25,26,27,28]. Supplementary File S1 contains the completed PRISMA 2020 checklist [12].
