# Supplementary material for: Aspirin-Based Thromboprophylaxis Versus Anticoagulant Regimens After Total Hip or Knee Arthroplasty: An Updated Systematic Review and Meta-Analysis of Randomized Controlled Trials
Source: Medicina (Kaunas). 2026 Oct 20;62(10):2030. doi: 10.3390/medicina62102030 (PMC13707184; doi:10.3390/medicina62102030)
Supplement: Supplementary file 1 [file medicina-62-02030-s001.zip › medicina-4623786-supplementary.pdf]

Table of Contents

Table S1. Search terms and results in different databases. 1
Figure S1. Forest plot of subgroup analysis of any DVT by aspirin initiation timing. 6
Figure S2. Forest plot of subgroup analysis of any DVT by intervention regimen. 7
Figure S3. Forest plot of subgroup analysis of any DVT by procedure laterality. 8
Figure S4. Forest plot of subgroup analysis of any DVT by surgery class. 9
Figure S5. Forest plot of subgroup analysis of any DVT by surgery type. 10
Figure S6. Forest plot of subgroup analysis of any DVT by total daily aspirin dose. 11
Figure S7. Forest plot of subgroup analysis of any DVT by follow-up duration. 12
Figure S8. Leave-one-out analysis of any DVT. 13
Figure S9. Galbraith plot for any DVT. 14
Figure S10. Funnel plot for any DVT. 15
Figure S11. Forest plot of subgroup analysis of symptomatic DVT by aspirin initiation timing. 16
Figure S12. Forest plot of subgroup analysis of symptomatic DVT by intervention regimen. 17
Figure S13. Forest plot of subgroup analysis of symptomatic DVT by procedure laterality. 18
Figure S14. Forest plot of subgroup analysis of symptomatic DVT by surgery class. 19
Figure S15. Forest plot of subgroup analysis of symptomatic DVT by surgery type. 20
Figure S16. Forest plot of subgroup analysis of symptomatic DVT by total daily aspirin dose. 21
Figure S17. Forest plot of subgroup analysis of symptomatic DVT by follow-up duration. 22
Figure S18. Leave-one-out analysis of symptomatic DVT. 23
Figure S19. Galbraith plot for symptomatic DVT. 24
Figure S20. Doi plot and LFK index for symptomatic DVT. 25
Figure S21. Forest plot of subgroup analysis of symptomatic VTE by aspirin initiation timing. 26
Figure S22. Forest plot of subgroup analysis of symptomatic VTE by intervention regimen. 27
Figure S23. Forest plot of subgroup analysis of symptomatic VTE by procedure laterality. 28
Figure S24. Forest plot of subgroup analysis of symptomatic VTE by surgery class. 29
Figure S25. Forest plot of subgroup analysis of symptomatic VTE by surgery type. 30
Figure S26. Forest plot of subgroup analysis of symptomatic VTE by total daily aspirin dose. 31
Figure S27. Forest plot of subgroup analysis of symptomatic VTE by follow-up duration. 32
Figure S28. Leave-one-out analysis of symptomatic VTE. 33
Figure S29. Galbraith plot for symptomatic VTE. 34
Figure S30. Funnel plot for symptomatic VTE. 35
Figure S31. Forest plot of proximal deep vein thrombosis. 36
Figure S32. Forest plot of distal deep vein thrombosis. 37
Figure S33. Forest plot of major bleeding. 38
Figure S34. Forest plot of clinically relevant non-major bleeding. 39
Figure S35. Forest plot of minor bleeding. 40
Figure S36. Forest plot of any bleeding 41
Figure S37. Forest plot of ecchymosis or bruising. 42
Figure S38. Forest plot of wound infection. 43
Figure S39. Forest plot of wound hematoma. 44
Figure S40. Forest plot of gastrointestinal bleeding. 45
Figure S41. Forest plot of blood transfusion. 46
Figure S42. Forest plot of mortality. 47
Figure S43. Forest plot of Low risk symptomatic VTE 48
Figure S44. Forest plot of Low risk symptomatic DVT 49
Figure S45. Forest plot of Low risk any DVT 50
Figure S46. Forest plot of Pulmonary embolism excluding CRISTAL 51

Figure S47. Forest plot of any deep vein thrombosis stratified by total pharmacological prophylaxis duration (≤14 versus >14 days) 52
Figure S48. Forest plot of symptomatic deep vein thrombosis stratified by total pharmacological prophylaxis duration (≤14 versus >14 days) 53
Figure S49. Forest plot of symptomatic venous thromboembolism stratified by total pharmacological prophylaxis duration (≤14 versus >14 days) 54
Figure S50. Any DVT with CRISTAL cluster adjustment. 55
Figure S51. Symptomatic DVT with CRISTAL cluster adjustment. 56
Figure S52. Symptomatic VTE with CRISTAL cluster adjustment. 57
Figure S53. PE with CRISTAL cluster adjustment. 58

Table S1. Search terms and results in different databases.

Database	Search query	Filter	Search results
PubMed	(<br>"Arthroplasty, Replacement, Hip"[Mesh]<br>OR "Arthroplasty, Replacement, Knee"[Mesh]<br>OR "Hip Prosthesis"[Mesh]<br>OR "Knee Prosthesis"[Mesh]<br>OR (<br>("Arthroplasty, Replacement"[Mesh] OR "Joint Prosthesis"[Mesh])<br>AND<br>("Hip Joint"[Mesh] OR "Knee Joint"[Mesh])<br>)<br>OR (<br>(hip[tiab] OR hips[tiab])<br>AND<br>(arthroplast*[tiab] OR replacement*[tiab] OR prosthe*[tiab])<br>)<br>OR (<br>(knee[tiab] OR knees[tiab])<br>AND<br>(arthroplast*[tiab] OR replacement*[tiab] OR prosthe*[tiab])<br>)<br>OR (<br>(joint[tiab] OR joints[tiab])<br>AND<br>(arthroplast*[tiab] OR replacement*[tiab])<br>)<br>OR "total hip arthroplasty"[tiab]<br>OR "total knee arthroplasty"[tiab]<br>OR "total hip replacement"[tiab]<br>OR "total knee replacement"[tiab]<br>OR "total joint arthroplasty"[tiab]	All feilds	863

	OR "total joint replacement"[tiab]<br>OR THA[tiab]<br>OR TKA[tiab]<br>OR THR[tiab]<br>OR TKR[tiab]<br>)<br>AND<br>(<br>"Aspirin"[Mesh]<br>OR aspirin[tiab]<br>OR acetylsalicyl*[tiab]<br>OR "acetyl salicylic acid"[tiab]<br>OR "ASA prophylaxis"[tiab]<br>)		
Scopus	TITLE-ABS-KEY(<br>(<br>(hip W/3 arthroplast*)<br>OR (hip W/3 replacement*)<br>OR (hip W/3 prosthe*)<br>OR (hips W/3 arthroplast*)<br>OR (hips W/3 replacement*)<br>OR (hips W/3 prosthe*)<br>OR (knee W/3 arthroplast*)<br>OR (knee W/3 replacement*)<br>OR (knee W/3 prosthe*)<br>OR (knees W/3 arthroplast*)<br>OR (knees W/3 replacement*)<br>OR (knees W/3 prosthe*)<br>OR (joint W/3 arthroplast*)<br>OR (joint W/3 replacement*)<br>OR (joints W/3 arthroplast*)<br>OR (joints W/3 replacement*)<br>OR "total hip arthroplasty"<br>OR "total knee arthroplasty"<br>OR "total hip replacement"<br>OR "total knee replacement"<br>OR "total joint arthroplasty"<br>OR "total joint replacement"<br>OR THA<br>OR TKA<br>OR THR<br>OR TKR<br>)<br>AND<br>(<br>aspirin	Article title, Abstract, Keywords	2,595

	OR acetylsalicyl*<br>OR "acetyl salicylic acid"<br>OR "ASA prophylaxis"<br>OR "ASA thromboprophylaxis"<br>OR "ASA chemoprophylaxis"<br>)<br>)		
Web of Science	TS=(<br>(<br>(hip NEAR/3 arthroplast*)<br>OR (hip NEAR/3 replacement*)<br>OR (hip NEAR/3 prosthe*)<br>OR (hips NEAR/3 arthroplast*)<br>OR (hips NEAR/3 replacement*)<br>OR (hips NEAR/3 prosthe*)<br>OR (knee NEAR/3 arthroplast*)<br>OR (knee NEAR/3 replacement*)<br>OR (knee NEAR/3 prosthe*)<br>OR (knees NEAR/3 arthroplast*)<br>OR (knees NEAR/3 replacement*)<br>OR (knees NEAR/3 prosthe*)<br>OR (joint NEAR/3 arthroplast*)<br>OR (joint NEAR/3 replacement*)<br>OR (joints NEAR/3 arthroplast*)<br>OR (joints NEAR/3 replacement*)<br>OR "total hip arthroplasty"<br>OR "total knee arthroplasty"<br>OR "total hip replacement"<br>OR "total knee replacement"<br>OR "total joint arthroplasty"<br>OR "total joint replacement"<br>OR THA<br>OR TKA<br>OR THR<br>OR TKR<br>)<br>AND<br>(<br>aspirin<br>OR acetylsalicyl*<br>OR "acetyl salicylic acid"<br>OR "ASA prophylaxis"<br>OR "ASA thromboprophylaxis"<br>OR "ASA chemoprophylaxis"<br>)<br>)	Topic	938

Cochrane central	#1<br>MeSH descriptor: [Arthroplasty, Replacement, Hip] explode all trees<br>#2<br>MeSH descriptor: [Arthroplasty, Replacement, Knee] explode all trees<br>#3<br>MeSH descriptor: [Hip Prosthesis] explode all trees<br>#4<br>MeSH descriptor: [Knee Prosthesis] explode all trees<br>#5<br>((hip or hips) near/3 (arthroplast* or replacement* or prosthe*)):ti,ab,kw<br>#6<br>((knee or knees) near/3 (arthroplast* or replacement* or prosthe*)):ti,ab,kw<br>#7<br>((joint or joints) near/3 (arthroplast* or replacement*)):ti,ab,kw<br>#8<br>(<br>"total hip arthroplasty"<br>or "total knee arthroplasty"<br>or "total hip replacement"<br>or "total knee replacement"<br>or "total joint arthroplasty"<br>or "total joint replacement"<br>or THA<br>or TKA<br>or THR<br>or TKR<br>):ti,ab,kw<br>#9<br>#1 or #2 or #3 or #4 or #5 or #6 or #7 or #8<br>#10<br>MeSH descriptor: [Aspirin] explode all trees<br>#11<br>(<br>aspirin<br>or acetylsalicyl*<br>or "acetyl salicylic acid"<br>or "ASA prophylaxis"<br>or "ASA thromboprophylaxis"<br>or "ASA chemoprophylaxis"	Title, Abstract, Keyword	226

	):ti,ab,kw<br>#12<br>#10 or #11<br>#13<br>#9 and #12		
Total			4,622
After Duplicate Removing (By EndNote 21)			3,430

Search date: 25/7/2026

Figure S1. Forest plot of subgroup analysis of any DVT by aspirin initiation timing.

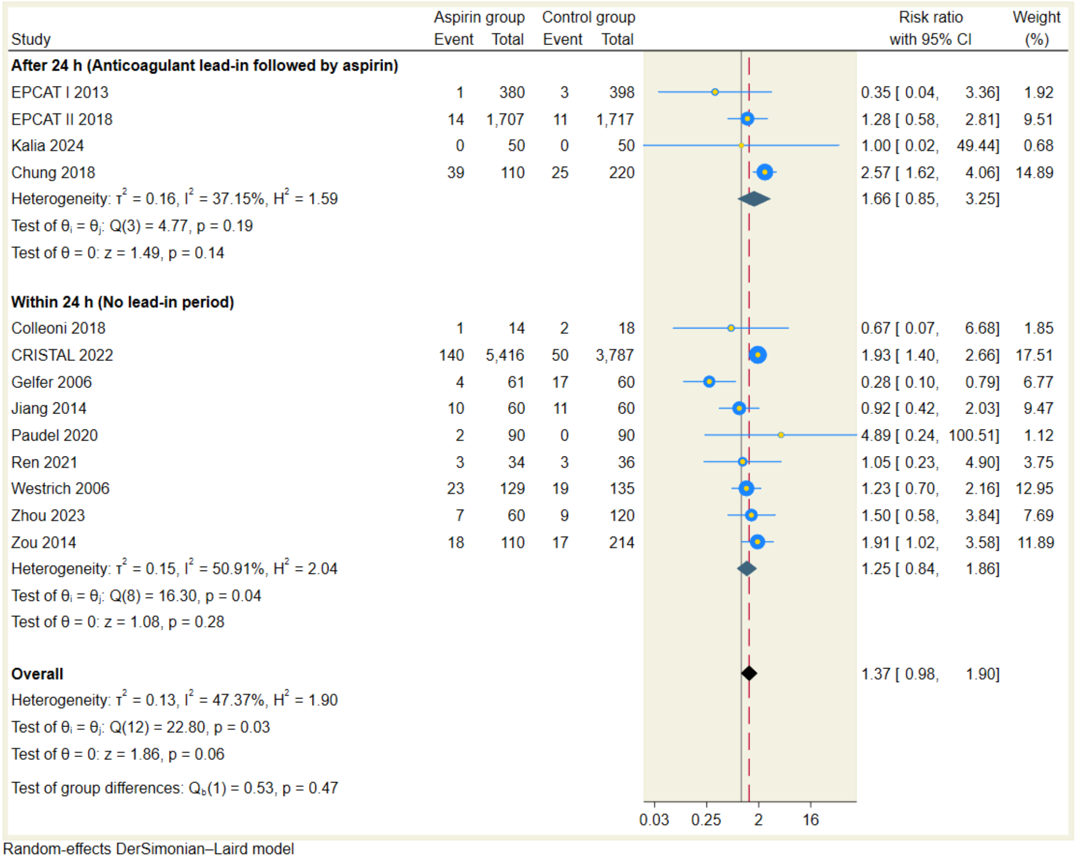

Figure S1. Forest plot of subgroup analysis of any DVT by aspirin initiation timing. Pooled risk ratios were calculated using a random-effects model. CI, confidence interval; DVT, deep vein thrombosis; RR, risk ratio; VTE, venous thromboembolism. Study references: [1,5,6,19–28].

Figure S2. Forest plot of subgroup analysis of any DVT by intervention regimen.

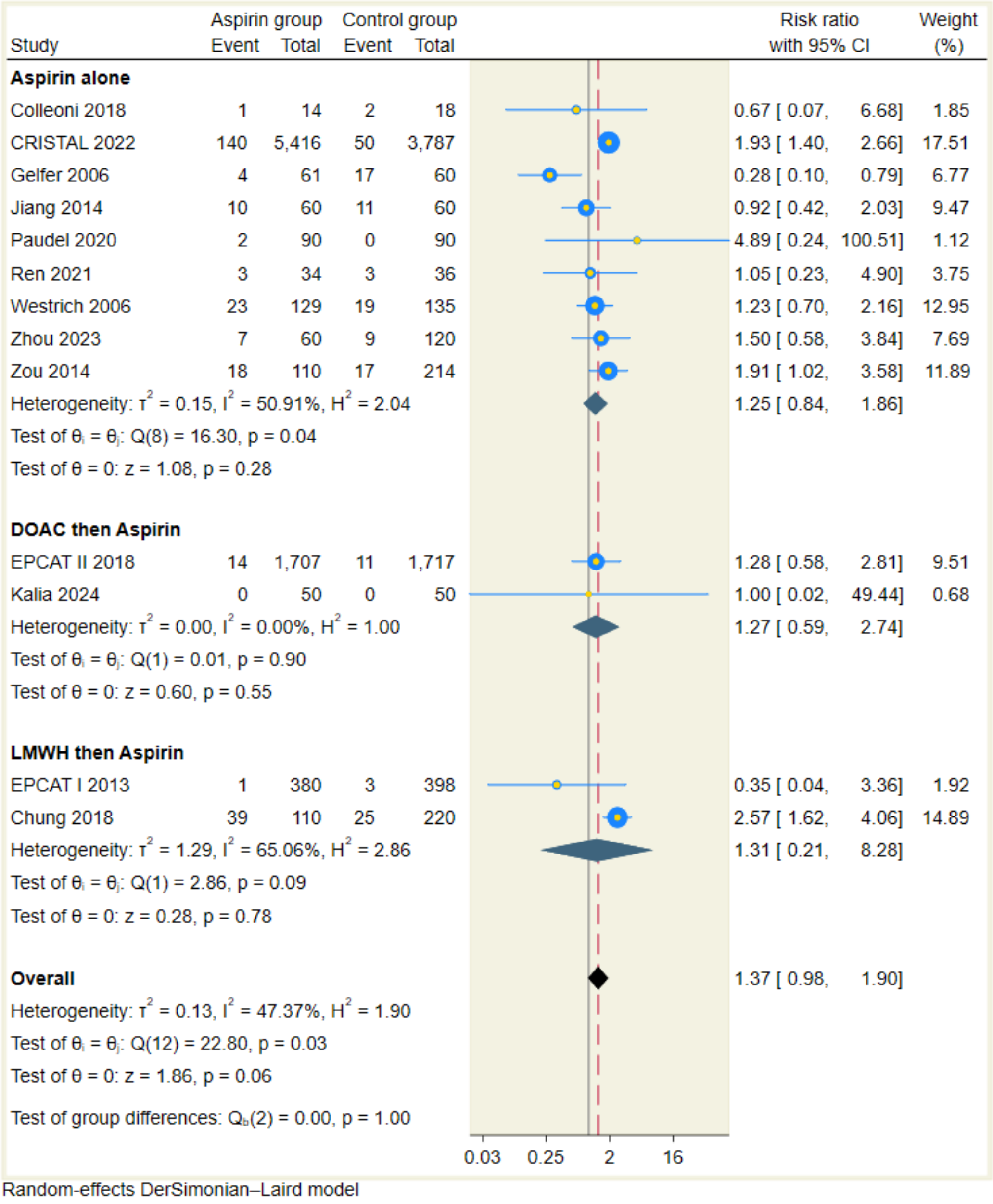

Figure S2. Forest plot of subgroup analysis of any DVT by intervention regimen. Pooled risk ratios were calculated using a random-effects model. CI, confidence interval; DVT, deep vein thrombosis; RR, risk ratio; VTE, venous thromboembolism. Study references: [1,5,6,19–28].

Figure S3. Forest plot of subgroup analysis of any DVT by procedure laterality.

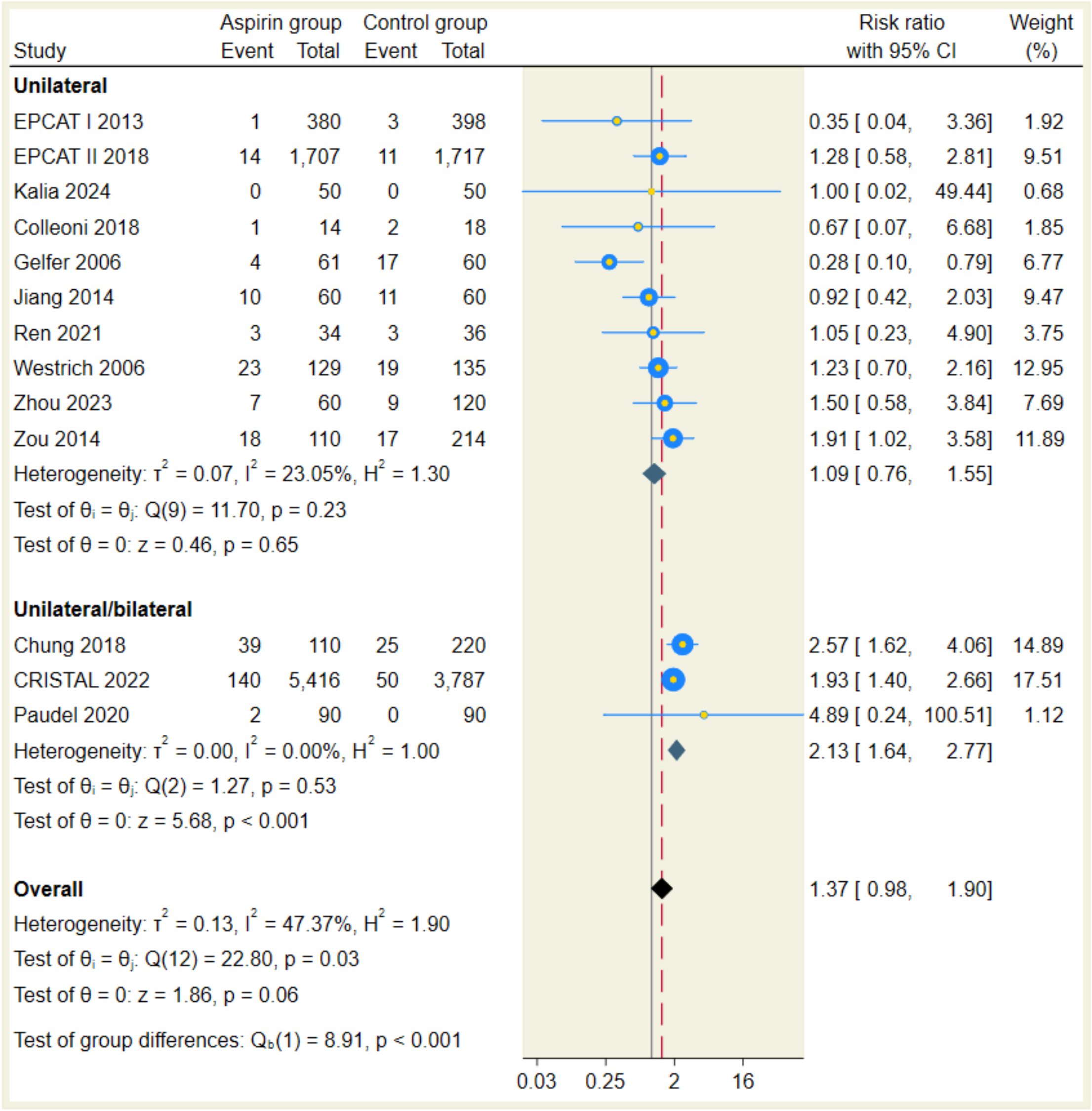

Random-effects DerSimonian–Laird model

Figure S3. Forest plot of subgroup analysis of any DVT by procedure laterality. Pooled risk ratios were calculated using a random-effects model. CI, confidence interval; DVT, deep vein thrombosis; RR, risk ratio; VTE, venous thromboembolism. Study references: [1,5,6,19–28].

Figure S4. Forest plot of subgroup analysis of any DVT by surgery class.

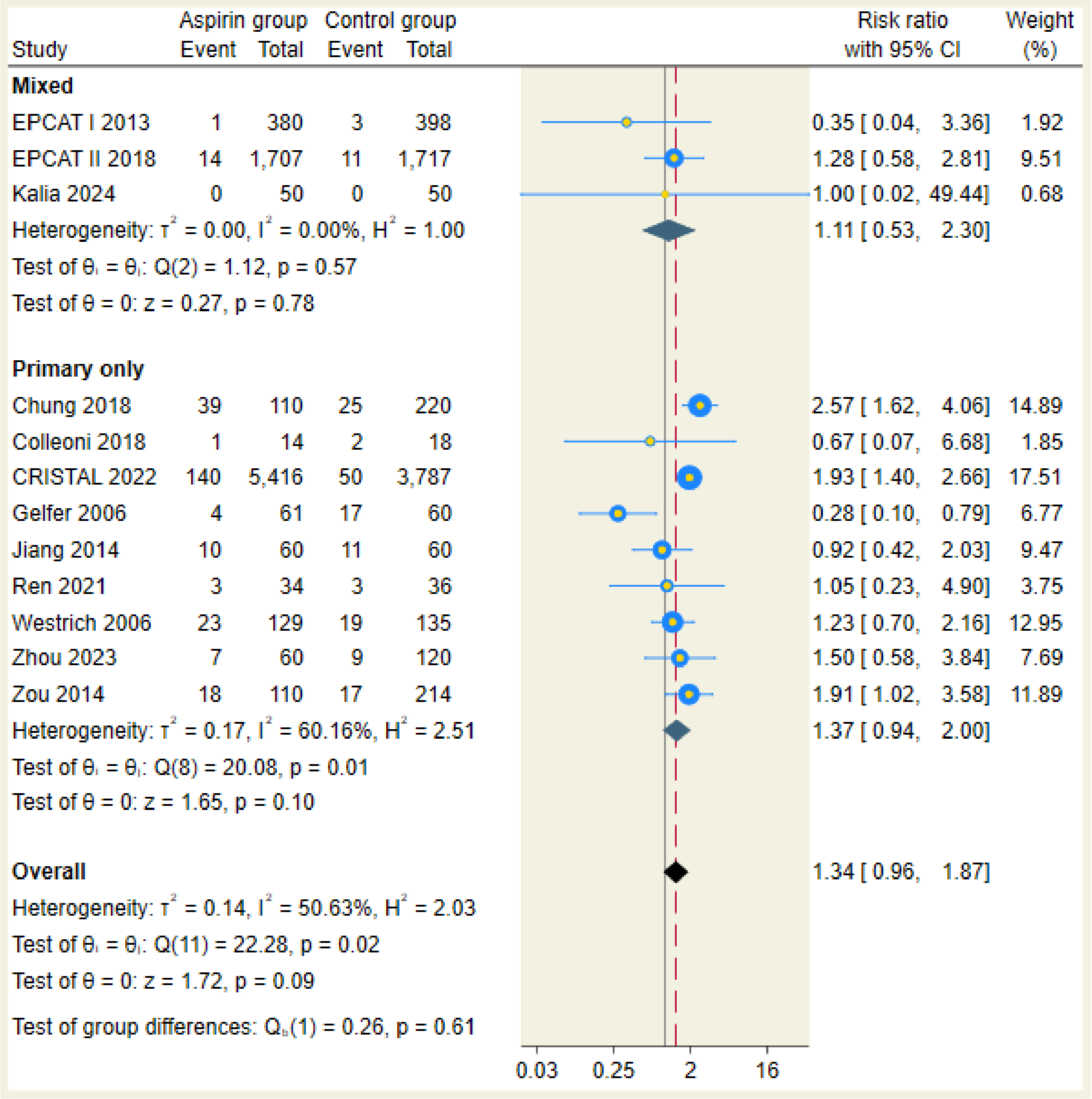

Random-effects DerSimonian–Laird model

Figure S4. Forest plot of subgroup analysis of any DVT by surgery class. Pooled risk ratios were calculated using a random-effects model. CI, confidence interval; DVT, deep vein thrombosis; RR, risk ratio; VTE, venous thromboembolism. Study references: [1,5,6,19–28].

Figure S5. Forest plot of subgroup analysis of any DVT by surgery type.

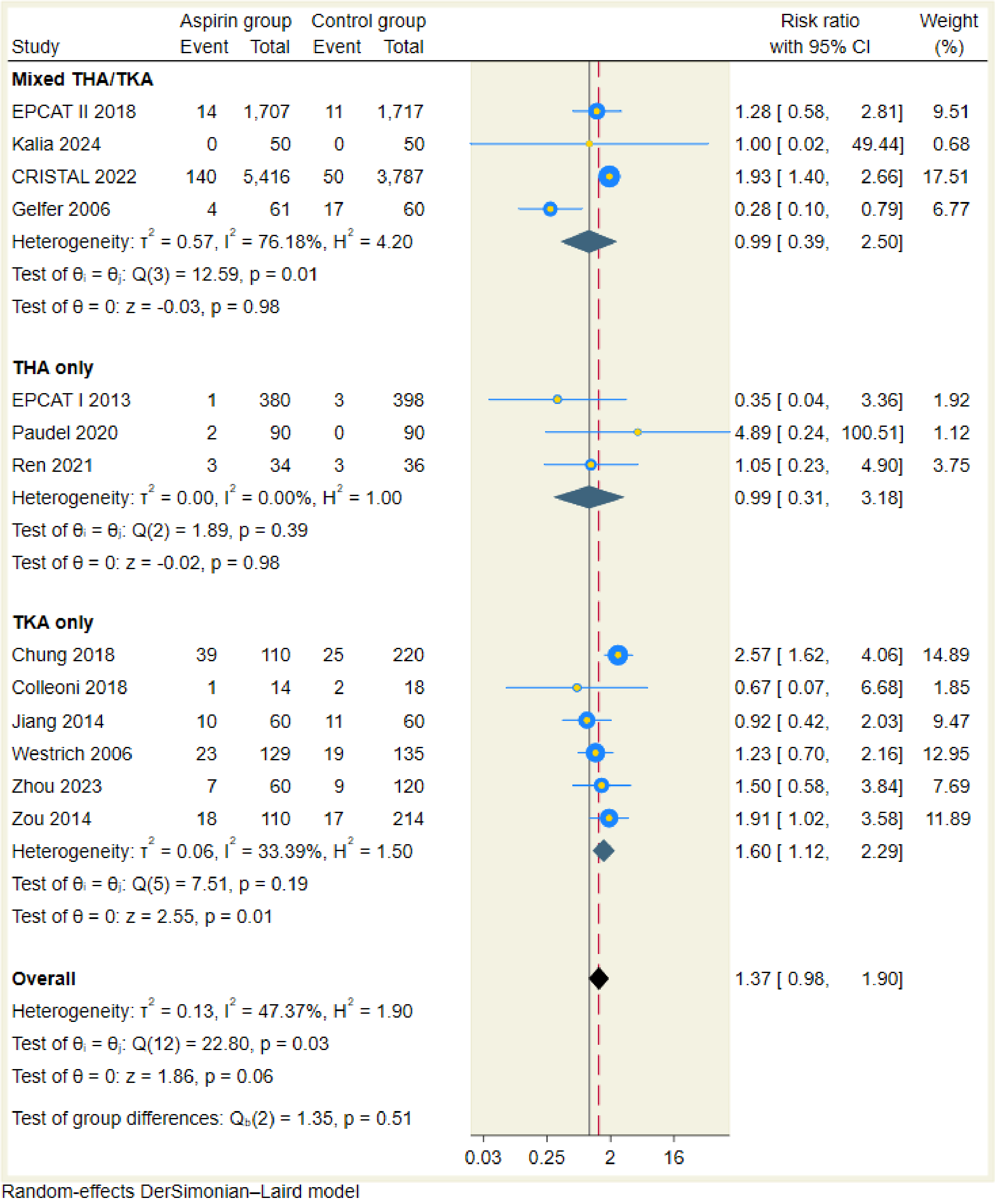

Figure S5. Forest plot of subgroup analysis of any DVT by surgery type. Pooled risk ratios were calculated using a random-effects model. CI, confidence interval; DVT, deep vein thrombosis; RR, risk ratio; VTE, venous thromboembolism. Study references: [1,5,6,19–28].

Figure S6. Forest plot of subgroup analysis of any DVT by total daily aspirin dose.

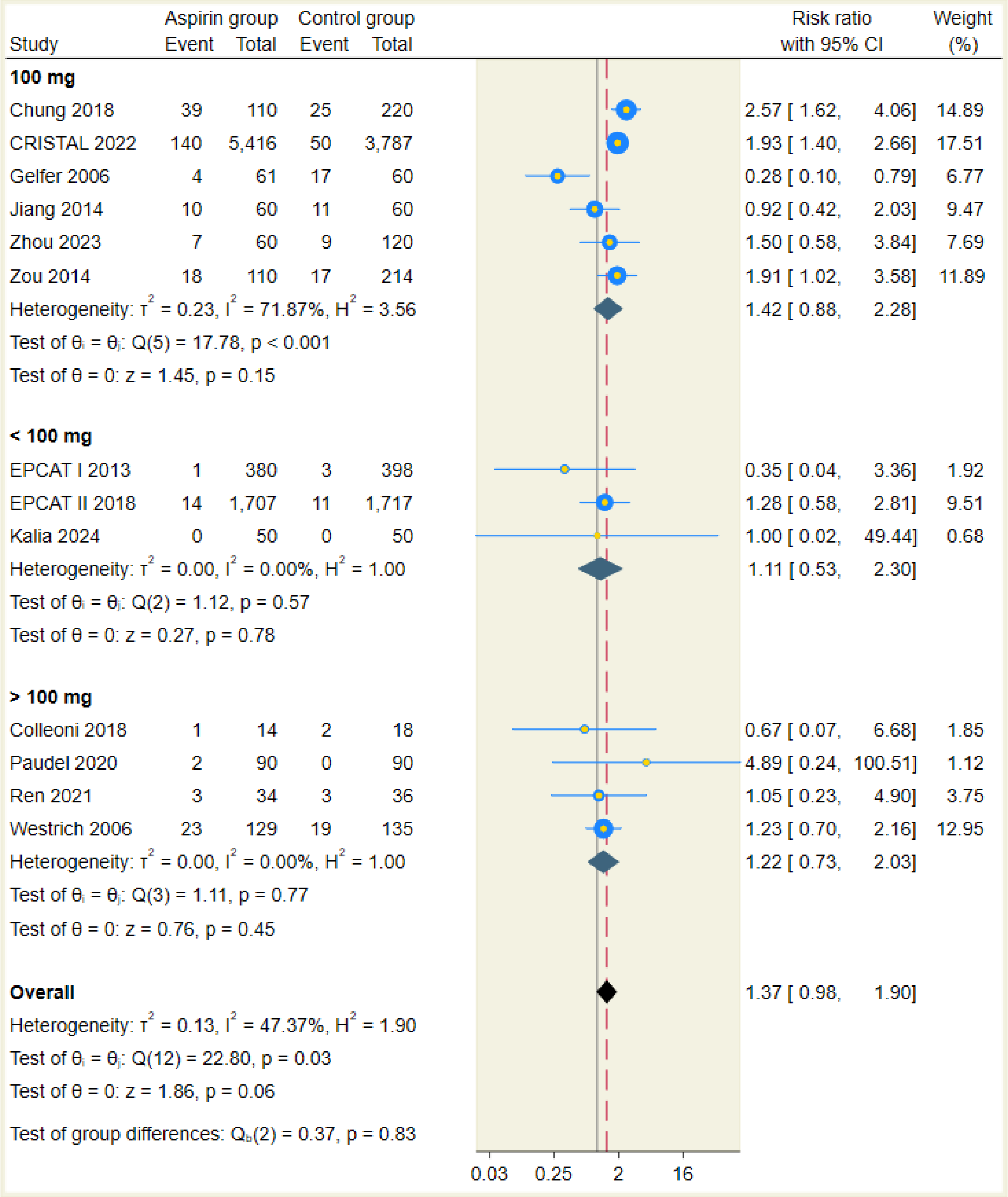

Random-effects DerSimonian–Laird model

Figure S6. Forest plot of subgroup analysis of any DVT by total daily aspirin dose. Pooled risk ratios were calculated using a random-effects model. CI, confidence interval; DVT, deep vein thrombosis; RR, risk ratio; VTE, venous thromboembolism. Study references: [1,5,6,19–28].

Figure S7. Forest plot of subgroup analysis of any DVT by follow-up duration.

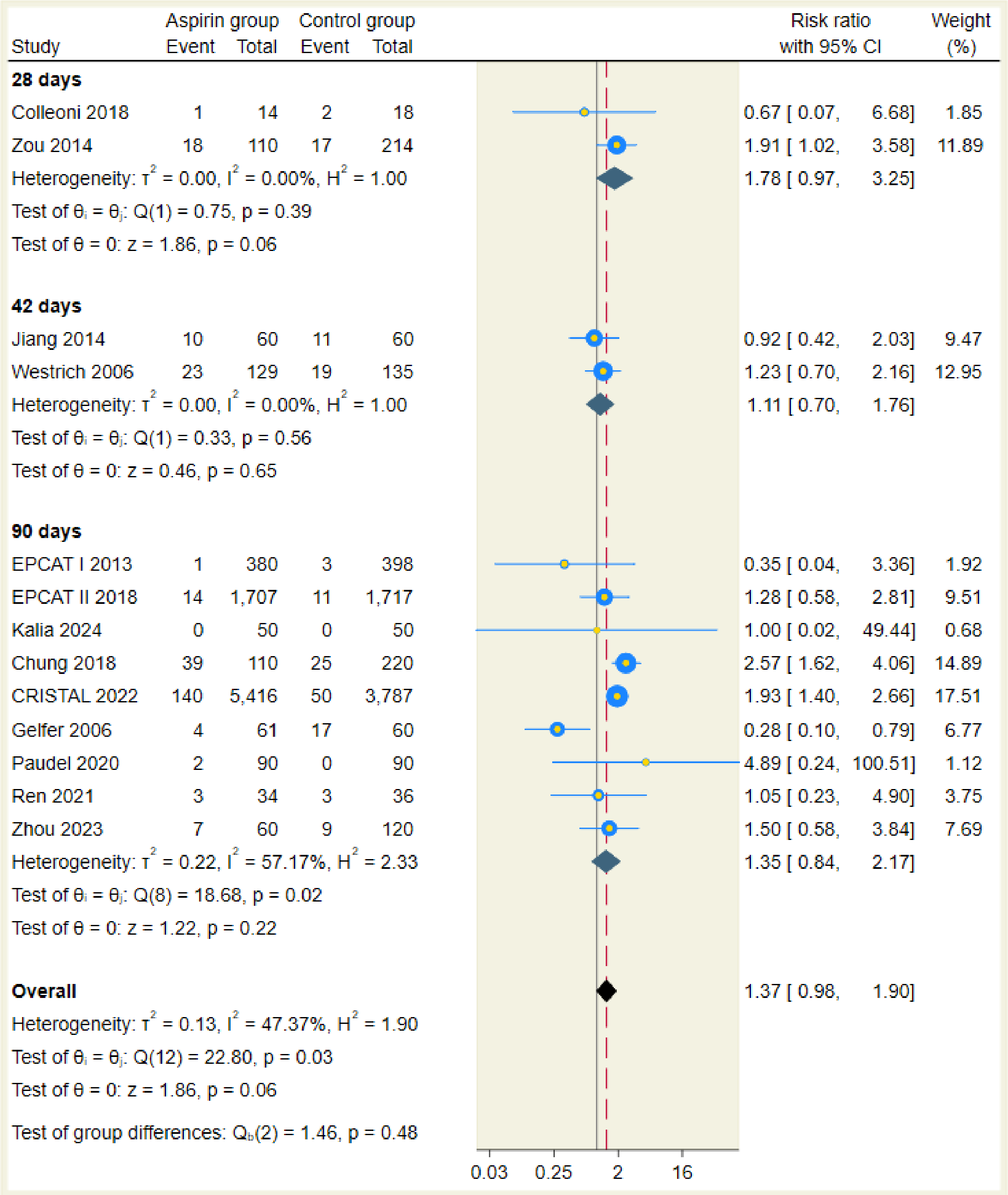

Random-effects DerSimonian–Laird model

Figure S7. Forest plot of subgroup analysis of any DVT by follow-up duration. Pooled risk ratios were calculated using a random-effects model. CI, confidence interval; DVT, deep vein thrombosis; RR, risk ratio; VTE, venous thromboembolism. Study references: [1,5,6,19–28].

Figure S8. Leave-one-out analysis of any DVT.

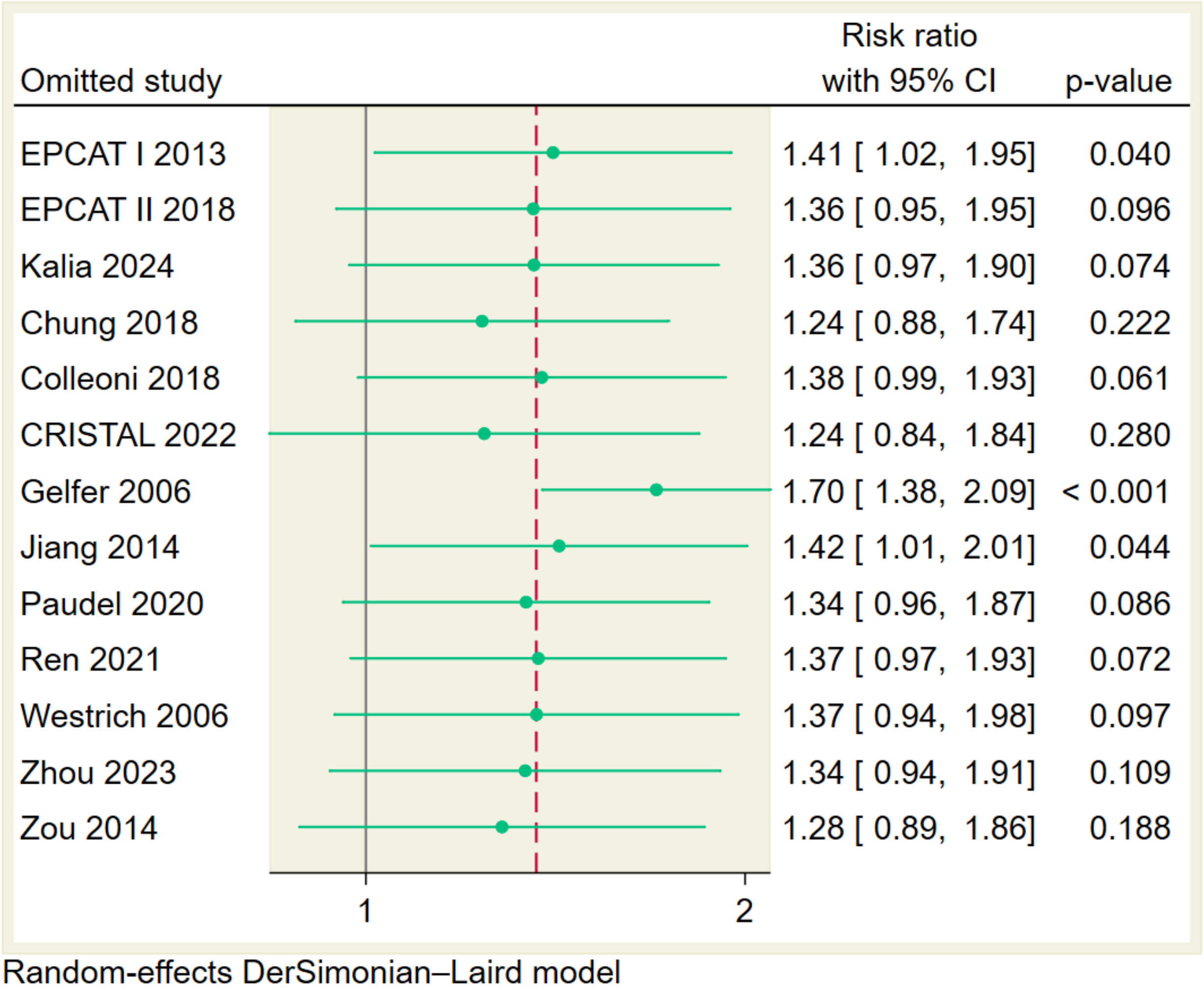

Figure S8. Leave-one-out analysis of any DVT. The pooled risk ratio was recalculated after omitting one study at a time. Study references: [1,5,6,19–28].

Figure S9. Galbraith plot for any DVT.

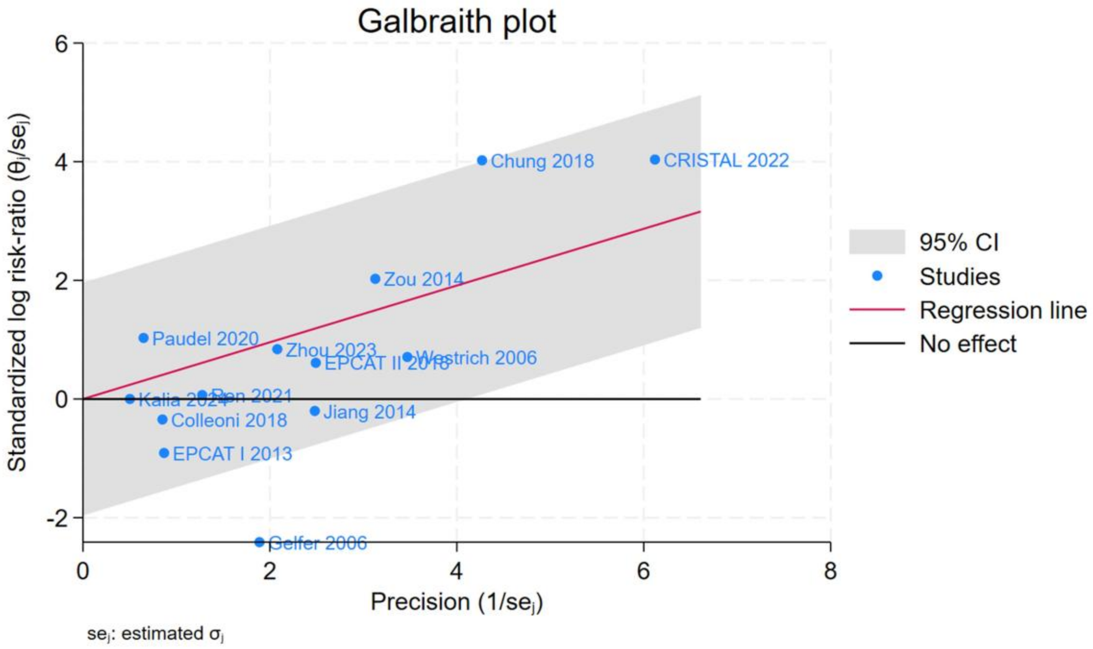

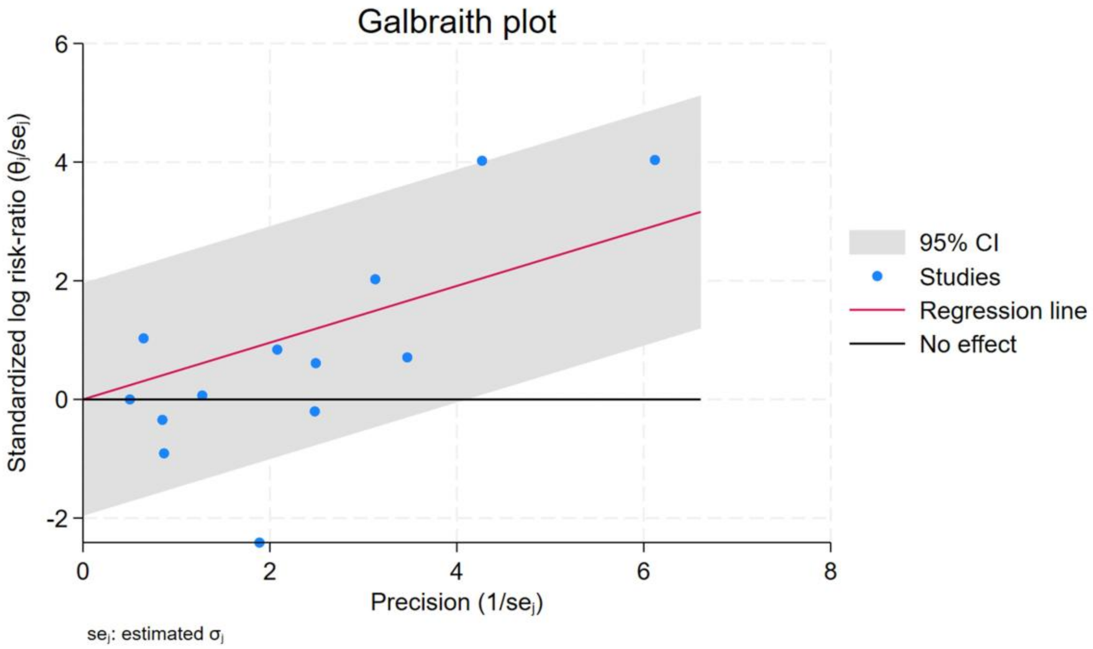

Figure S9. Galbraith plot for any DVT. Radial plot used to explore potential outlying or influential studies. Study references: [1,5,6,19–28].

Figure S10. Funnel plot for any DVT.

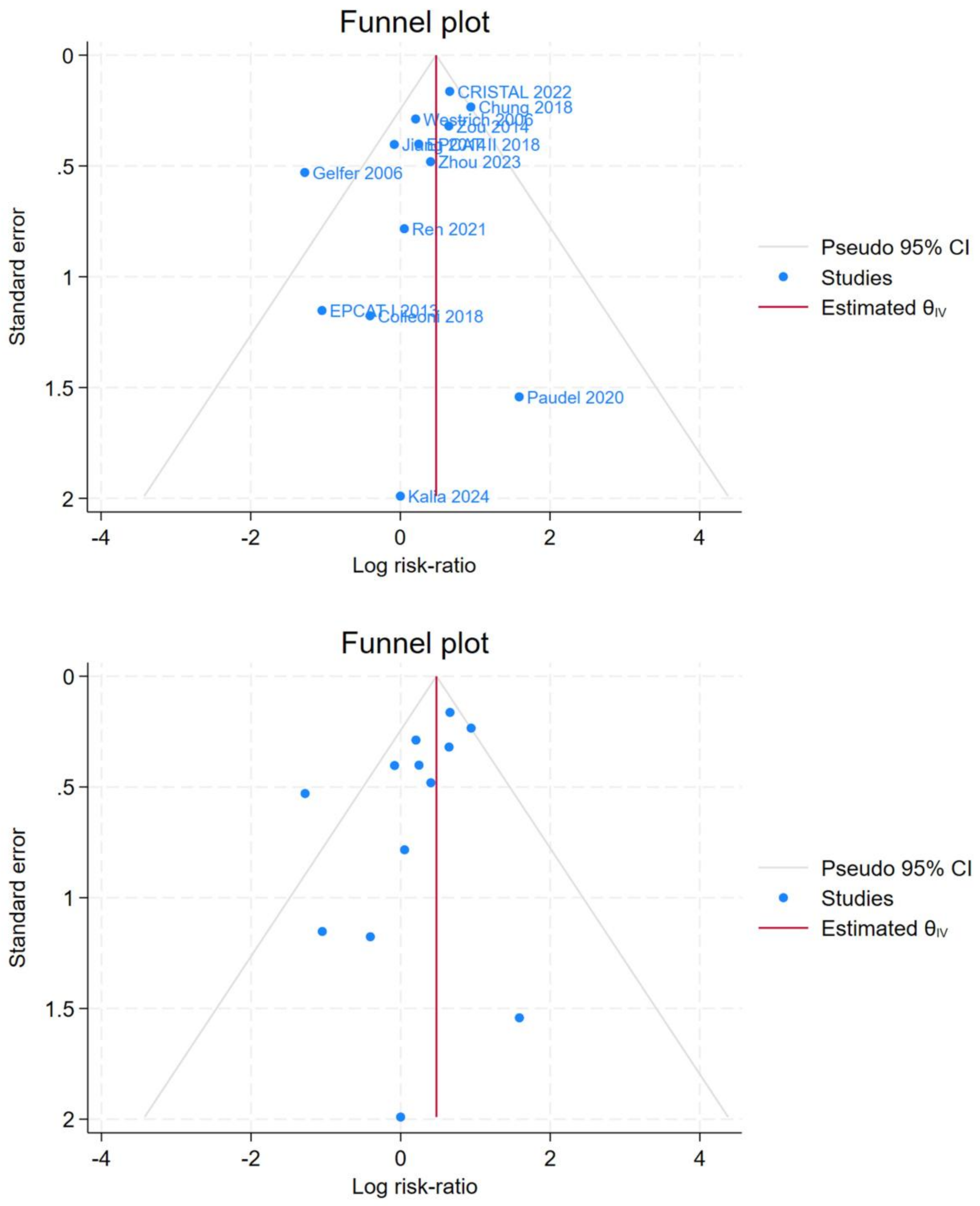

Figure S10. Funnel plot for any DVT. Funnel plot used to assess potential small-study effects. Study references: [1,5,6,19–28].

Figure S11. Forest plot of subgroup analysis of symptomatic DVT by aspirin initiation timing.

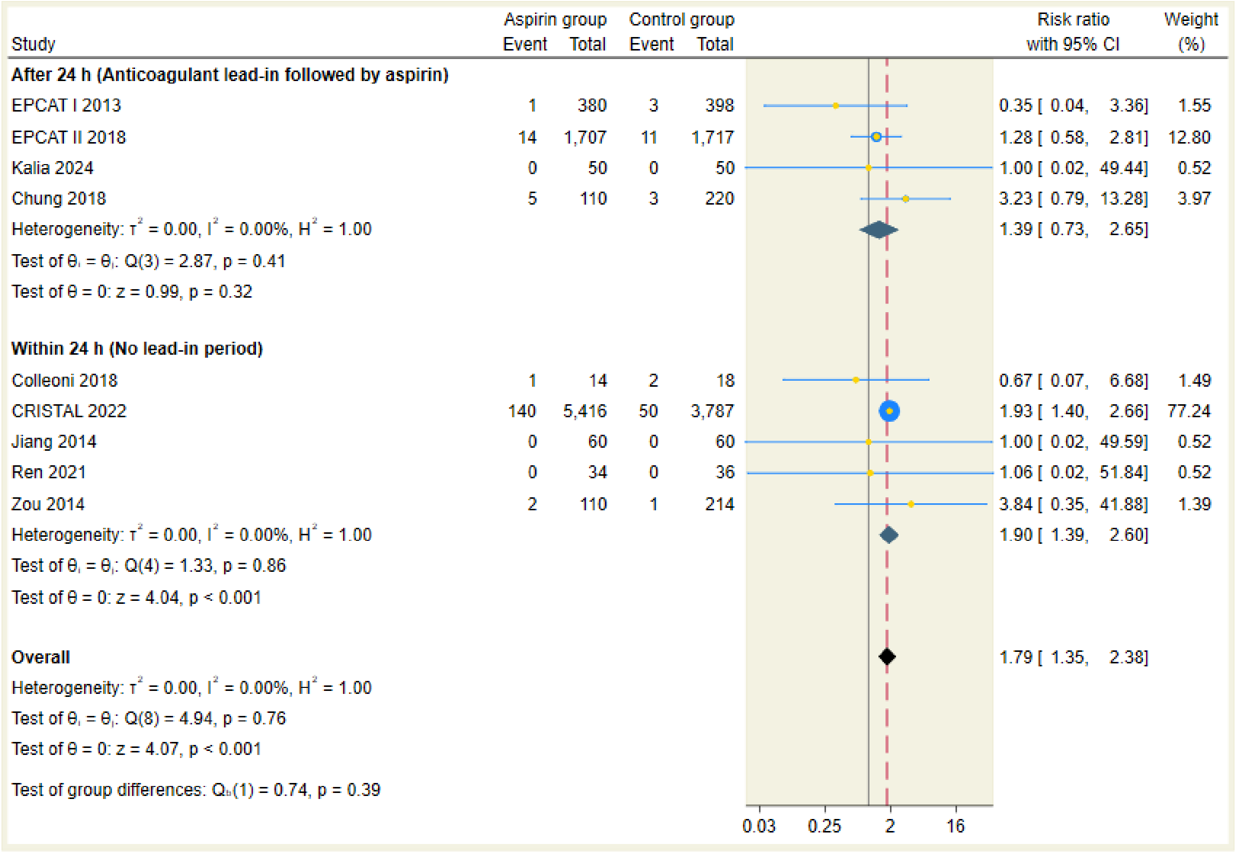

Random-effects DerSimonian–Laird model

Figure S11. Forest plot of subgroup analysis of symptomatic DVT by aspirin initiation timing. Pooled risk ratios were calculated using a random-effects model. CI, confidence interval; DVT, deep vein thrombosis; RR, risk ratio; VTE, venous thromboembolism. Study references: [1,5,6,20,21,23–26].

Figure S12. Forest plot of subgroup analysis of symptomatic DVT by intervention regimen.

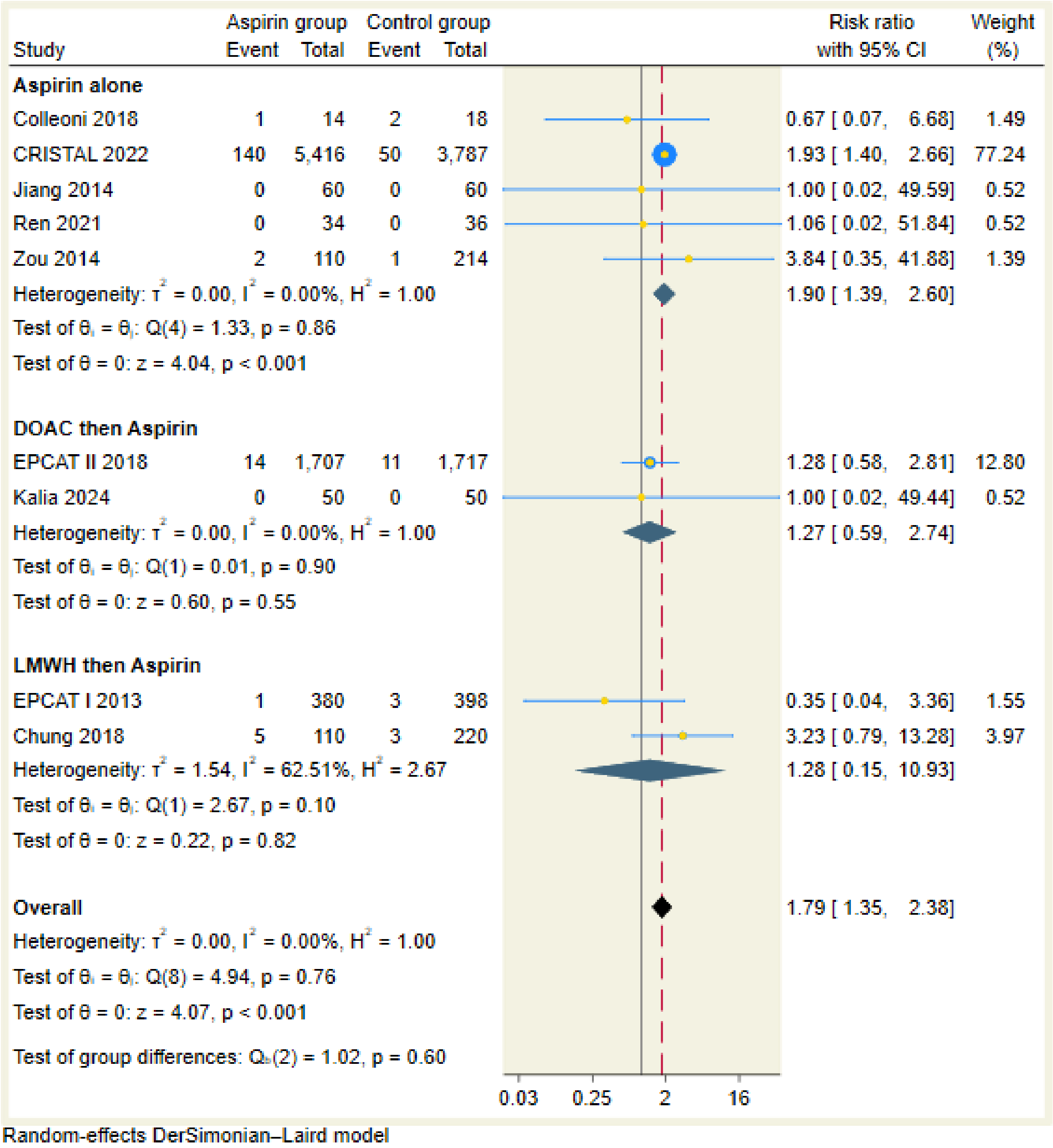

Figure S12. Forest plot of subgroup analysis of symptomatic DVT by intervention regimen. Pooled risk ratios were calculated using a random-effects model. CI, confidence interval; DVT, deep vein thrombosis; RR, risk ratio; VTE, venous thromboembolism. Study references: [1,5,6,20,21,23–26].

Figure S13. Forest plot of subgroup analysis of symptomatic DVT by procedure laterality.

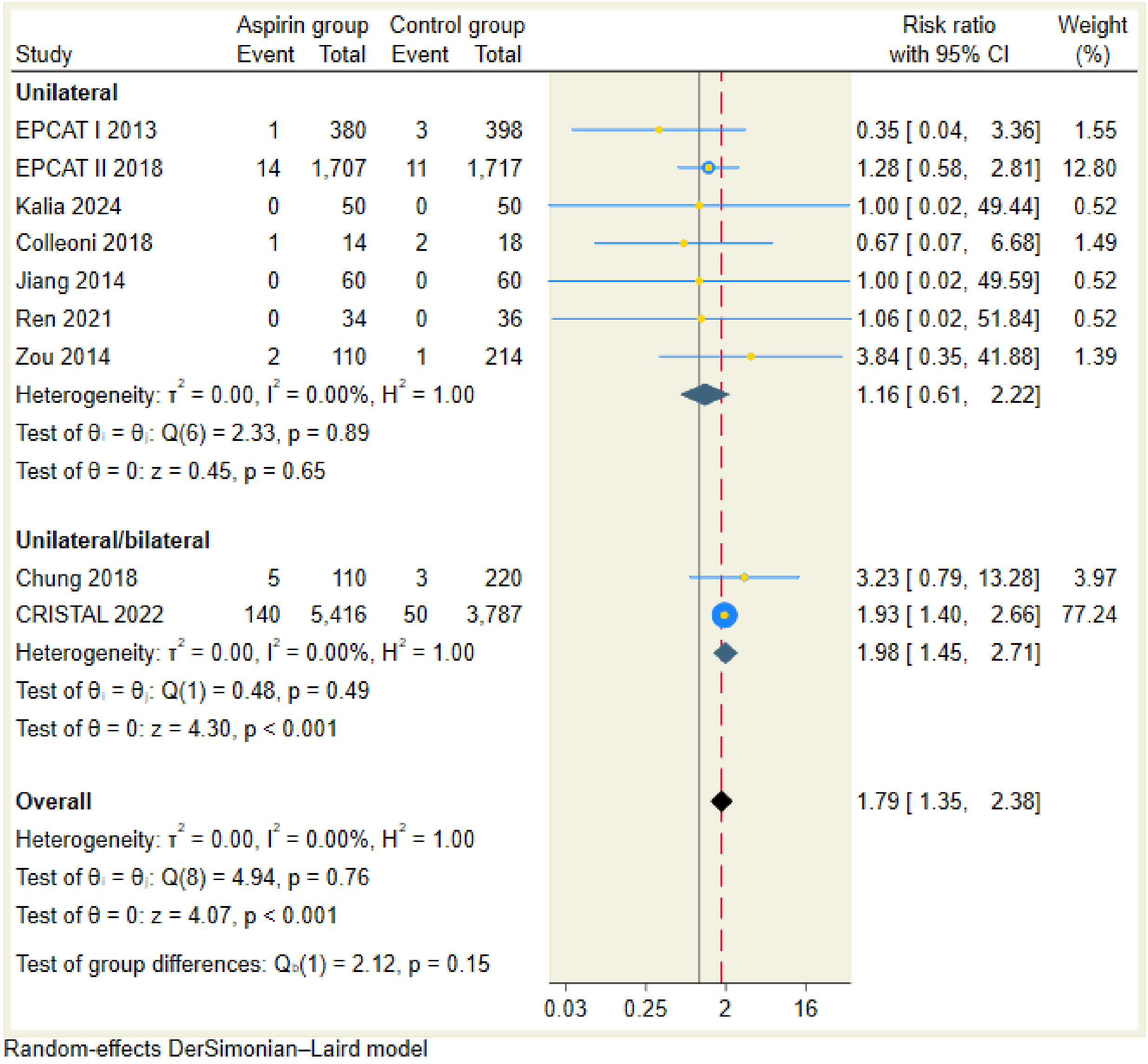

Figure S13. Forest plot of subgroup analysis of symptomatic DVT by procedure laterality. Pooled risk ratios were calculated using a random-effects model. CI, confidence interval; DVT, deep vein thrombosis; RR, risk ratio; VTE, venous thromboembolism. Study references: [1,5,6,20,21,23–26].

Figure S14. Forest plot of subgroup analysis of symptomatic DVT by surgery class.

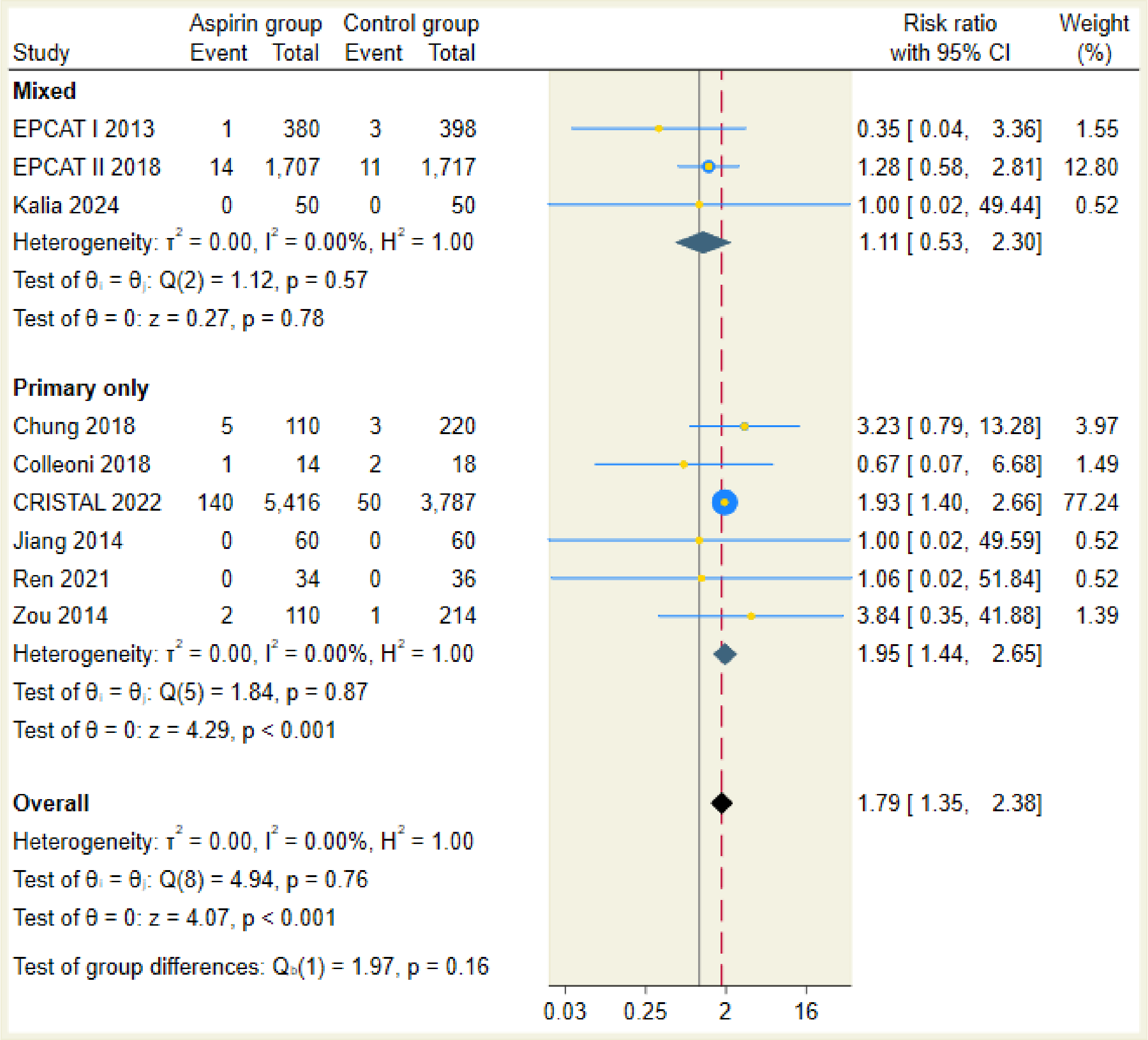

Random-effects DerSimonian–Laird model

Figure S14. Forest plot of subgroup analysis of symptomatic DVT by surgery class. Pooled risk ratios were calculated using a random-effects model. CI, confidence interval; DVT, deep vein thrombosis; RR, risk ratio; VTE, venous thromboembolism. Study references: [1,5,6,20,21,23–26].

Figure S15. Forest plot of subgroup analysis of symptomatic DVT by surgery type.

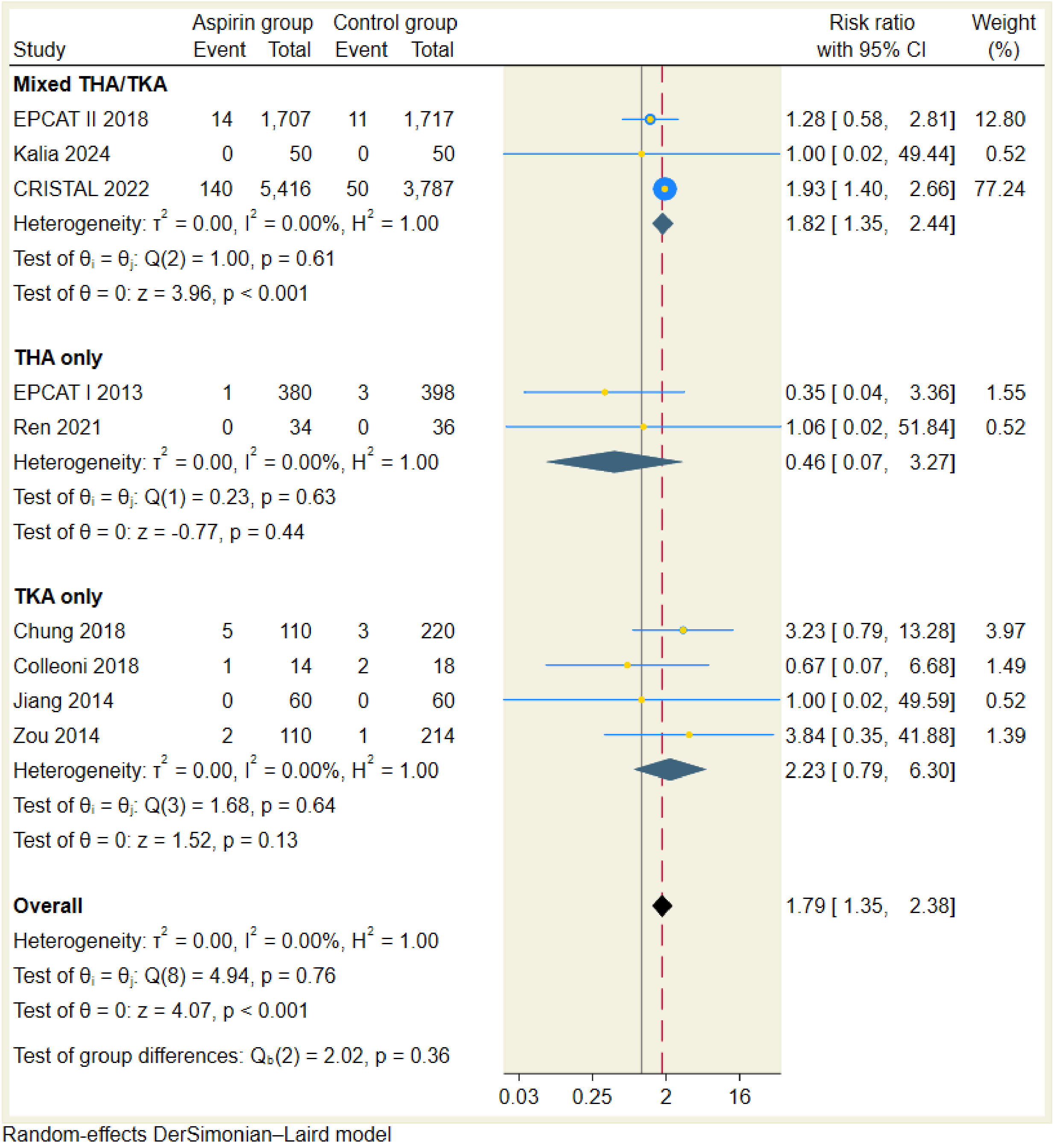

Figure S15. Forest plot of subgroup analysis of symptomatic DVT by surgery type. Pooled risk ratios were calculated using a random-effects model. CI, confidence interval; DVT, deep vein thrombosis; RR, risk ratio; VTE, venous thromboembolism. Study references: [1,5,6,20,21,23–26].

Figure S16. Forest plot of subgroup analysis of symptomatic DVT by total daily aspirin dose.

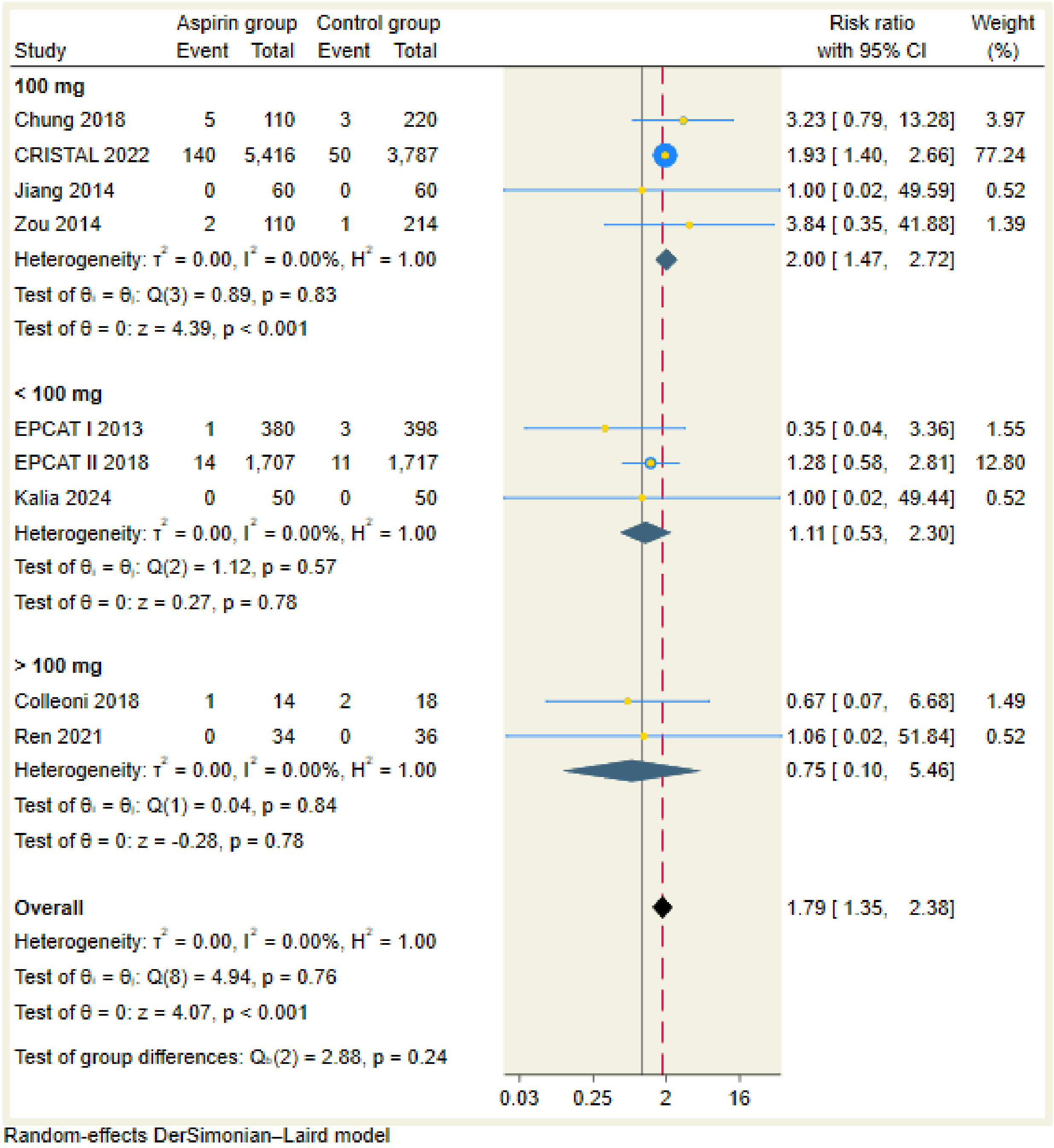

Figure S16. Forest plot of subgroup analysis of symptomatic DVT by total daily aspirin dose. Pooled risk ratios were calculated using a random-effects model. CI, confidence interval; DVT, deep vein thrombosis; RR, risk ratio; VTE, venous thromboembolism. Study references: [1,5,6,20,21,23–26].

Figure S17. Forest plot of subgroup analysis of symptomatic DVT by follow-up duration.

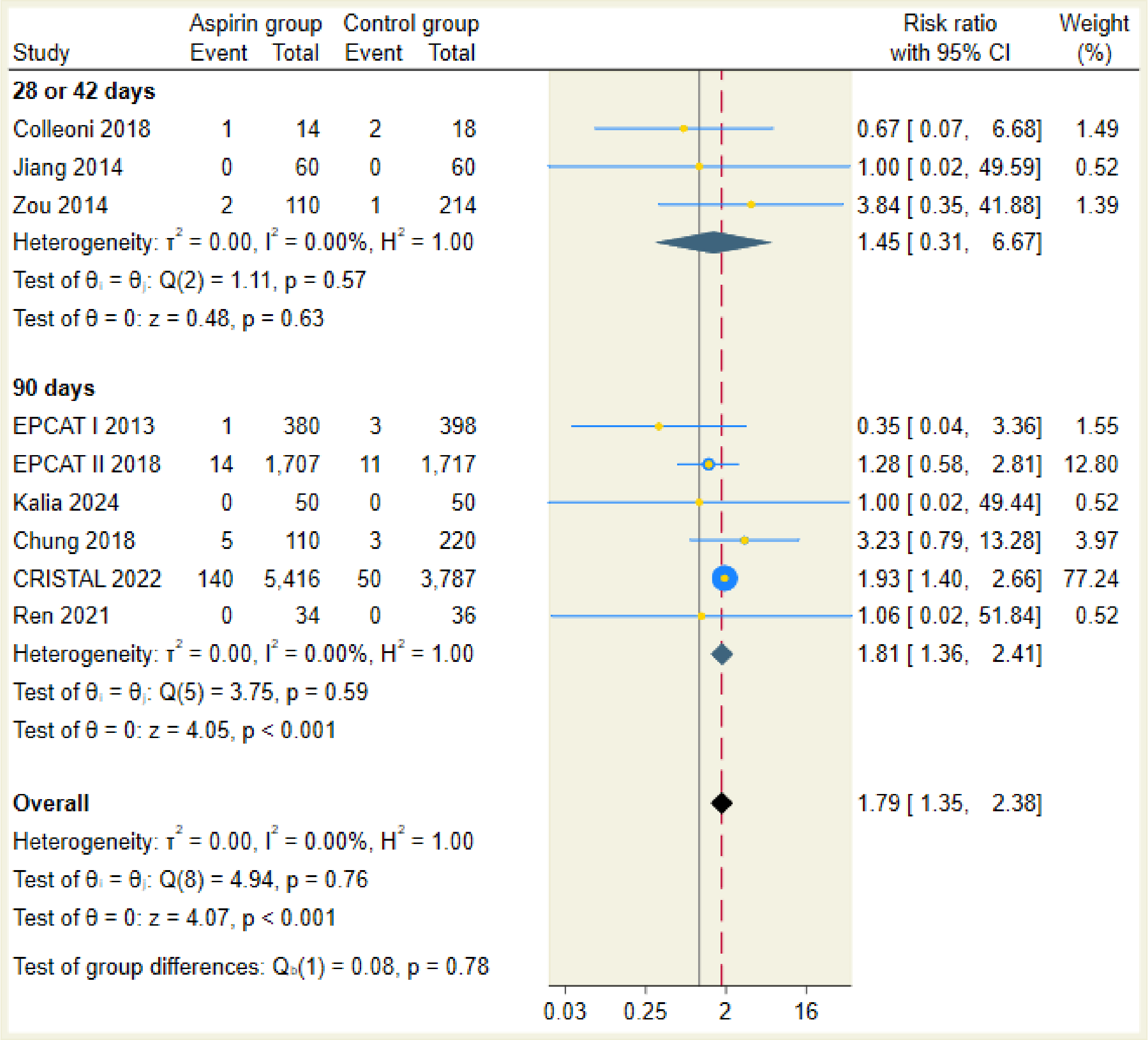

Random-effects DerSimonian–Laird model

Figure S17. Forest plot of subgroup analysis of symptomatic DVT by follow-up duration. Pooled risk ratios were calculated using a random-effects model. CI, confidence interval; DVT, deep vein thrombosis; RR, risk ratio; VTE, venous thromboembolism. Study references: [1,5,6,20,21,23–26].

Figure S18. Leave-one-out analysis of symptomatic DVT.

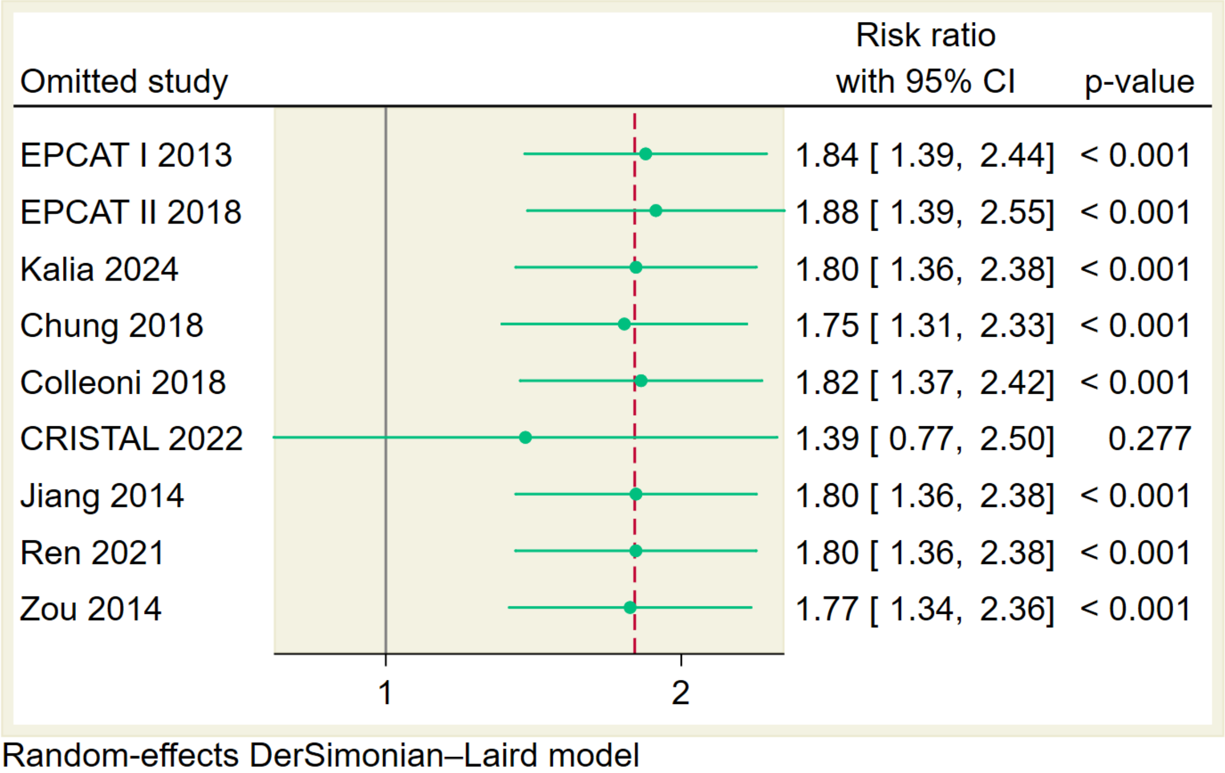

Figure S18. Leave-one-out analysis of symptomatic DVT. The pooled risk ratio was recalculated after omitting one study at a time. Study references: [1,5,6,20,21,23–26].

Figure S19. Galbraith plot for symptomatic DVT.

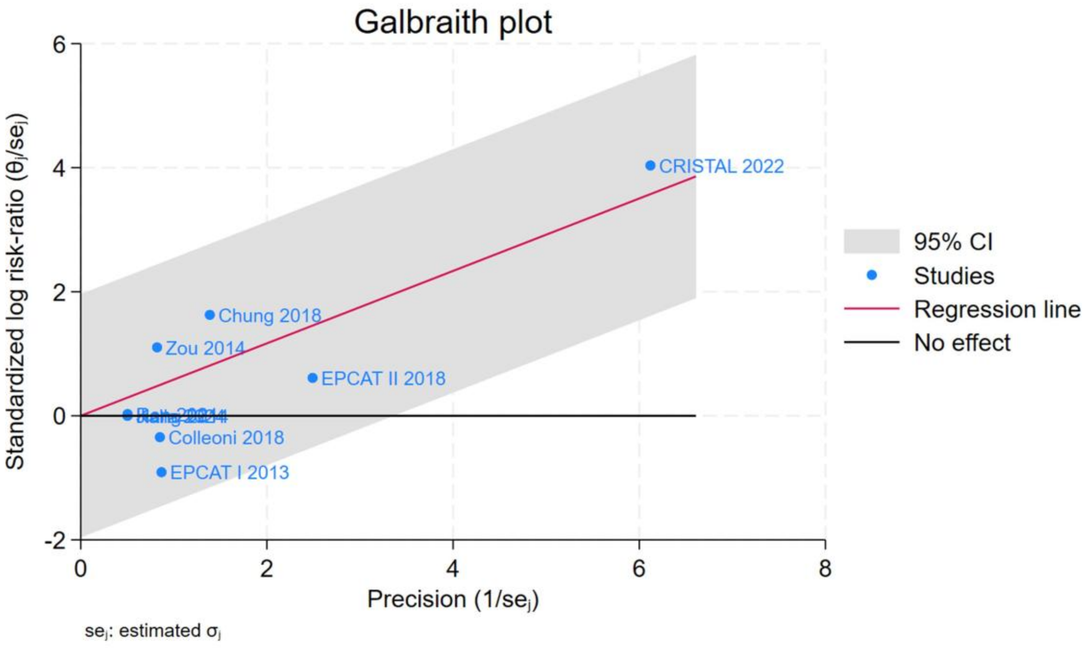

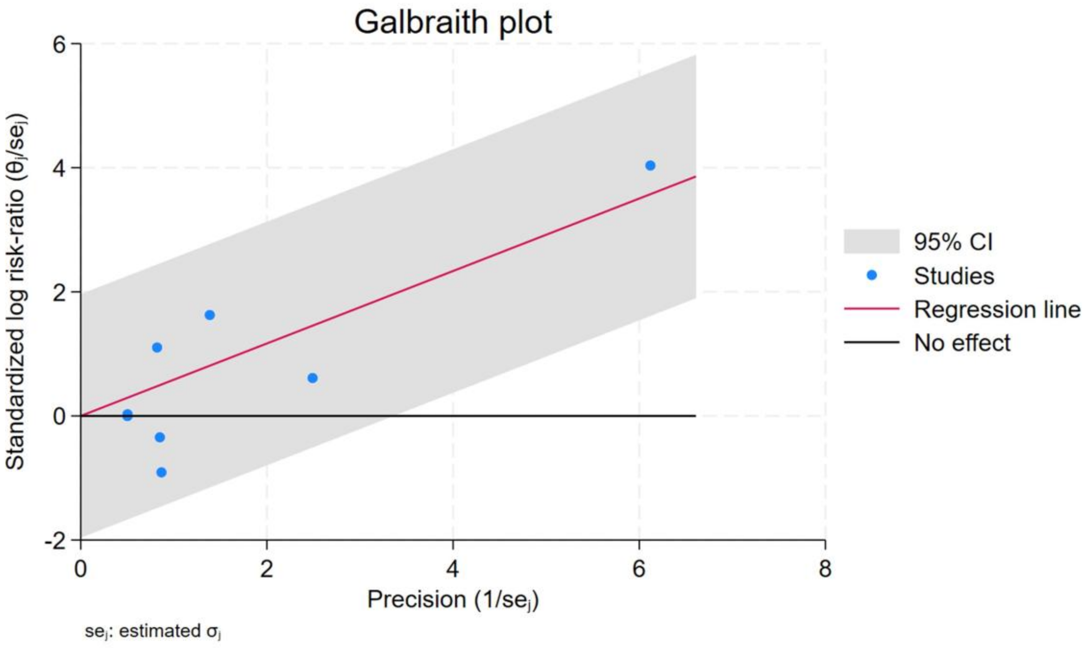

Figure S19. Galbraith plot for symptomatic DVT. Radial plot used to explore potential outlying or influential studies. Study references: [1,5,6,20,21,23–26].

Figure S20. Doi plot and LFK index for symptomatic DVT.

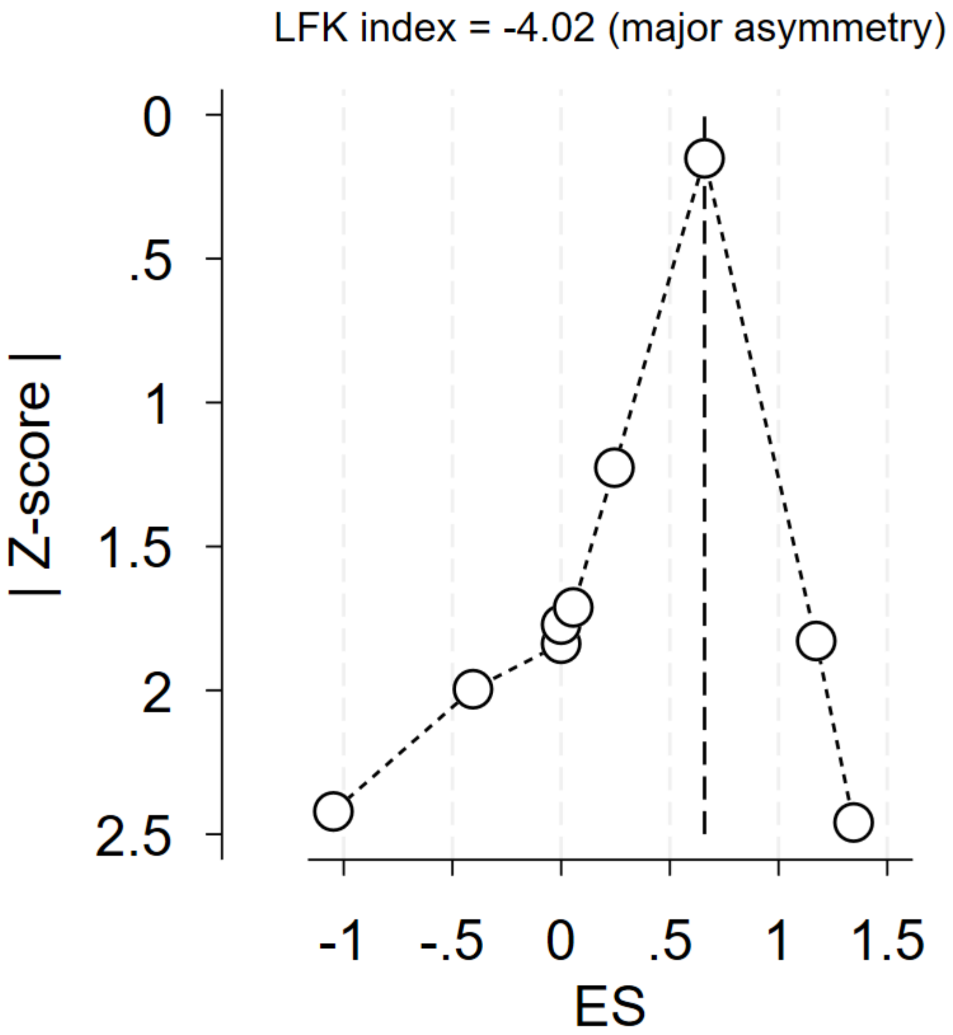

Figure S20. Doi plot and LFK index for symptomatic DVT. Doi plot with the Luis Furuya-Kanamori (LFK) index. Study references [1,5,6,20,21,23–26].

Figure S21. Forest plot of subgroup analysis of symptomatic VTE by aspirin initiation timing.

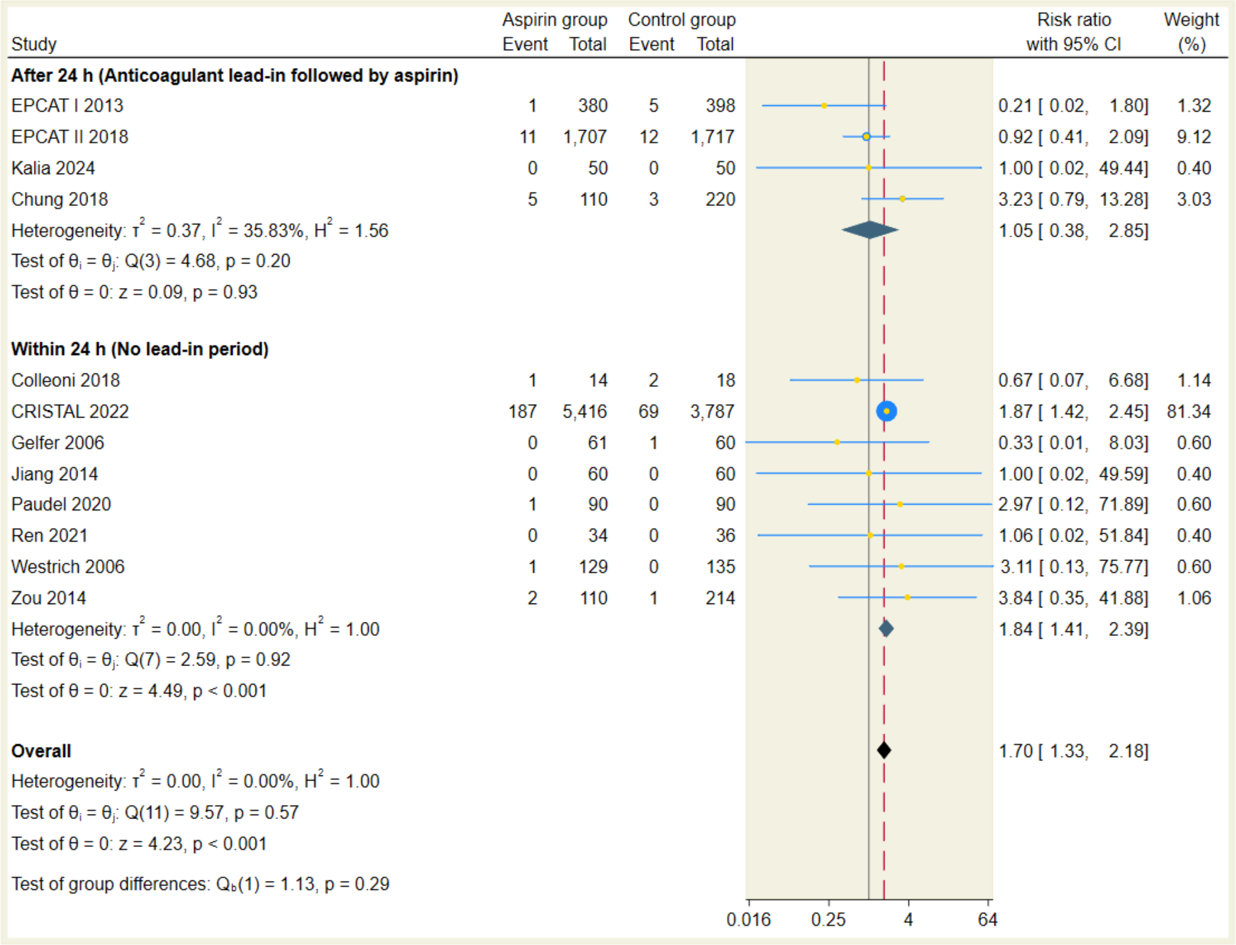

Random-effects DerSimonian–Laird model

Figure S21. Forest plot of subgroup analysis of symptomatic VTE by aspirin initiation timing. Pooled risk ratios were calculated using a random-effects model. CI, confidence interval; DVT, deep vein thrombosis; RR, risk ratio; VTE, venous thromboembolism. Study references: [1,5,6,20–28].

Figure S22. Forest plot of subgroup analysis of symptomatic VTE by intervention regimen.

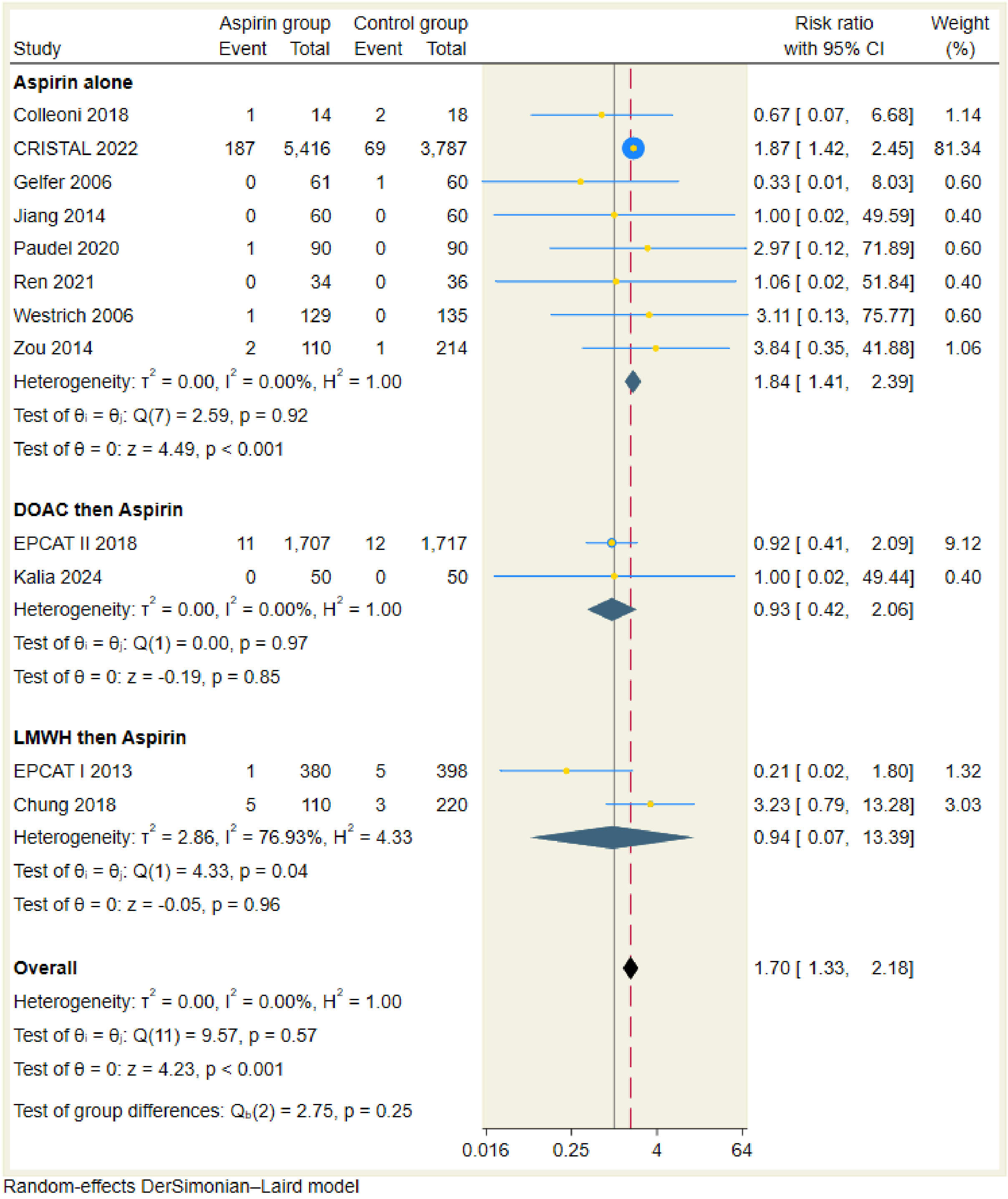

Figure S22. Forest plot of subgroup analysis of symptomatic VTE by intervention regimen. Pooled risk ratios were calculated using a random-effects model. CI, confidence interval; DVT, deep vein thrombosis; RR, risk ratio; VTE, venous thromboembolism. Study references: [1,5,6,20–28].

Figure S23. Forest plot of subgroup analysis of symptomatic VTE by procedure laterality.

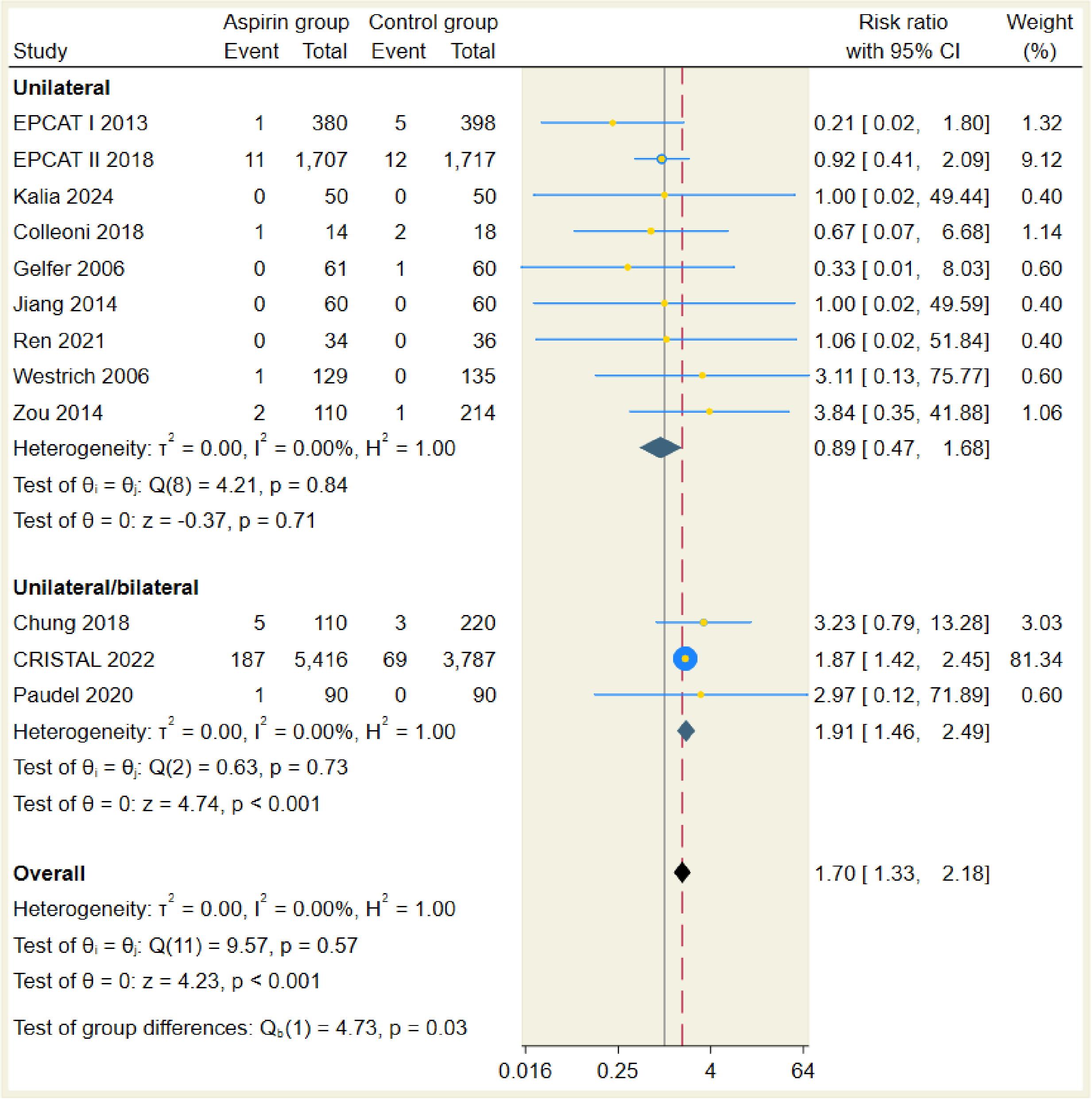

Random-effects DerSimonian–Laird model

Figure S23. Forest plot of subgroup analysis of symptomatic VTE by procedure laterality. Pooled risk ratios were calculated using a random-effects model. CI, confidence interval; DVT, deep vein thrombosis; RR, risk ratio; VTE, venous thromboembolism. Study references: [1,5,6,20–28].

Figure S24. Forest plot of subgroup analysis of symptomatic VTE by surgery class.

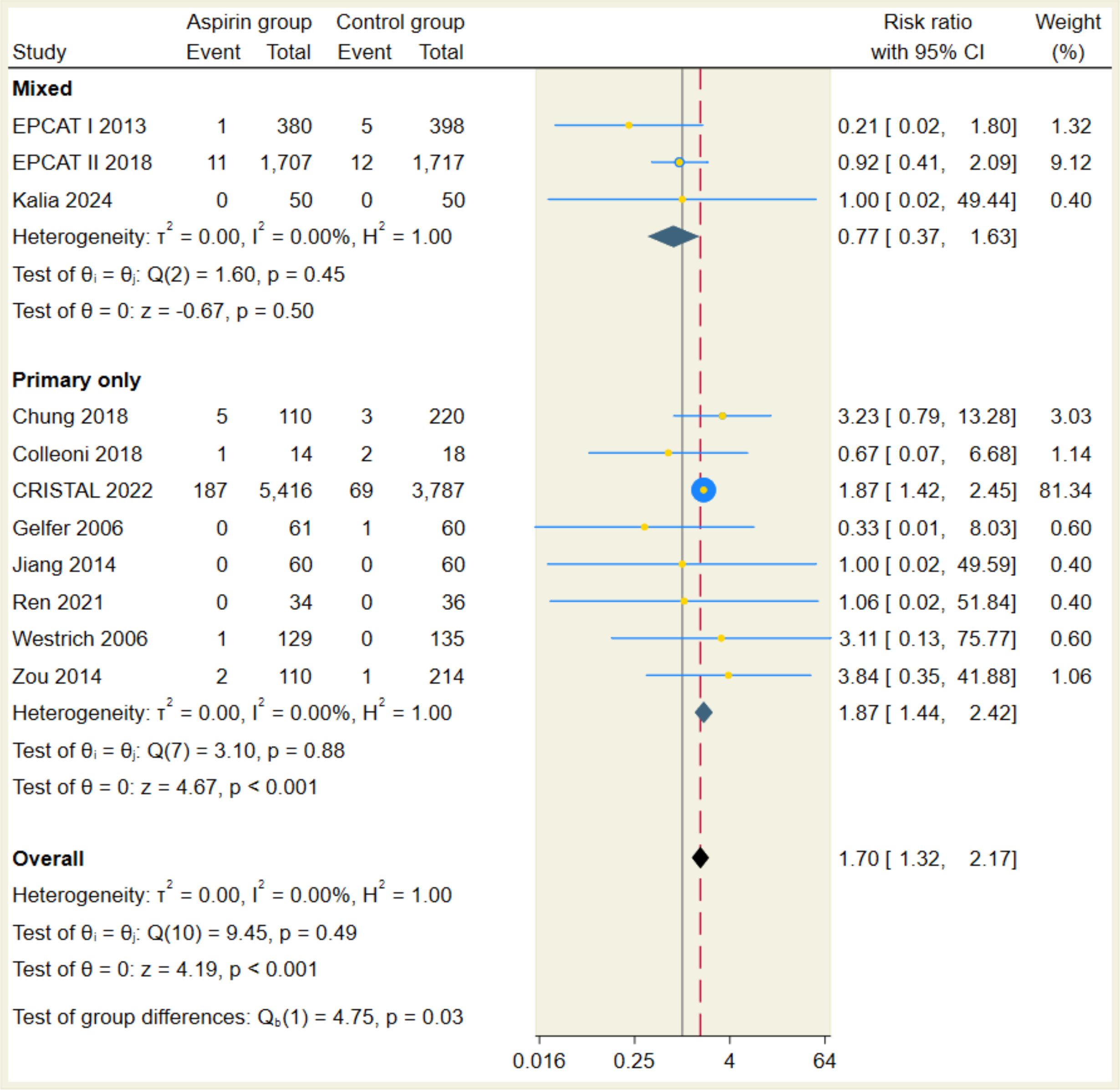

Random-effects DerSimonian–Laird model

Figure S24. Forest plot of subgroup analysis of symptomatic VTE by surgery class. Pooled risk ratios were calculated using a random-effects model. CI, confidence interval; DVT, deep vein thrombosis; RR, risk ratio; VTE, venous thromboembolism. Study references: [1,5,6,20–28].

Figure S25. Forest plot of subgroup analysis of symptomatic VTE by surgery type.

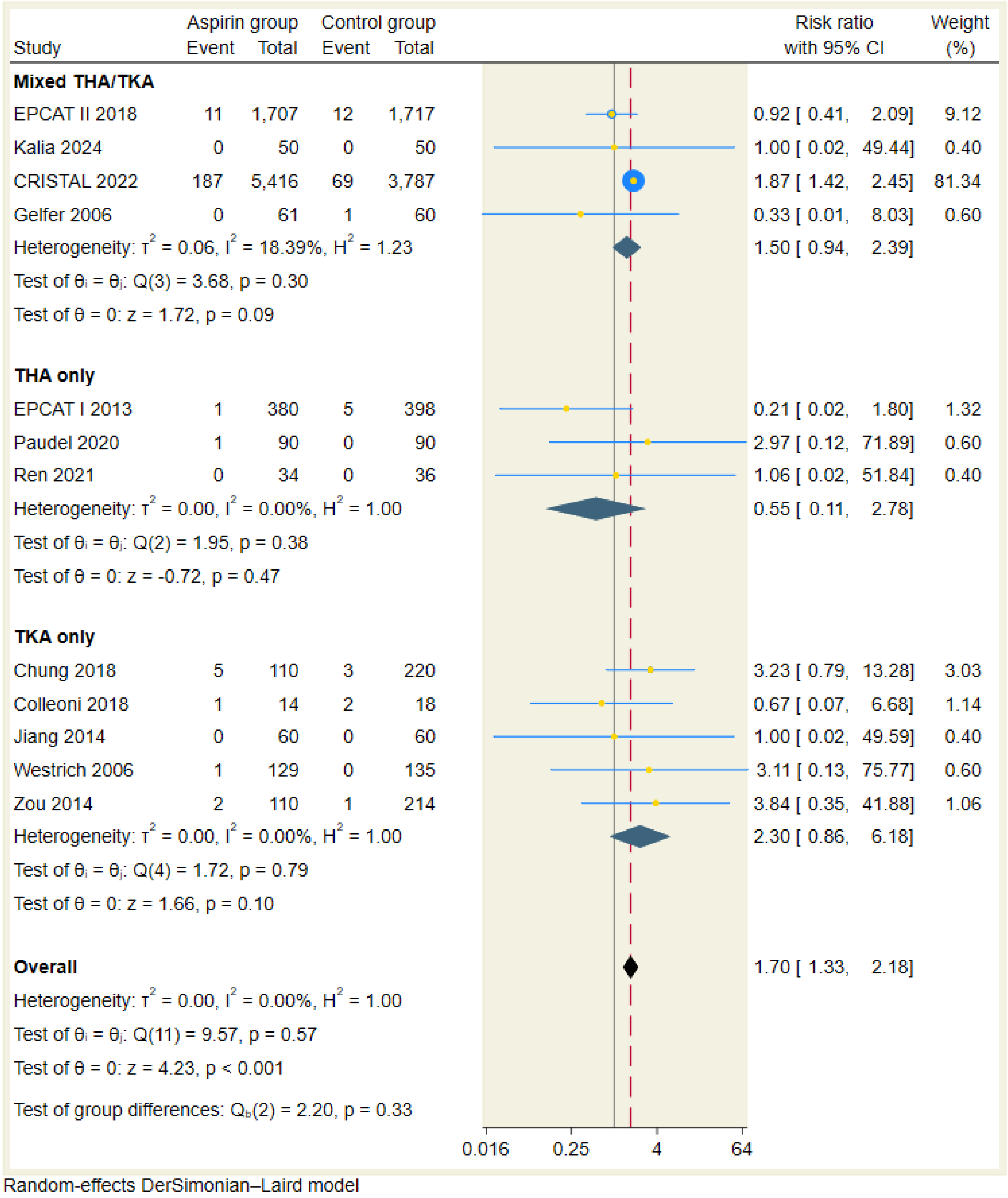

Figure S25. Forest plot of subgroup analysis of symptomatic VTE by surgery type. Pooled risk ratios were calculated using a random-effects model. CI, confidence interval; DVT, deep vein thrombosis; RR, risk ratio; VTE, venous thromboembolism. Study references: [1,5,6,20–28].

Figure S26. Forest plot of subgroup analysis of symptomatic VTE by total daily aspirin dose.

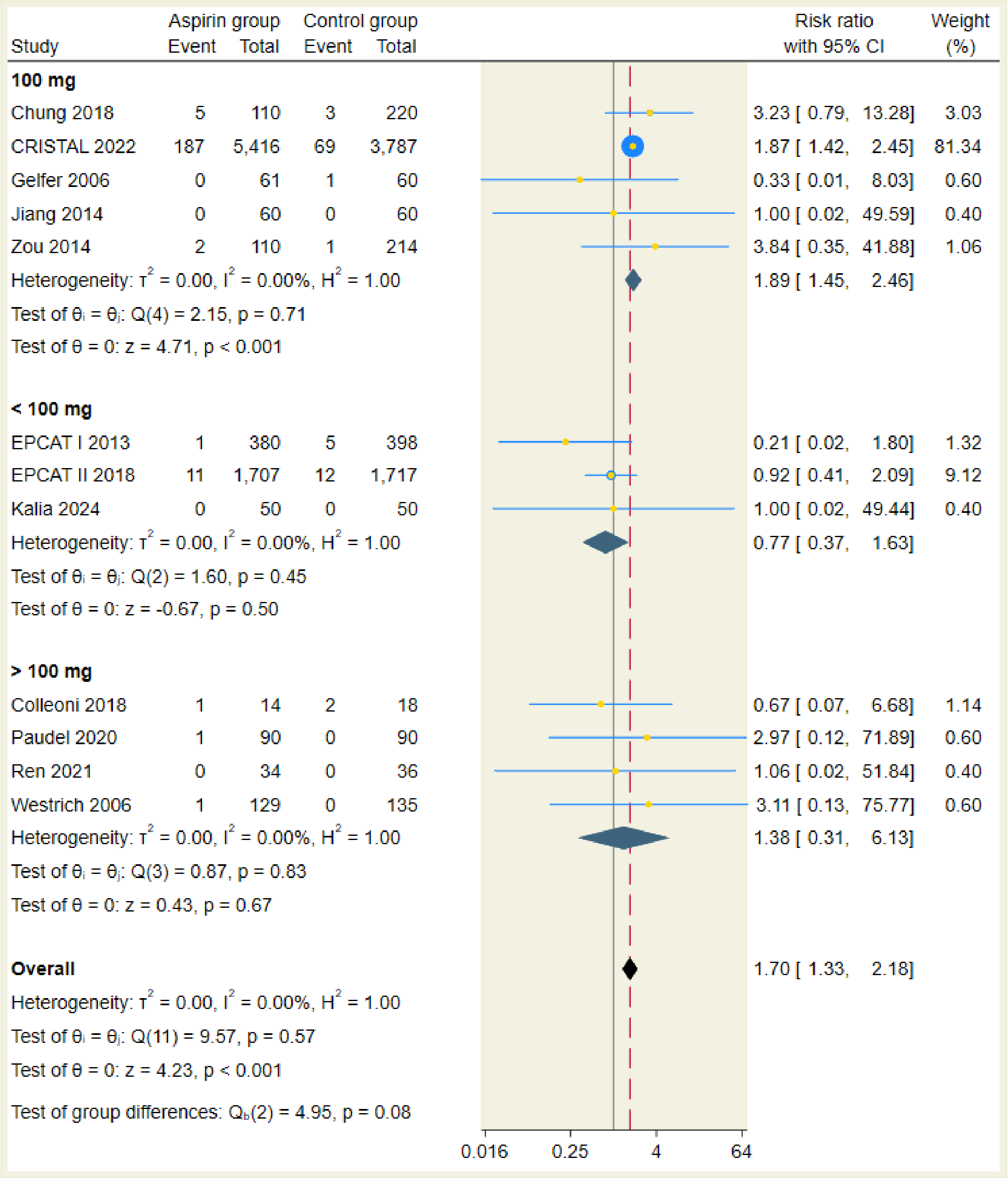

Random-effects DerSimonian–Laird model

Figure S26. Forest plot of subgroup analysis of symptomatic VTE by total daily aspirin dose. Pooled risk ratios were calculated using a random-effects model. CI, confidence interval; DVT, deep vein thrombosis; RR, risk ratio; VTE, venous thromboembolism. Study references: [1,5,6,20–28].

Figure S27. Forest plot of subgroup analysis of symptomatic VTE by follow-up duration.

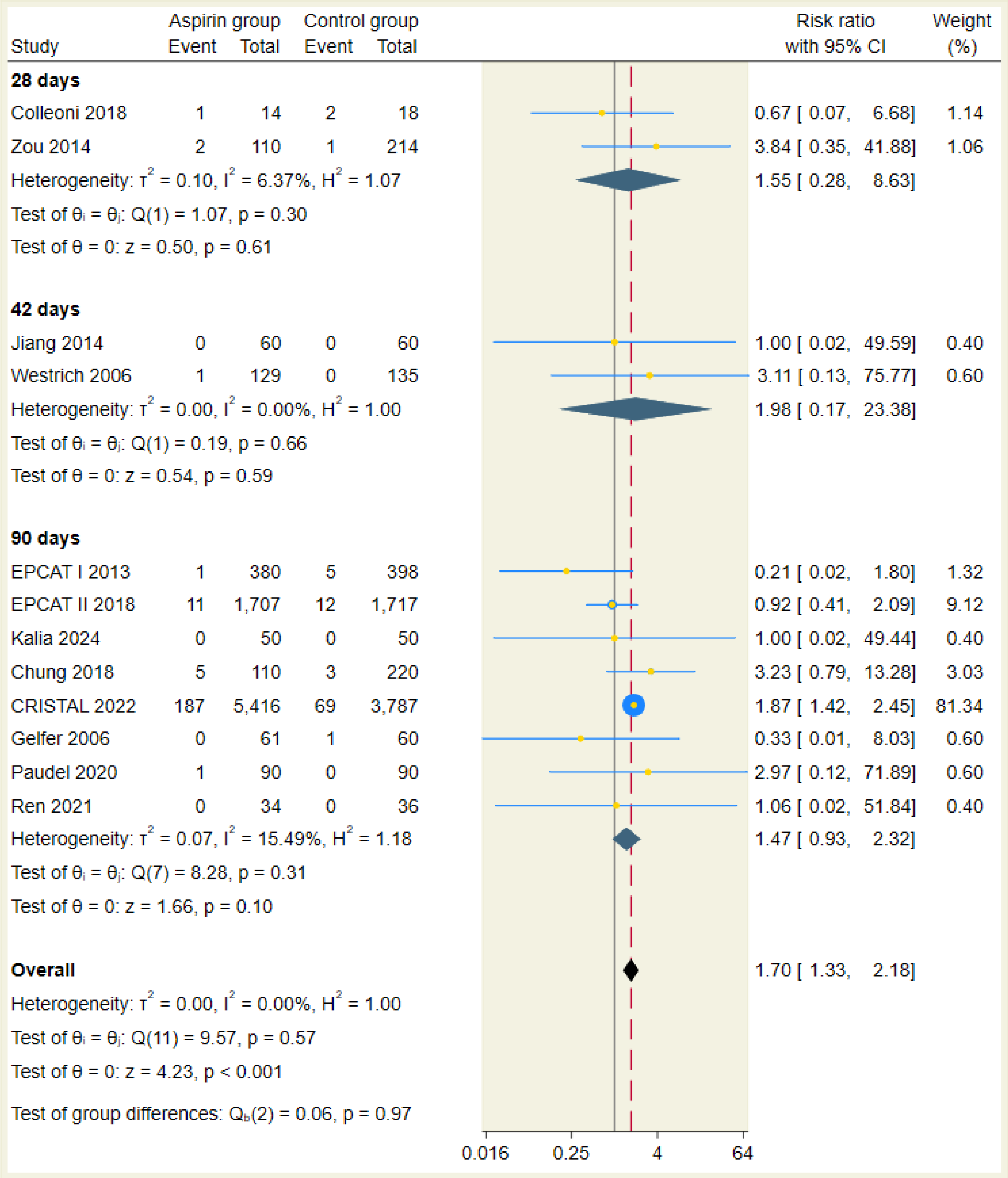

Random-effects DerSimonian–Laird model

Figure S27. Forest plot of subgroup analysis of symptomatic VTE by follow-up duration. Pooled risk ratios were calculated using a random-effects model. CI, confidence interval; DVT, deep vein thrombosis; RR, risk ratio; VTE, venous thromboembolism. Study references: [1,5,6,20–28].

Figure S28. Leave-one-out analysis of symptomatic VTE.

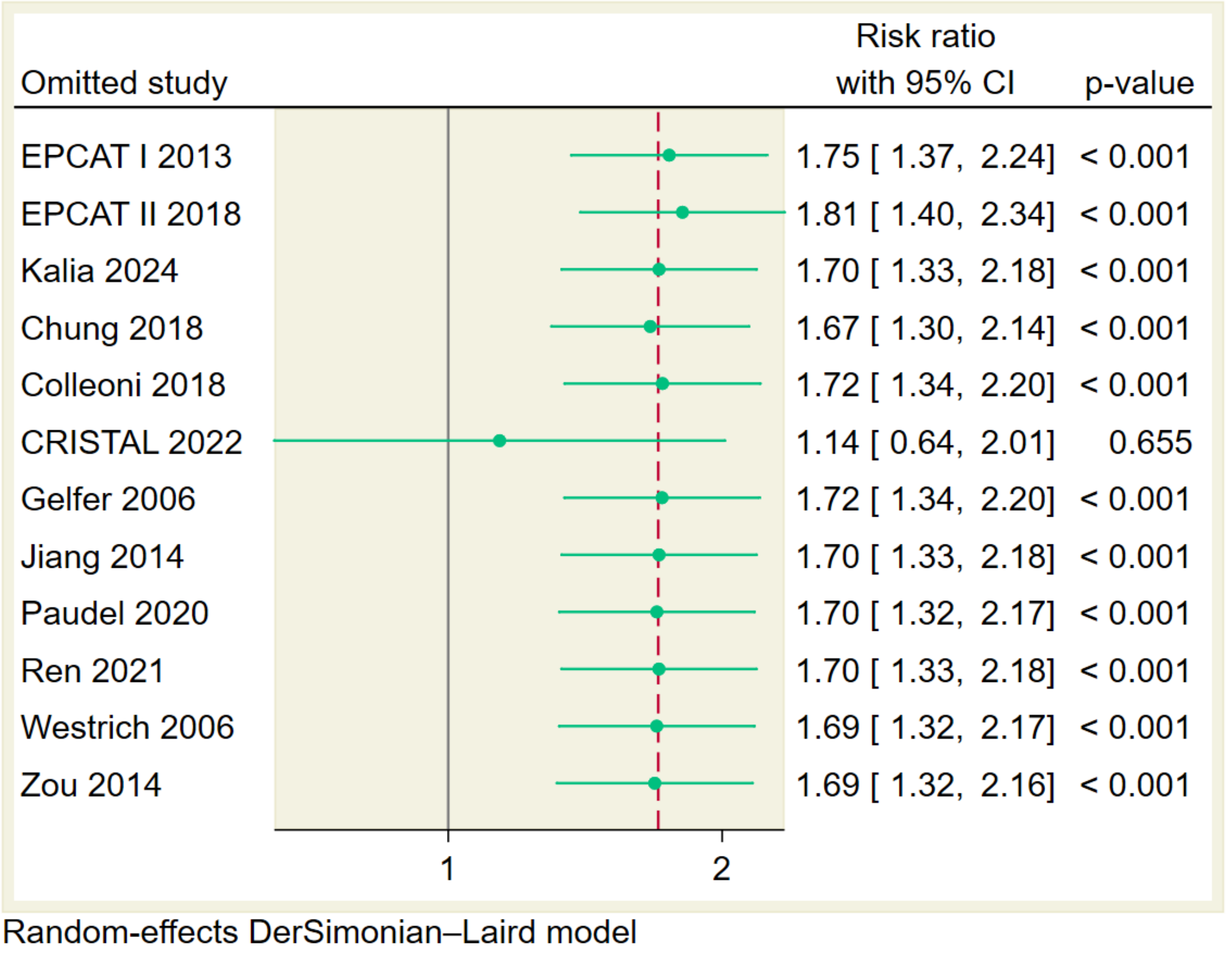

Figure S28. Leave-one-out analysis of symptomatic VTE. The pooled risk ratio was recalculated after omitting one study at a time. Study references: [1,5,6,20–28].

Figure S29. Galbraith plot for symptomatic VTE.

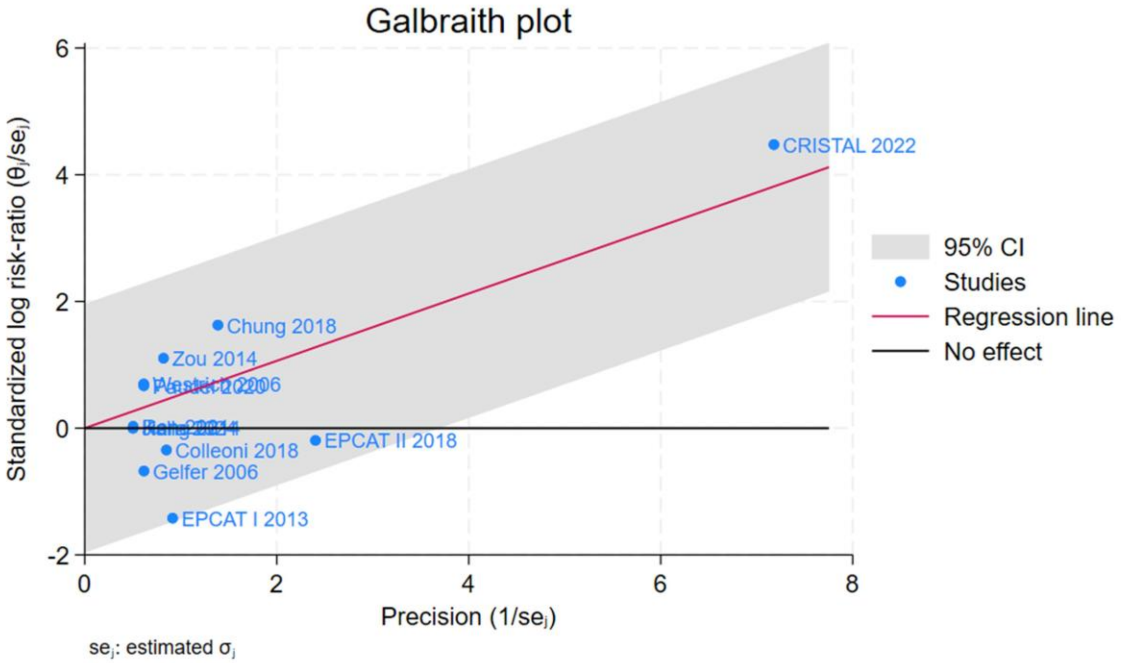

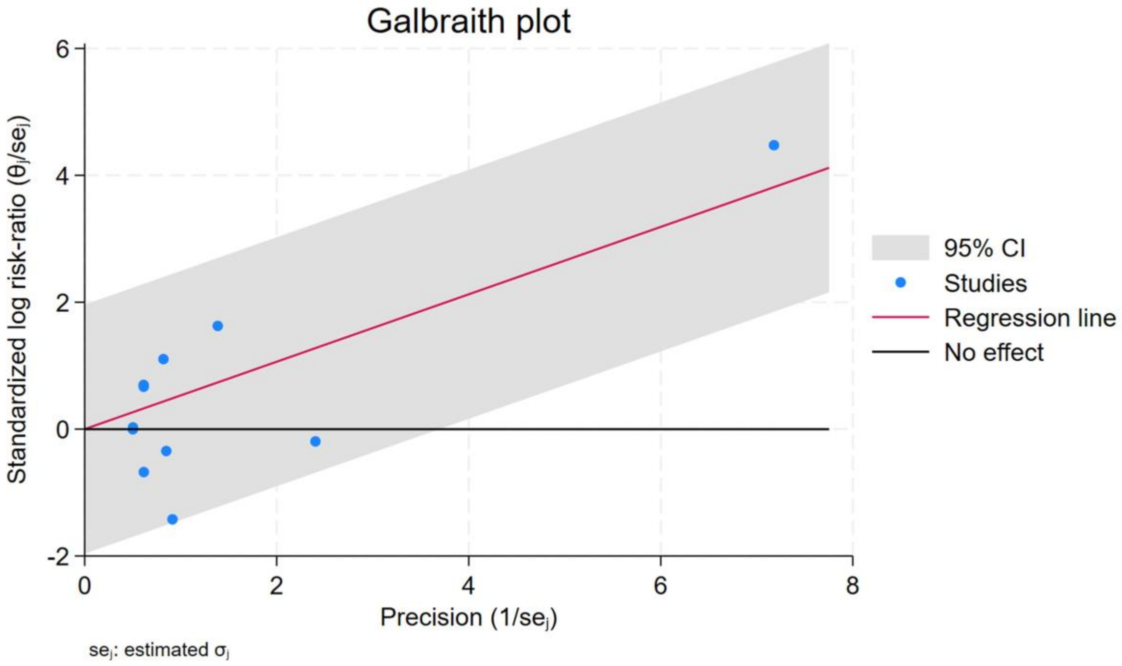

Figure S29. Galbraith plot for symptomatic VTE. Radial plot used to explore potential outlying or influential studies. Study references: [1,5,6,20–28].

Figure S30. Funnel plot for symptomatic VTE.

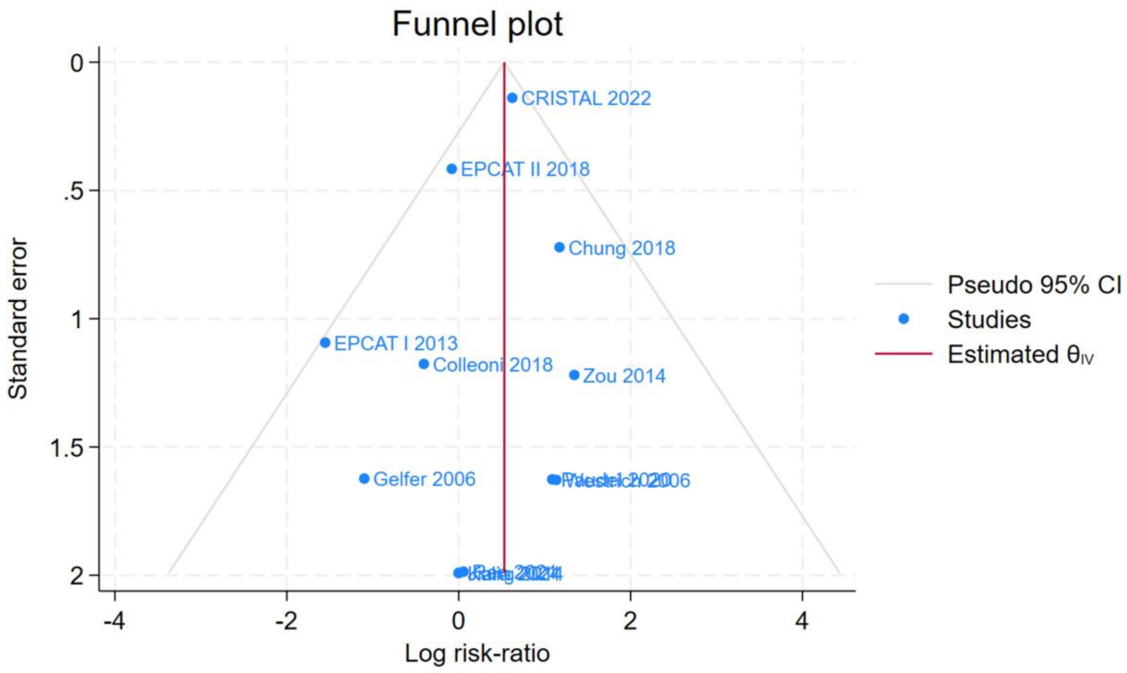

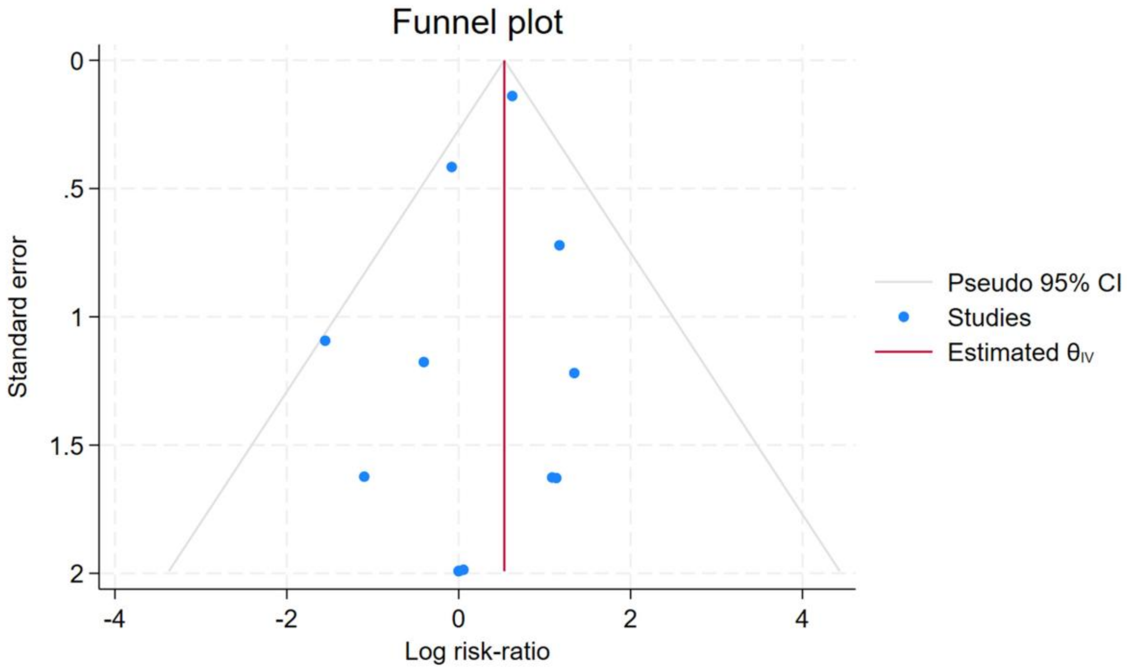

Figure S30. Funnel plot for symptomatic VTE. Funnel plot used to assess potential small-study effects. Study references: [1,5,6,20–28].

Figure S31. Forest plot of proximal deep vein thrombosis.

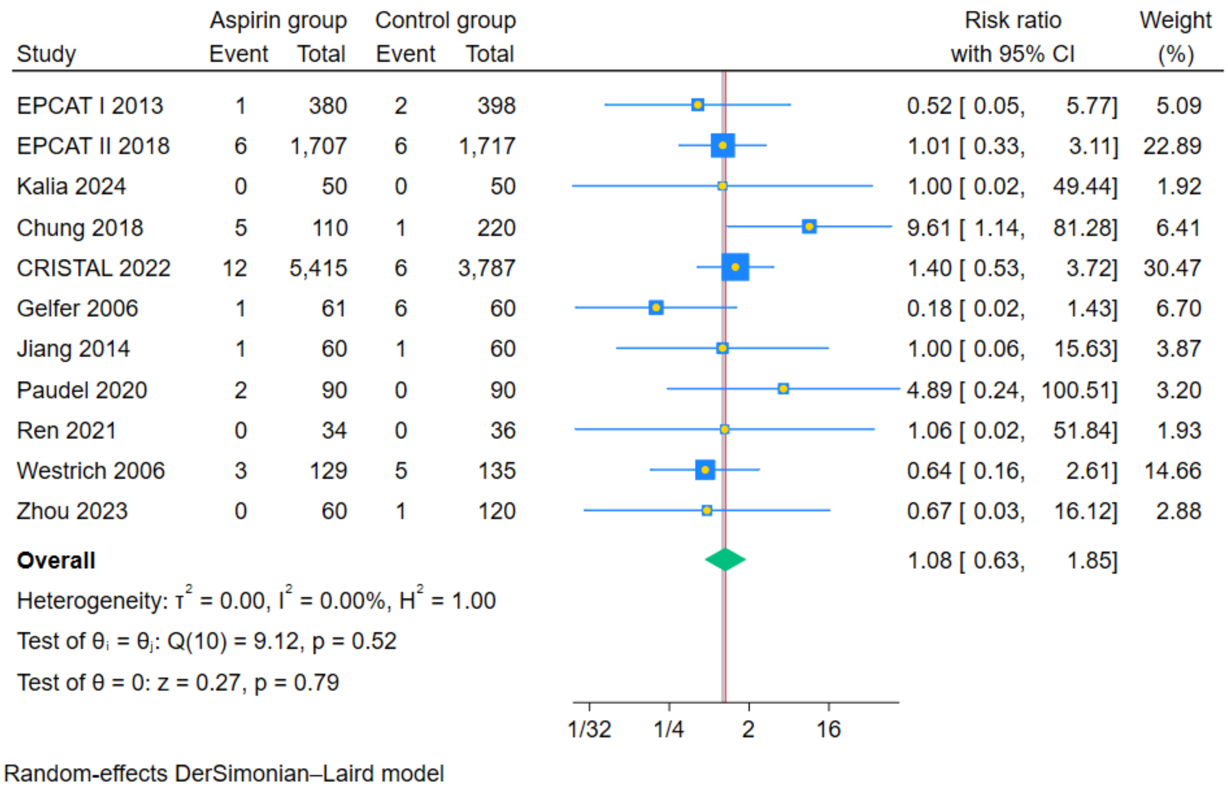

Figure S31. Forest plot of proximal deep vein thrombosis. Pooled risk ratios were calculated using a random-effects model. CI, confidence interval; DVT, deep vein thrombosis; RR, risk ratio; VTE, venous thromboembolism. Study references: [1,5,6,19–23,25,27,28].

Figure S32. Forest plot of distal deep vein thrombosis.

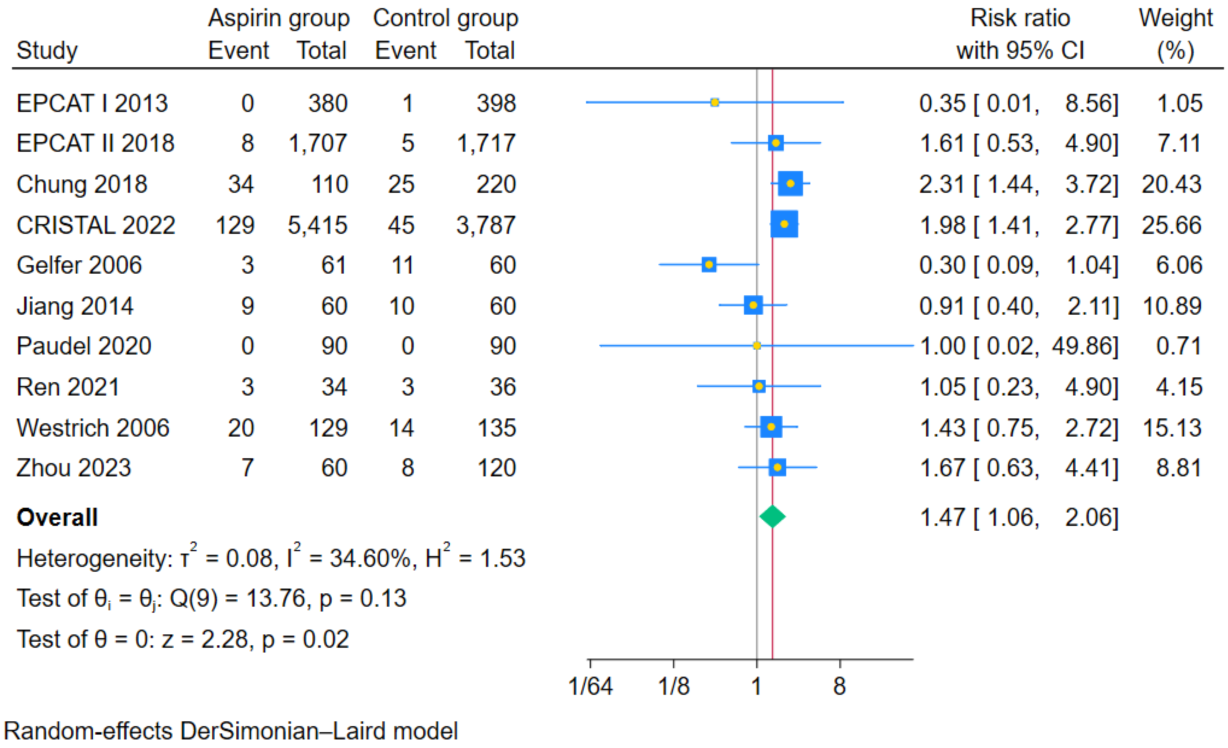

Figure S32. Forest plot of distal deep vein thrombosis. Pooled risk ratios were calculated using a random-effects model. CI, confidence interval; DVT, deep vein thrombosis; RR, risk ratio; VTE, venous thromboembolism. Study references: [1,5,6,19,21–23,25,27,28].

Figure S33. Forest plot of major bleeding.

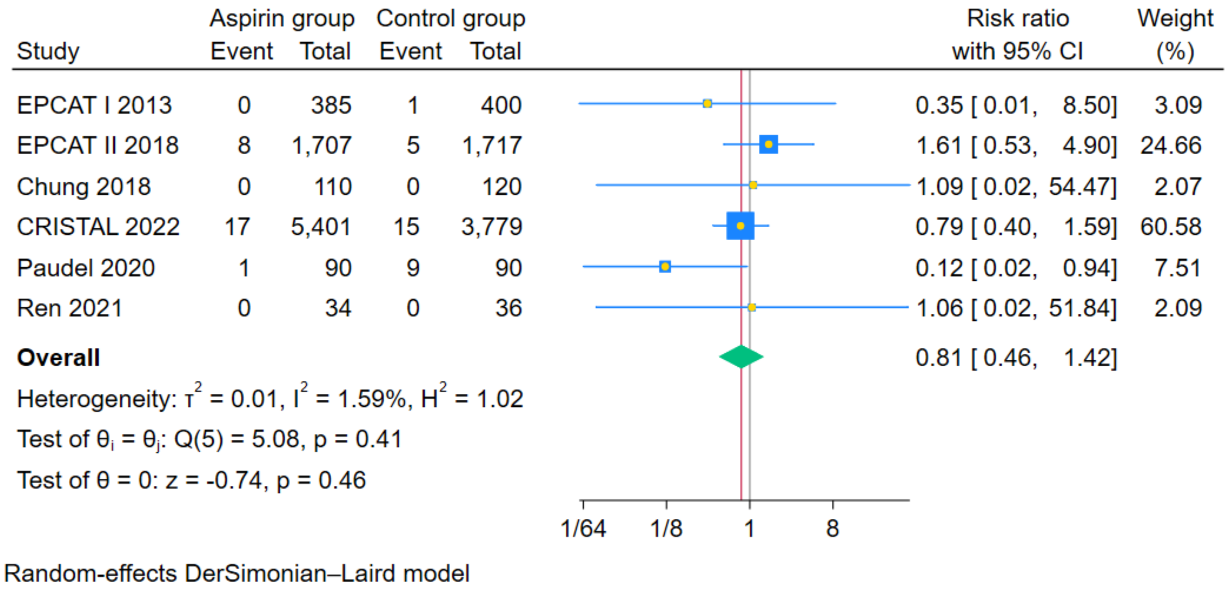

Figure S33. Forest plot of major bleeding. Pooled risk ratios were calculated using a random-effects model. CI, confidence interval; DVT, deep vein thrombosis; RR, risk ratio; VTE, venous thromboembolism. Study references: [1,5,6,21–23].

Figure S34. Forest plot of clinically relevant non-major bleeding.

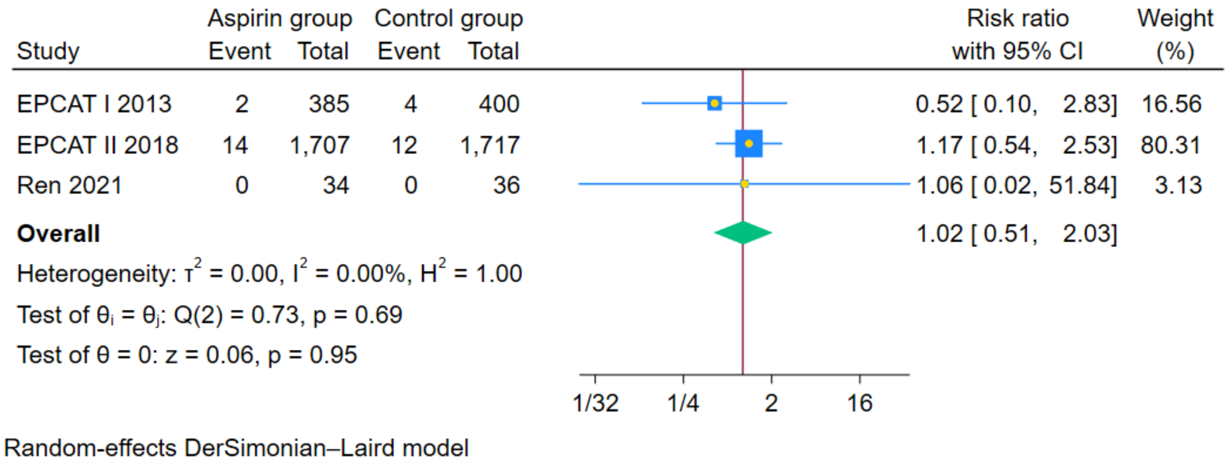

Figure S34. Forest plot of clinically relevant non-major bleeding. Pooled risk ratios were calculated using a random-effects model. CI, confidence interval; DVT, deep vein thrombosis; RR, risk ratio; VTE, venous thromboembolism. Study references: [5,6,21].

Figure S35. Forest plot of minor bleeding.

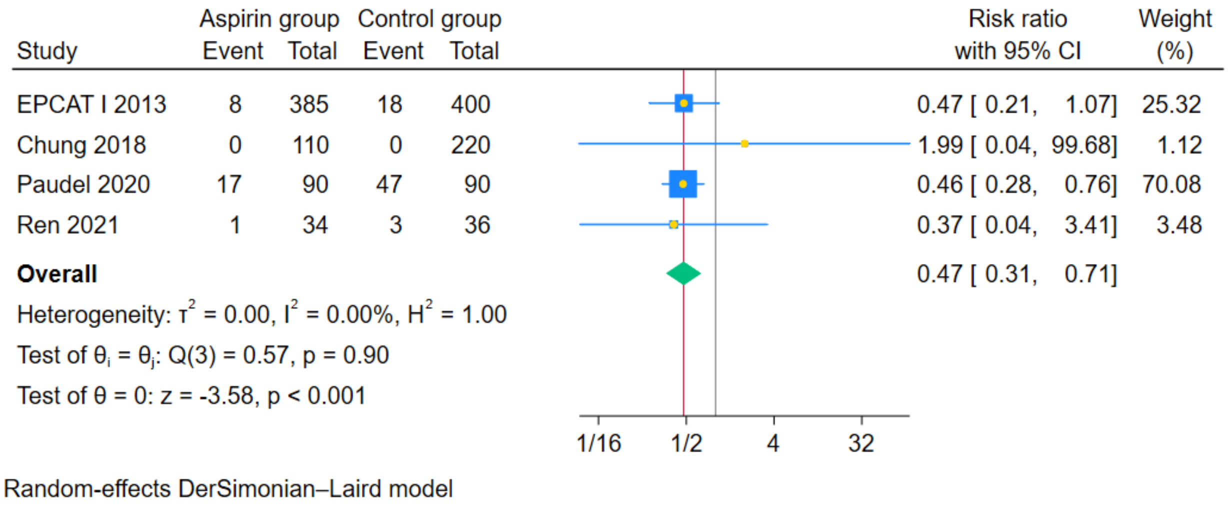

Figure S35. Forest plot of minor bleeding. Pooled risk ratios were calculated using a random-effects model. CI, confidence interval; DVT, deep vein thrombosis; RR, risk ratio; VTE, venous thromboembolism. Study references: [5,21–23].

Figure S36. Forest plot of any bleeding

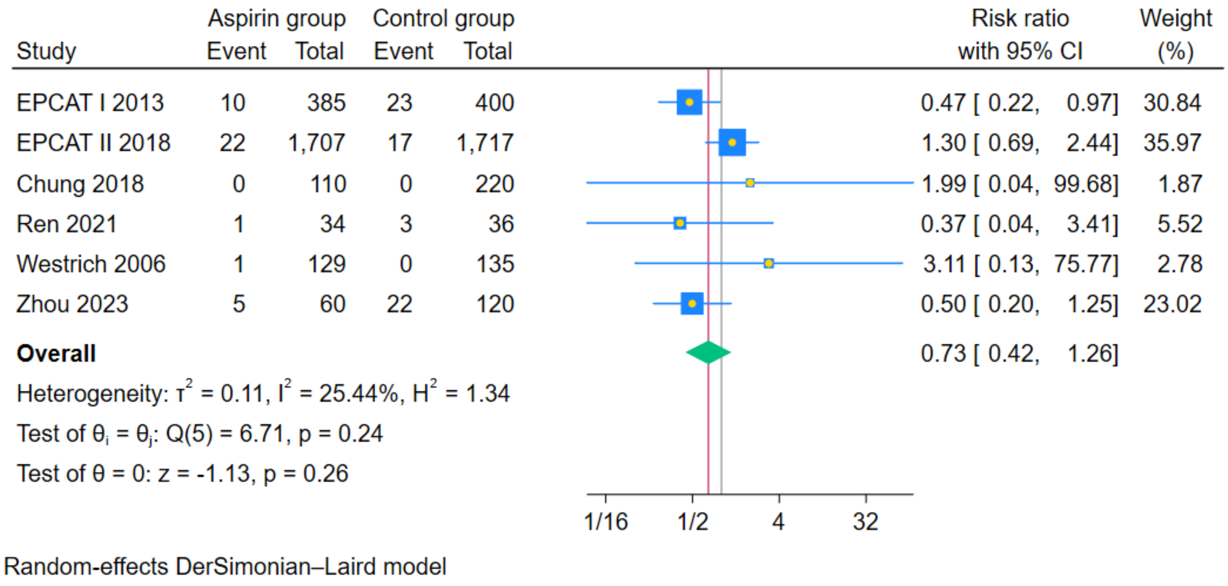

Figure S36. Forest plot of any bleeding. Pooled risk ratios were calculated using a random-effects model. CI, confidence interval; DVT, deep vein thrombosis; RR, risk ratio; VTE, venous thromboembolism. Study references: [5,6,19,21,23,28].

Figure S37. Forest plot of ecchymosis or bruising.

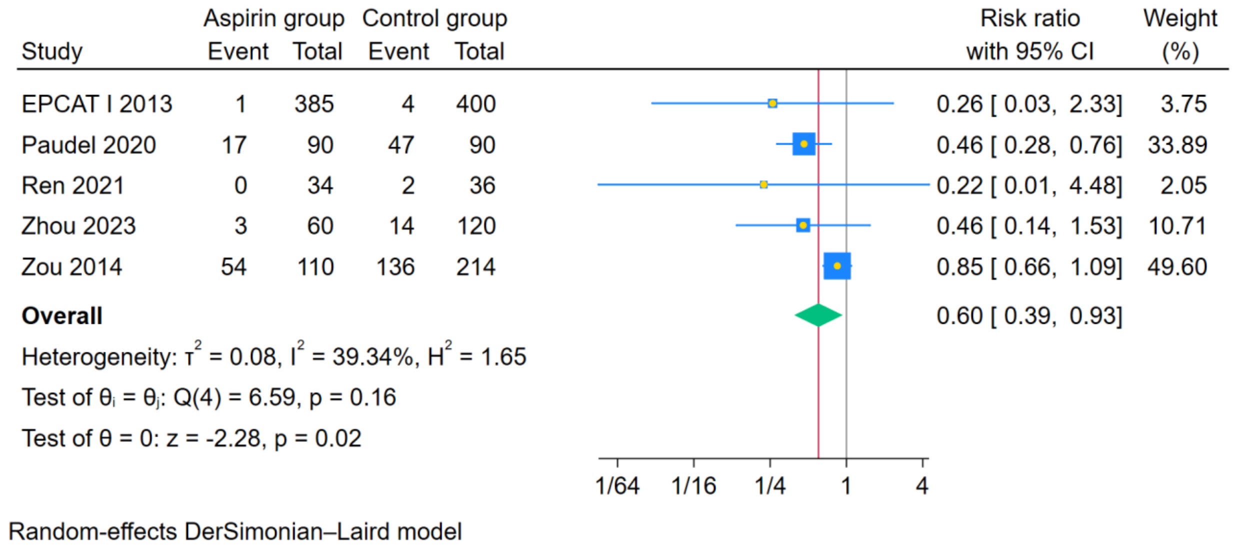

Figure S37. Forest plot of ecchymosis or bruising. Pooled risk ratios were calculated using a random-effects model. CI, confidence interval; DVT, deep vein thrombosis; RR, risk ratio; VTE, venous thromboembolism. Study references: [5,19,21,22,26].

Figure S38. Forest plot of wound infection.

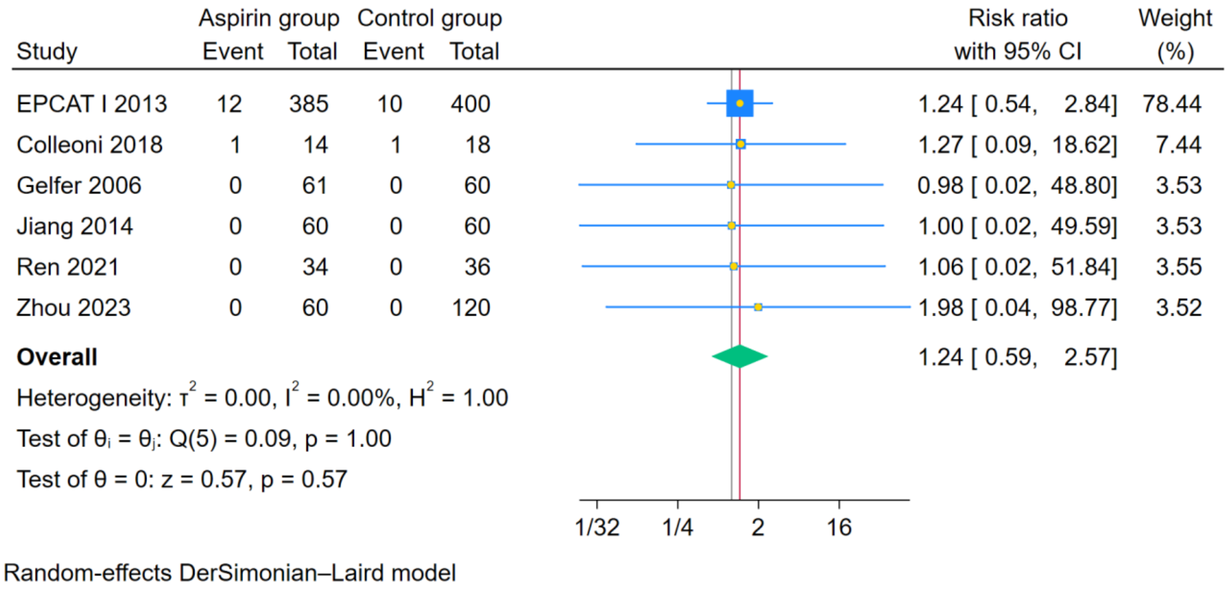

Figure S38. Forest plot of wound infection. Pooled risk ratios were calculated using a random-effects model. CI, confidence interval; DVT, deep vein thrombosis; RR, risk ratio; VTE, venous thromboembolism. Study references: [5,19,21,24,25,27].

Figure S39. Forest plot of wound hematoma.

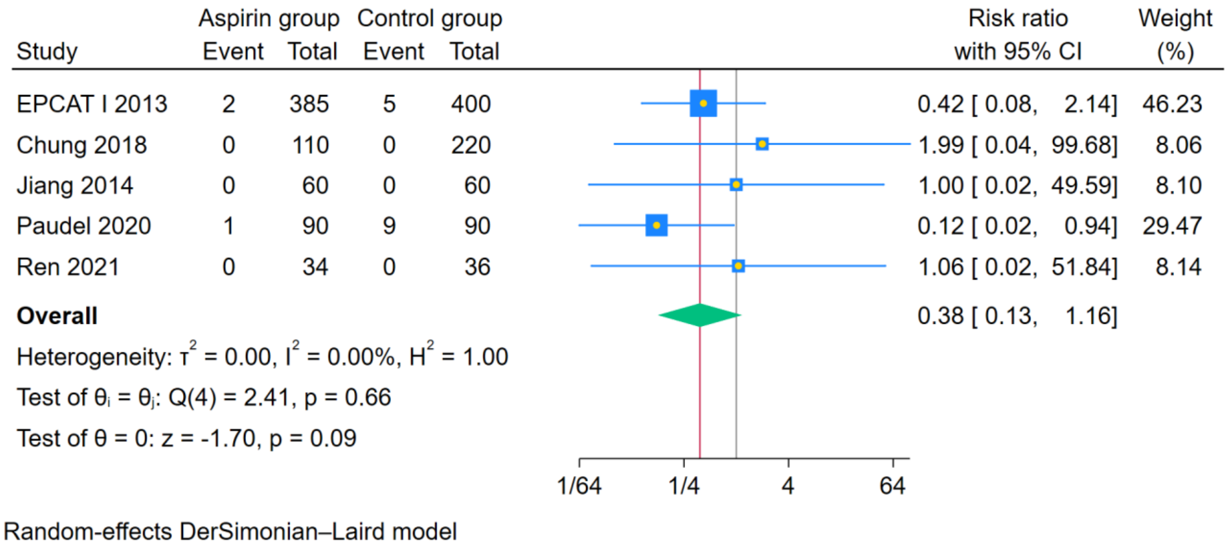

Figure S39. Forest plot of wound hematoma. Pooled risk ratios were calculated using a random-effects model. CI, confidence interval; DVT, deep vein thrombosis; RR, risk ratio; VTE, venous thromboembolism. Study references: [5,21–23,25].

Figure S40. Forest plot of gastrointestinal bleeding.

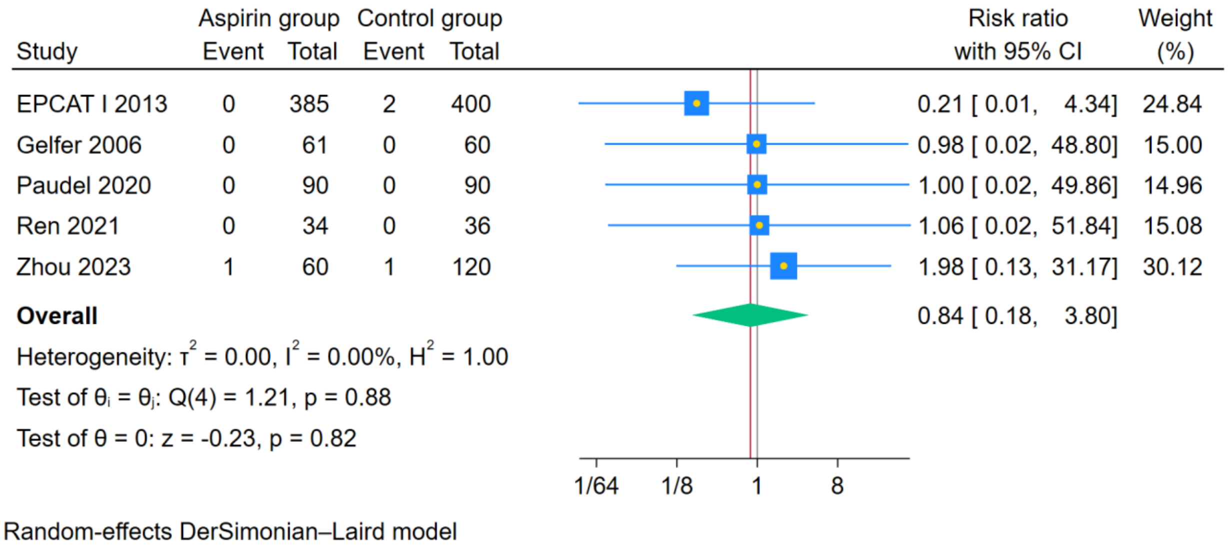

Figure S40. Forest plot of gastrointestinal bleeding. Pooled risk ratios were calculated using a random-effects model. CI, confidence interval; DVT, deep vein thrombosis; RR, risk ratio; VTE, venous thromboembolism. Study references: [5,19,21,22,27].

Figure S41. Forest plot of blood transfusion.

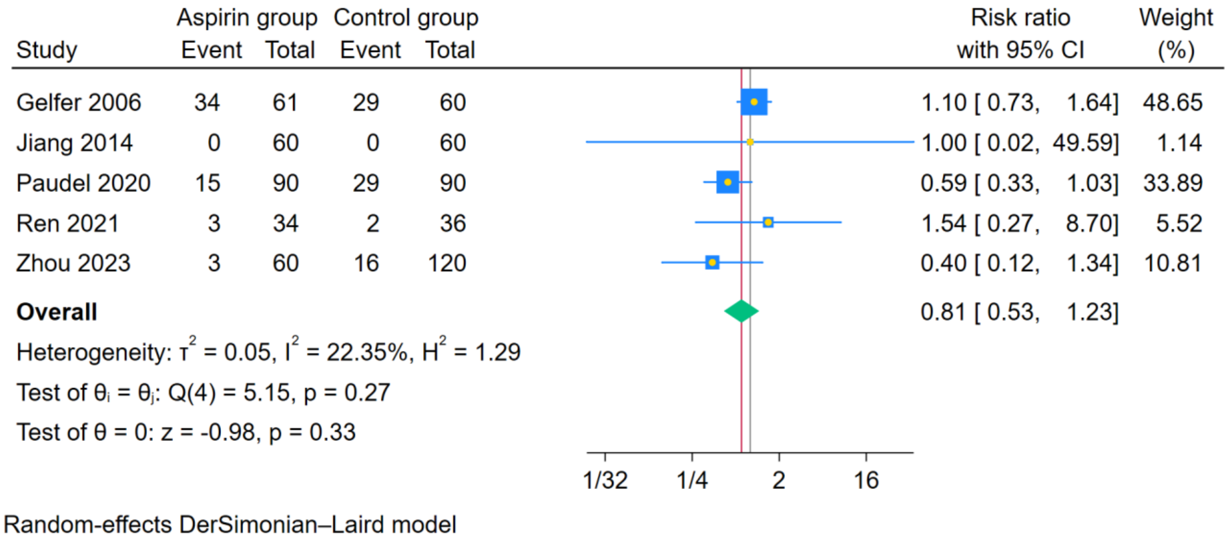

Figure S41. Forest plot of blood transfusion. Pooled risk ratios were calculated using a random-effects model. CI, confidence interval; DVT, deep vein thrombosis; RR, risk ratio; VTE, venous thromboembolism. Study references: [19,21,22,25,27].

Figure S42. Forest plot of mortality.

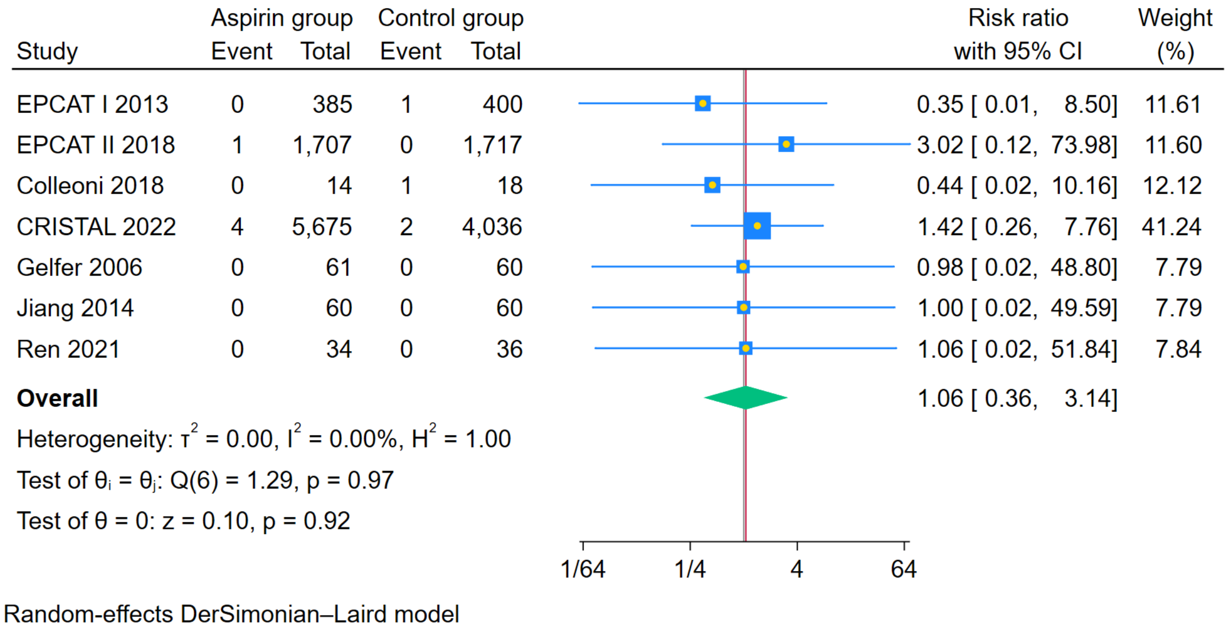

Figure S42. Forest plot of mortality. Pooled risk ratios were calculated using a random-effects model. CI, confidence interval; DVT, deep vein thrombosis; RR, risk ratio; VTE, venous thromboembolism. Study references: [1,5,6,21,24,25,27].

Low-risk-only forest plot of Symptomatic VTE

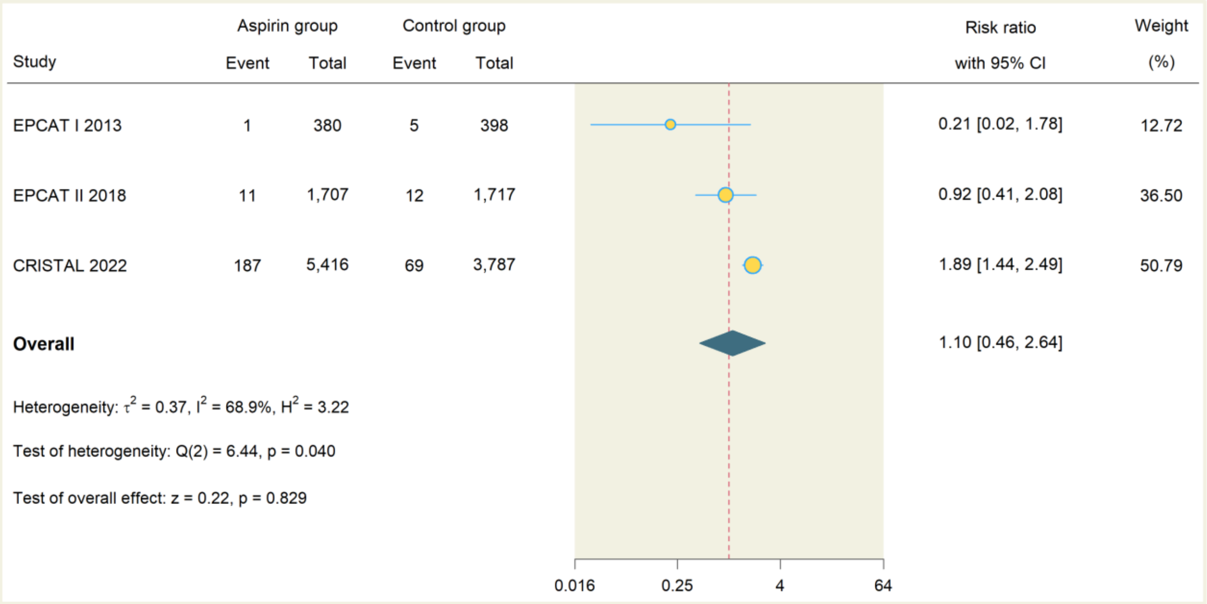

Random-effects DerSimonian-Laird model

Unadjusted for CRISTAL clustering. Trial-defined VTE outcomes differ in inclusion of calf DVT.

Figure S43. Forest plot of symptomatic venous thromboembolism restricted to trials at low risk of bias. Random-effects meta-analysis using the DerSimonian–Laird method. Trial-defined symptomatic VTE outcomes were retained. Yellow circles represent individual trial risk ratios, blue horizontal lines indicate 95% confidence intervals, and the teal diamond represents the pooled estimate. The red dashed line indicates no effect (RR = 1). RRs greater than 1 favour anticoagulant regimens. CI, confidence interval; RR, risk ratio; VTE, venous thromboembolism. Study references: [1,5,6].

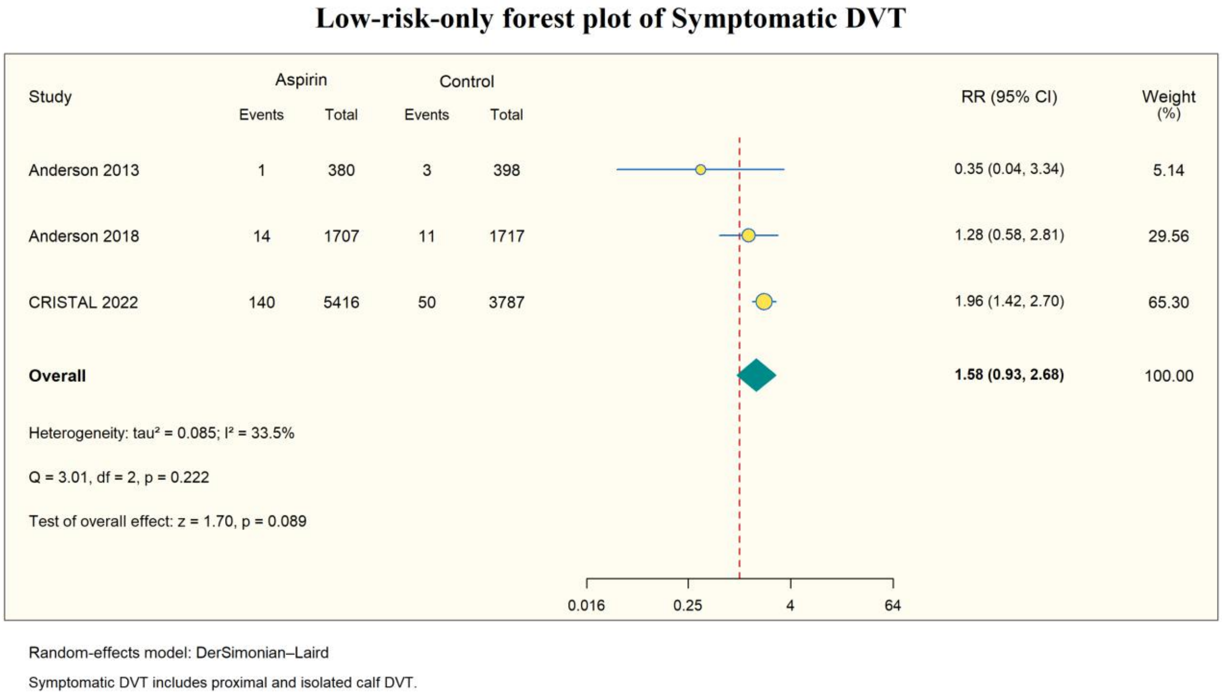

Figure S44. Forest plot of symptomatic deep vein thrombosis restricted to trials at low risk of bias. Random-effects meta-analysis, using the DerSimonian–Laird method. Symptomatic DVT includes proximal and isolated calf DVT. Yellow circles represent individual trial risk ratios, blue horizontal lines indicate 95% confidence intervals, and the teal diamond represents the pooled estimate. The red dashed line indicates no effect (RR = 1). RRs greater than 1 favour anticoagulant regimens. CI, confidence interval; DVT, deep vein thrombosis; RR, risk ratio. Study references: [1,5,6].

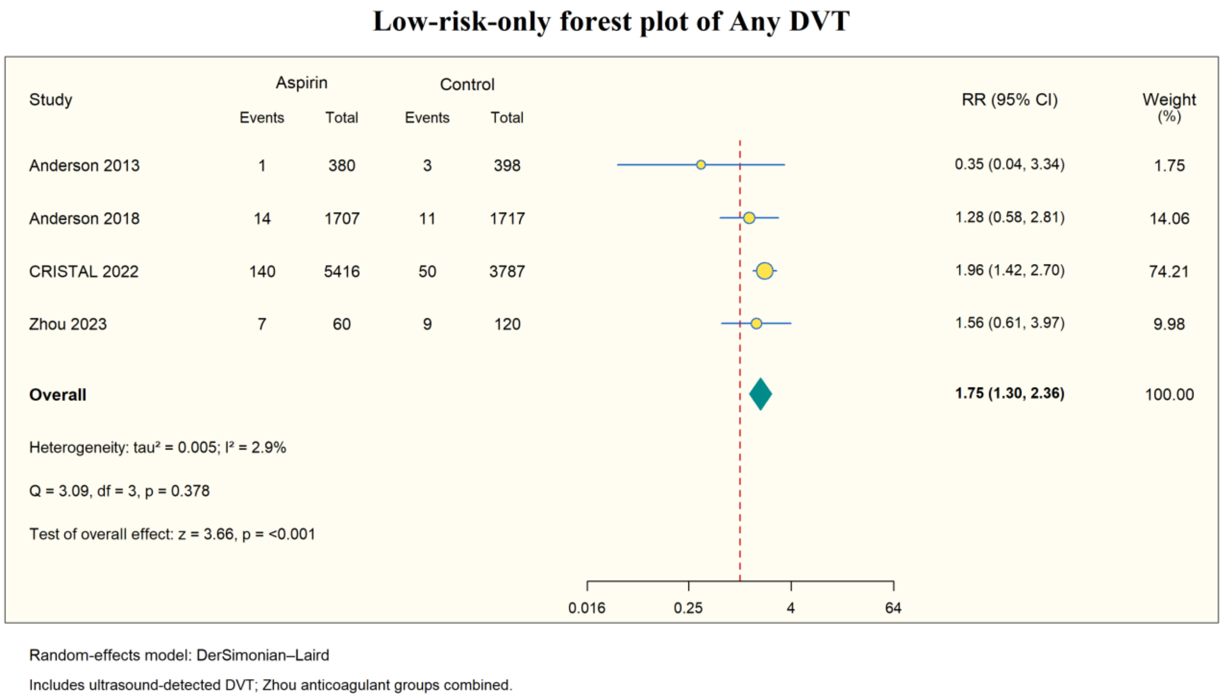

Figure S45. Forest plot of any deep vein thrombosis restricted to trials at low risk of bias. Random-effects meta-analysis, using the DerSimonian–Laird method. Any DVT includes symptomatic and screening-detected events. Zhou's rivaroxaban and dalteparin groups were combined into a single anticoagulant comparator. Yellow circles represent individual trial risk ratios, blue horizontal lines indicate 95% confidence intervals, and the teal diamond represents the pooled estimate. The red dashed line indicates no effect (RR = 1). RRs greater than 1 favour anticoagulant regimens. CI, confidence interval; DVT, deep vein thrombosis; RR, risk ratio. Study references: [1,5,6,19].

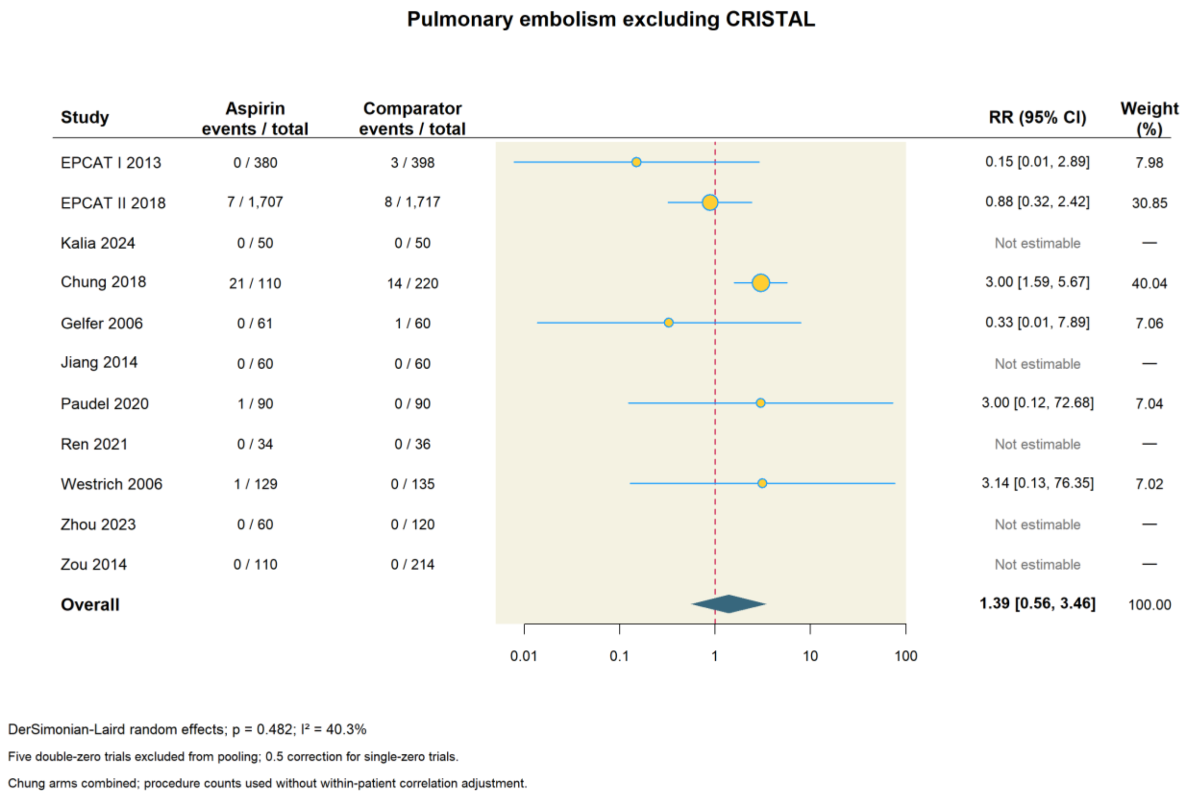

Figure S46. Forest plot of pulmonary embolism excluding the CRISTAL trial. Random-effects meta-analysis, using the DerSimonian–Laird method. PE includes symptomatic and screening-detected events. Chung's two rivaroxaban groups were combined into a single anticoagulant comparator; its procedure-based denominators were retained without adjustment for within-patient correlation. Trials with zero events in both arms are displayed but excluded from pooling. A continuity correction of 0.5 was applied to all four cells in trials with a zero-event arm. Yellow circles represent individual trial risk ratios, blue horizontal lines indicate 95% confidence intervals, and the teal diamond represents the pooled estimate. The red dashed line indicates no effect (RR = 1). RRs greater than 1 favour anticoagulant regimens. CI, confidence interval; PE, pulmonary embolism; RR, risk ratio. Study references: [5,6,19–23,25–28].

Duration classification and exclusions for Figures S47–S49

This post hoc exploratory analysis classified trials by total planned pharmacological prophylaxis duration, including any anticoagulant lead-in, provided both treatment arms fell within the same category. Chung, Colleoni, Jiang, and Zou were classified as ≤14 days [23–26]; EPCAT I, Zhou, Ren, and Westrich as >14 days [5,20,21,28]. CRISTAL, EPCAT II, and Kalia were excluded because their hip and knee regimens crossed the 14-day threshold and duration-specific event counts were unavailable in the source plots [1,6,19]. Paudel was excluded because its treatment arms fell into different categories [22], and Gelfer because its in-hospital prophylaxis duration could not be confidently classified [27]. The exclusions applied to the main pooled comparisons were retained.

Risk ratios were reconstructed from event counts and denominators displayed in Figures 3A–5A and pooled using inverse-variance DerSimonian–Laird random-effects models, with heterogeneity estimated separately within each subgroup. Double-zero trials were displayed but excluded from pooling; single-zero trials received a 0.5 correction in all four cells. Combined comparator groups were retained for Chung and Zhou; Chung's procedure-based denominators were not adjusted for within-patient correlation. Outcomes absent from the source plots were not treated as zero events. Subgroup interactions were assessed using a chi-square test with one degree of freedom.

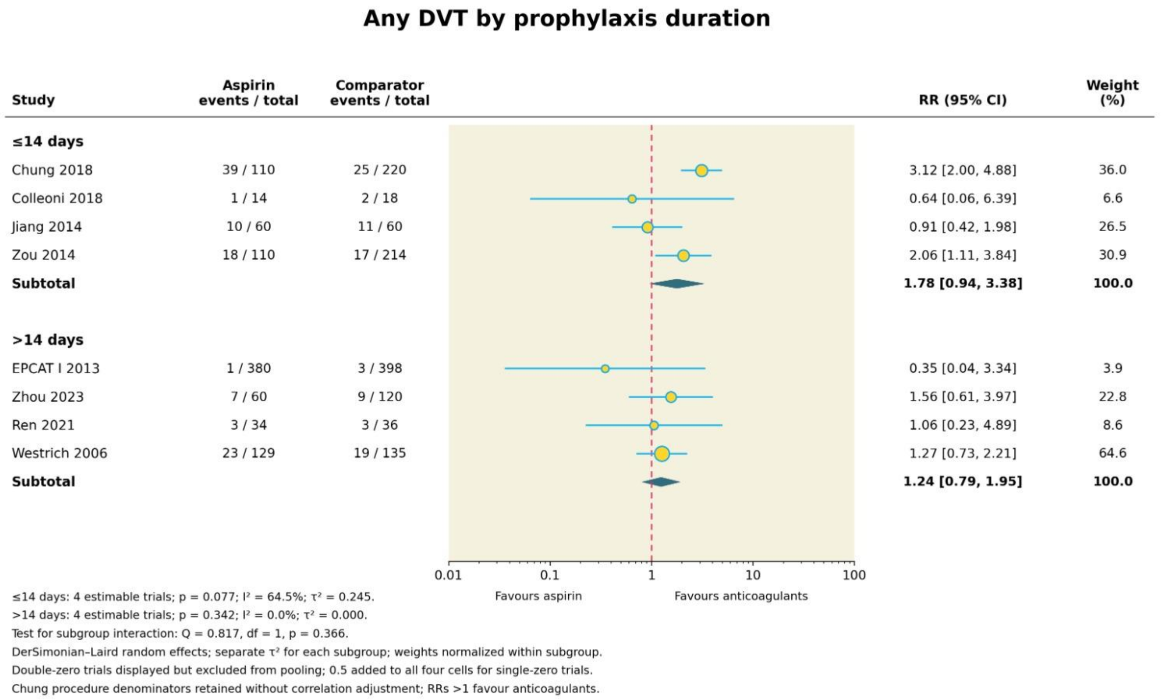

Figure S47. Forest plot of any deep vein thrombosis stratified by total pharmacological prophylaxis duration (≤14 versus >14 days). Any DVT includes symptomatic and screening-detected events. Eight trials contributed estimable risk ratios. Zhou's rivaroxaban and dalteparin groups were combined into a single anticoagulant comparator. Study references: [5,19,21,23–26,28].

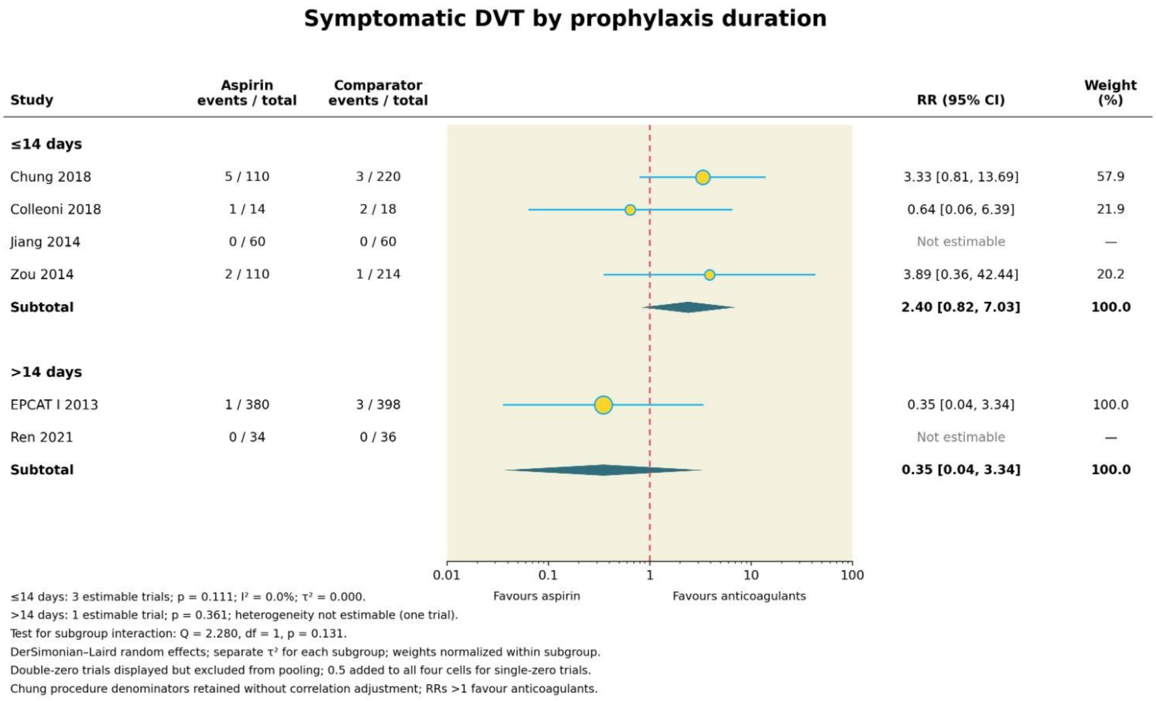

Figure S48. Forest plot of symptomatic deep vein thrombosis stratified by total pharmacological prophylaxis duration (≤14 versus >14 days). Six trials were displayed and four contributed estimable risk ratios. Jiang and Ren had zero events in both arms and did not contribute to pooling. EPCAT I was the only informative trial in the >14-day subgroup. Outcomes not displayed in the source symptomatic-DVT plot were not entered as zero. Study references: [5,21,23–26].

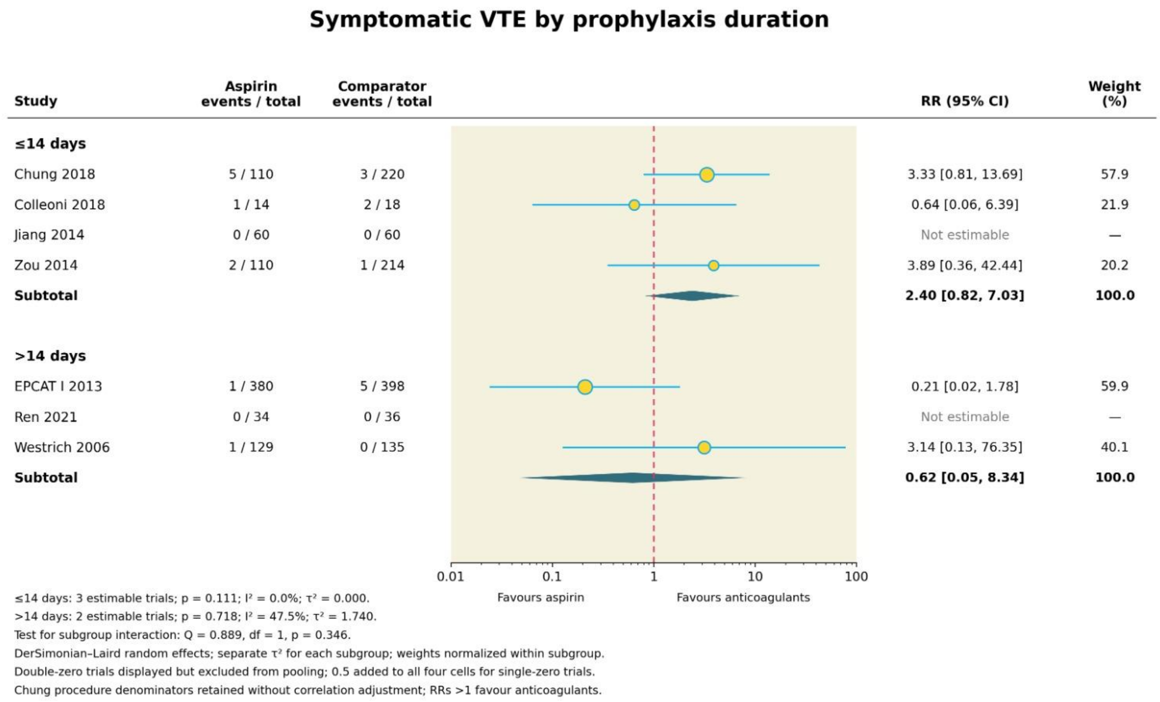

Figure S49. Forest plot of symptomatic venous thromboembolism stratified by total pharmacological prophylaxis duration (≤14 versus >14 days). Seven trials were displayed and five contributed estimable risk ratios. Trial-defined symptomatic VTE outcomes were retained. Jiang and Ren had zero events in both arms and did not contribute to pooling. Outcomes not displayed in the source symptomatic-VTE plot were not entered as zero. Study references: [5,21,23–26,28].

CRISTAL-adjusted sensitivity analyses for Figures S50–S53

These post hoc sensitivity analyses used generic inverse-variance random-effects pooling of absolute risk differences, with between-study variance estimated by restricted maximum likelihood (REML) and normal 95% confidence intervals. CRISTAL contributed its published cluster-adjusted risk difference and a standard error approximated from its reported 95% confidence interval [1]. The same CRISTAL DVT estimate was used for any DVT and symptomatic DVT because asymptomatic patients were not screened.

For other trials, risk differences and independent-binomial variances were reconstructed from the event counts in the main forest plots. A correction of 0.5 was added to all four cells of trials with a zero-event arm; double-zero trials were retained. Chung's comparator arms were combined; its procedure-based denominators were retained without adjustment for within-patient correlation. These analyses differ from the main risk-ratio analyses in both effect measure and pooling method. Risk-difference pooling is sensitive to sparse events and differences in baseline risk; these results do not establish equivalence.

Figure S50. Any DVT with CRISTAL cluster adjustment

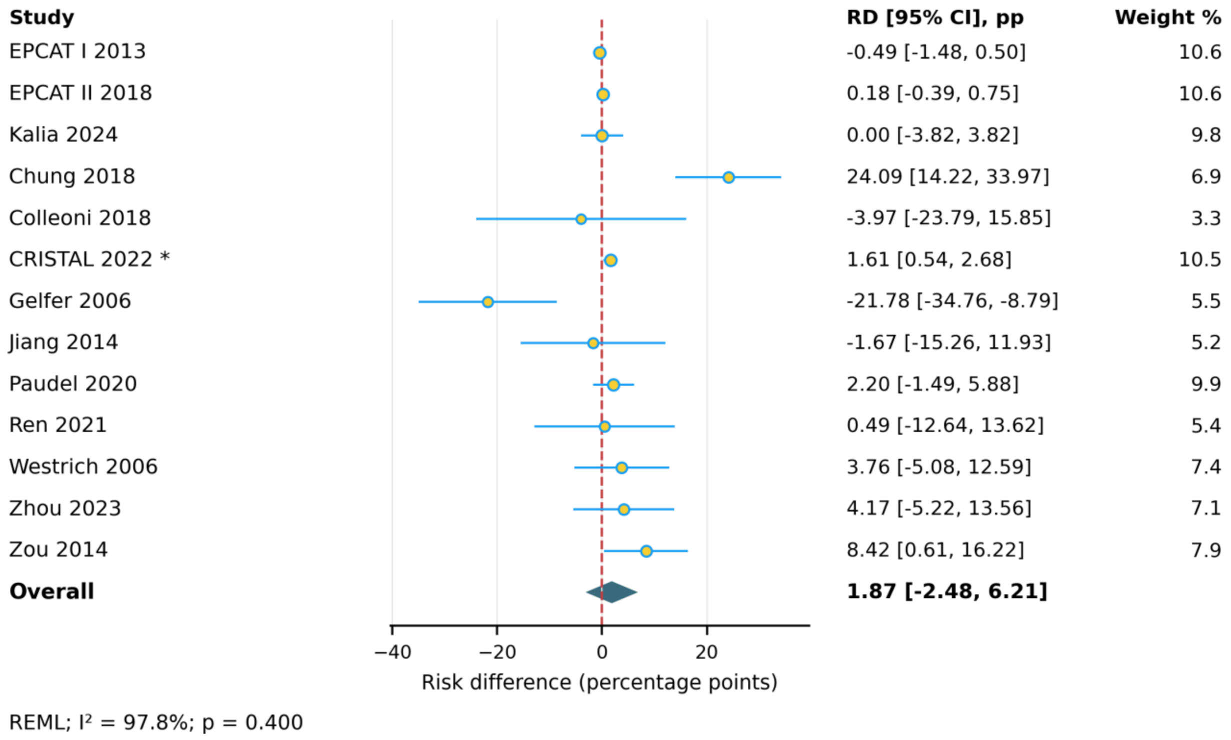

Figure S50. Forest plot of any deep vein thrombosis using CRISTAL's published cluster-adjusted estimate in a post hoc risk-difference sensitivity analysis. Any DVT includes symptomatic and screening-detected events. Yellow circles represent trial risk differences, blue lines their 95% confidence intervals, and the teal diamond the pooled estimate. The red dashed line indicates no difference (RD = 0). Positive values favour anticoagulant regimens. All effects are absolute percentage-point differences. *CRISTAL: published cluster-adjusted estimate; other trials use reconstructed counts. CI, confidence interval; DVT, deep vein thrombosis; PE, pulmonary embolism; pp, percentage points; RD, risk difference; VTE, venous thromboembolism. Study references: [1,5,6,19–28].

Figure S51. Symptomatic DVT with CRISTAL cluster adjustment

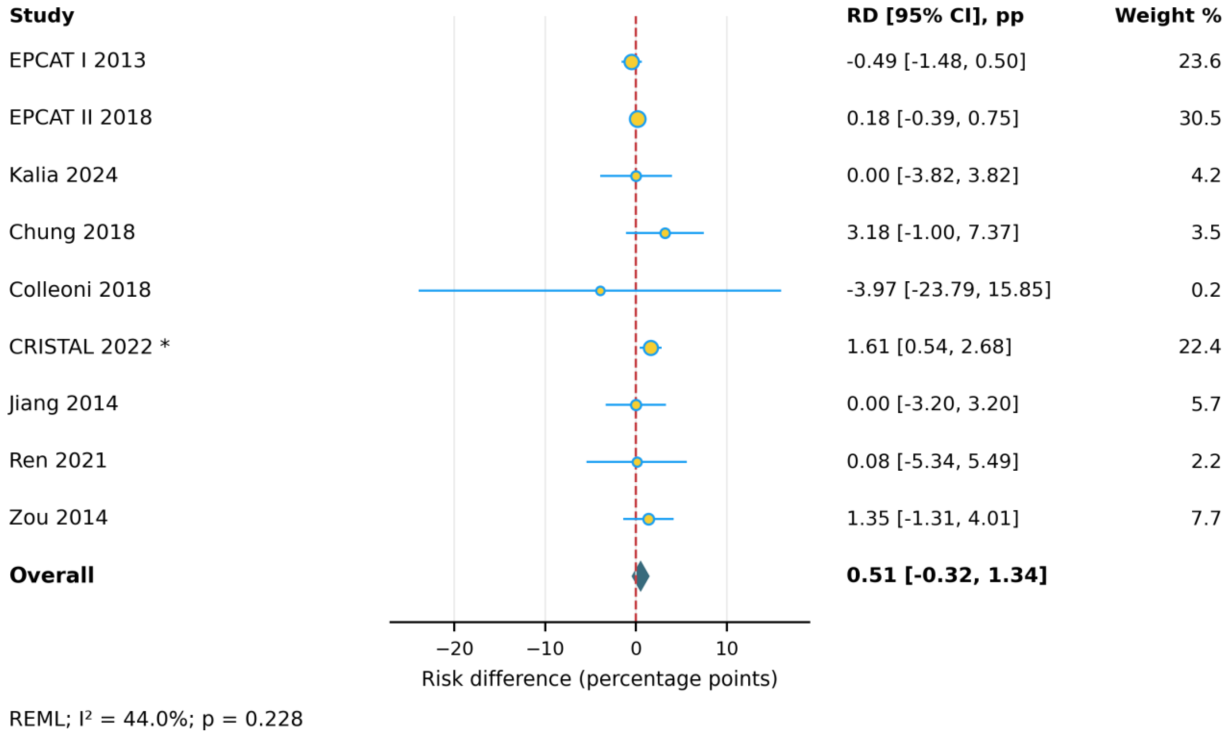

Figure S51. Forest plot of symptomatic deep vein thrombosis using CRISTAL's published cluster-adjusted estimate in a post hoc risk-difference sensitivity analysis. Yellow circles represent trial risk differences, blue lines their 95% confidence intervals, and the teal diamond the pooled estimate. The red dashed line indicates no difference (RD = 0). Positive values favour anticoagulant regimens. All effects are absolute percentage-point differences. *CRISTAL: published cluster-adjusted estimate; other trials use reconstructed counts. CI, confidence interval; DVT, deep vein thrombosis; PE, pulmonary embolism; pp, percentage points; RD, risk difference; VTE, venous thromboembolism. Study references: [1,5,6,20,21,23–26].

Figure S52. Symptomatic VTE with CRISTAL cluster adjustment

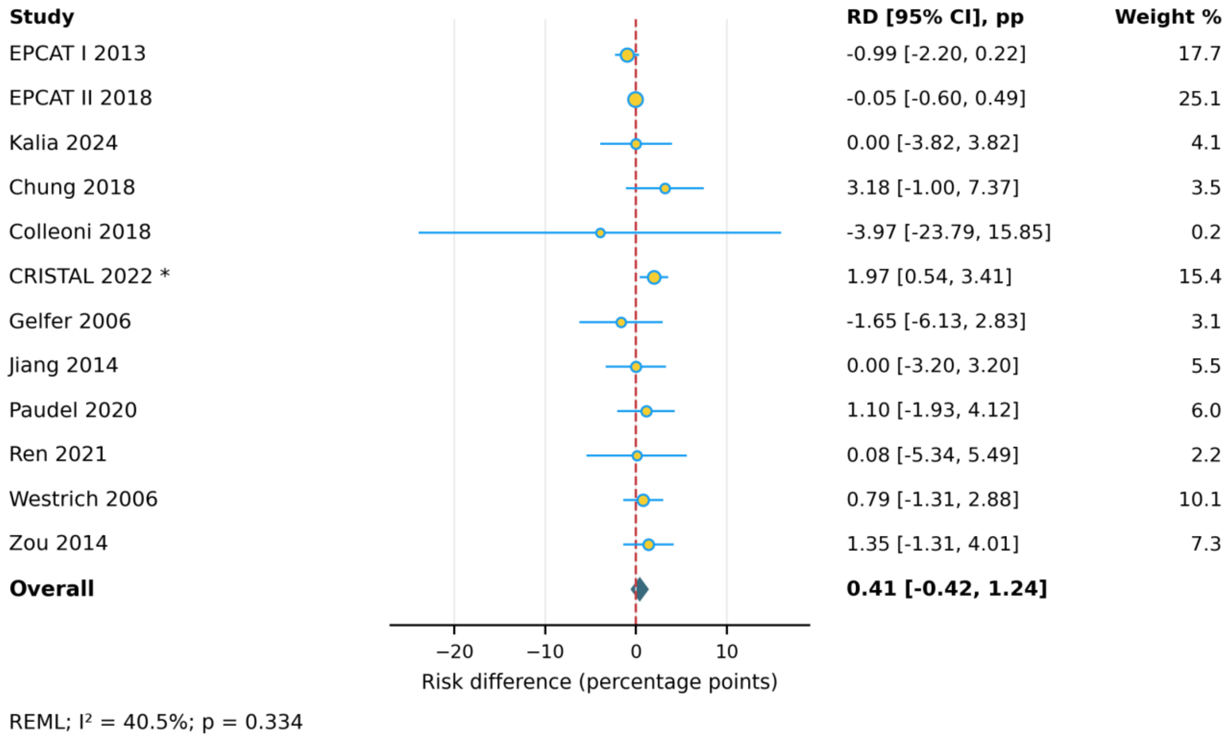

Figure S52. Forest plot of symptomatic venous thromboembolism using CRISTAL's published cluster-adjusted estimate in a post hoc risk-difference sensitivity analysis. Trial-defined symptomatic VTE outcomes were retained. Yellow circles represent trial risk differences, blue lines their 95% confidence intervals, and the teal diamond the pooled estimate. The red dashed line indicates no difference (RD = 0). Positive values favour anticoagulant regimens. All effects are absolute percentage-point differences. *CRISTAL: published cluster-adjusted estimate; other trials use reconstructed counts. CI, confidence interval; DVT, deep vein thrombosis; PE, pulmonary embolism; pp, percentage points; RD, risk difference; VTE, venous thromboembolism. Study references: [1,5,6,20,21–28].

Figure S53. PE with CRISTAL cluster adjustment

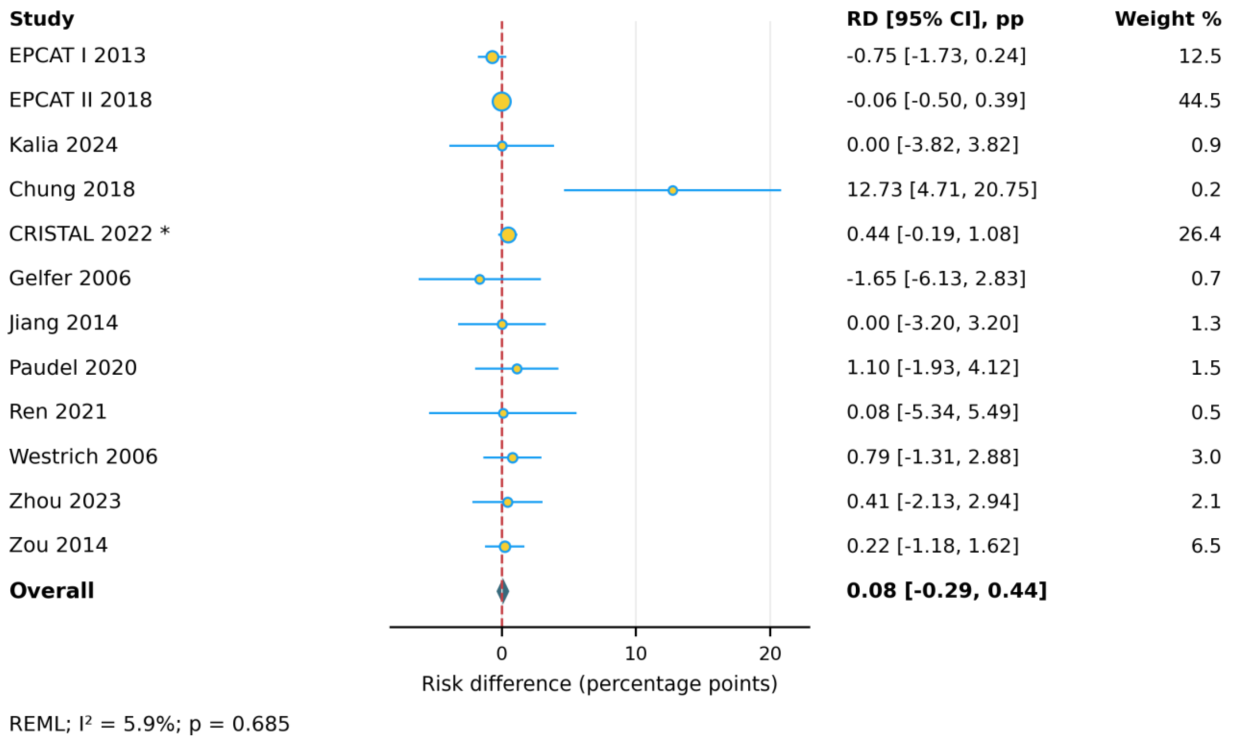

Figure S53. Forest plot of pulmonary embolism using CRISTAL's published cluster-adjusted estimate in a post hoc risk-difference sensitivity analysis. PE includes symptomatic and screening-detected events. Yellow circles represent trial risk differences, blue lines their 95% confidence intervals, and the teal diamond the pooled estimate. The red dashed line indicates no difference (RD = 0). Positive values favour anticoagulant regimens. All effects are absolute percentage-point differences. *CRISTAL: published cluster-adjusted estimate; other trials use reconstructed counts. CI, confidence interval; DVT, deep vein thrombosis; PE, pulmonary embolism; pp, percentage points; RD, risk difference; VTE, venous thromboembolism. Study references: [1,5,6,19–23,25–28].
